# Analysis of the *Phlebiopsis gigantea* Genome, Transcriptome and Secretome Provides Insight into Its Pioneer Colonization Strategies of Wood

**DOI:** 10.1371/journal.pgen.1004759

**Published:** 2014-12-04

**Authors:** Chiaki Hori, Takuya Ishida, Kiyohiko Igarashi, Masahiro Samejima, Hitoshi Suzuki, Emma Master, Patricia Ferreira, Francisco J. Ruiz-Dueñas, Benjamin Held, Paulo Canessa, Luis F. Larrondo, Monika Schmoll, Irina S. Druzhinina, Christian P. Kubicek, Jill A. Gaskell, Phil Kersten, Franz St. John, Jeremy Glasner, Grzegorz Sabat, Sandra Splinter BonDurant, Khajamohiddin Syed, Jagjit Yadav, Anthony C. Mgbeahuruike, Andriy Kovalchuk, Fred O. Asiegbu, Gerald Lackner, Dirk Hoffmeister, Jorge Rencoret, Ana Gutiérrez, Hui Sun, Erika Lindquist, Kerrie Barry, Robert Riley, Igor V. Grigoriev, Bernard Henrissat, Ursula Kües, Randy M. Berka, Angel T. Martínez, Sarah F. Covert, Robert A. Blanchette, Daniel Cullen

**Affiliations:** 1 Department of Biomaterials Sciences, University of Tokyo, Tokyo, Japan; 2 Department of Chemical Engineering, University of Toronto, Toronto, Ontario, Canada; 3 Department of Biochemistry and Molecular and Cellular Biology and Institute of Biocomputation and Physics of Complex Systems, University of Zaragoza, Zaragoza, Spain; 4 Centro de Investigaciones Biológicas, Consejo Superior de Investigaciones Cientificas, Madrid, Spain; 5 Department of Plant Pathology, University of Minnesota, St. Paul, Minnesota, United States of America; 6 Millennium Nucleus for Fungal Integrative and Synthetic Biology and Departamento de Genética Molecular y Microbiología, Facultad de Ciencias Biológicas, Pontificia Universidad Católica de Chile, Santiago, Chile; 7 Health and Environment Department, Austrian Institute of Technology GmbH, Tulin, Austria; 8 Austrian Center of Industrial Biotechnology and Institute of Chemical Engineering, Vienna University of Technology, Vienna, Austria; 9 USDA, Forest Products Laboratory, Madison, Wisconsin, United States of America; 10 University of Wisconsin Biotechnology Center, Madison, Wisconsin, United States of America; 11 Department of Environmental Health, University of Cincinnati, Cincinnati, Ohio, United States of America; 12 Department of Forest Sciences, University of Helsinki, Helsinki, Finland; 13 Department of Pharmaceutical Biology at the Hans-Knöll-Institute, Friedrich-Schiller-University, Jena, Germany; 14 Instituto de Recursos Naturales y Agrobiologia de Sevilla, CSIC, Seville, Spain; 15 US Department of Energy Joint Genome Institute, Walnut Creek, California, United States of America; 16 Architecture et Fonction des Macromolécules Biologiques, Unité Mixte de Recherche 7257, Aix-Marseille Université, Centre National de la Recherche Scientifique, Marseille, France; 17 Molecular Wood Biotechnology and Technical Mycology, Büsgen-Institute, Georg-August University Göttingen, Göttingen, Germany; 18 Novozymes, Inc., Davis, California, United States of America; 19 Warnell School of Forestry and Natural Resources, University of Georgia, Athens, Georgia, United States of America; The University of North Carolina at Chapel Hill, United States of America

## Abstract

Collectively classified as white-rot fungi, certain basidiomycetes efficiently degrade the major structural polymers of wood cell walls. A small subset of these Agaricomycetes, exemplified by *Phlebiopsis gigantea*, is capable of colonizing freshly exposed conifer sapwood despite its high content of extractives, which retards the establishment of other fungal species. The mechanism(s) by which *P. gigantea* tolerates and metabolizes resinous compounds have not been explored. Here, we report the annotated *P. gigantea* genome and compare profiles of its transcriptome and secretome when cultured on fresh-cut versus solvent-extracted loblolly pine wood. The *P. gigantea* genome contains a conventional repertoire of hydrolase genes involved in cellulose/hemicellulose degradation, whose patterns of expression were relatively unperturbed by the absence of extractives. The expression of genes typically ascribed to lignin degradation was also largely unaffected. In contrast, genes likely involved in the transformation and detoxification of wood extractives were highly induced in its presence. Their products included an ABC transporter, lipases, cytochrome P450s, glutathione S-transferase and aldehyde dehydrogenase. Other regulated genes of unknown function and several constitutively expressed genes are also likely involved in *P. gigantea*'s extractives metabolism. These results contribute to our fundamental understanding of pioneer colonization of conifer wood and provide insight into the diverse chemistries employed by fungi in carbon cycling processes.

## Author Summary

The wood decay fungus *Phlebiopsis gigantea* degrades all components of plant cell walls and is uniquely able to rapidly colonize freshly exposed conifer sapwood. However, mechanisms underlying its conversion of lignocellulose and resinous extractives have not been explored. We report here analyses of the genetic repertoire, transcriptome and secretome of *P. gigantea*. Numerous highly expressed hydrolases, together with lytic polysaccharide monooxygenases were implicated in *P. gigantea*'*s* attack on cellulose, and an array of ligninolytic peroxidases and auxiliary enzymes were also identified. Comparisons of woody substrates with and without extractives revealed differentially expressed genes predicted to be involved in the transformation of resin. These expression patterns are likely key to the pioneer colonization of conifers by *P. gigantea*.

## Introduction

The most abundant source of terrestrial carbon is plant biomass, composed primarily of cellulose, hemicellulose, and lignin. Numerous microbes utilize cellulose and hemicellulose, but a much smaller group of filamentous fungi has the capacity to degrade lignin, the most recalcitrant component of plant cell walls. Uniquely, such ‘white-rot’ fungi efficiently depolymerize lignin to access cell wall carbohydrates for carbon and energy sources. As such, white-rot fungi play a key role in the carbon cycle.

**Figure 1:**
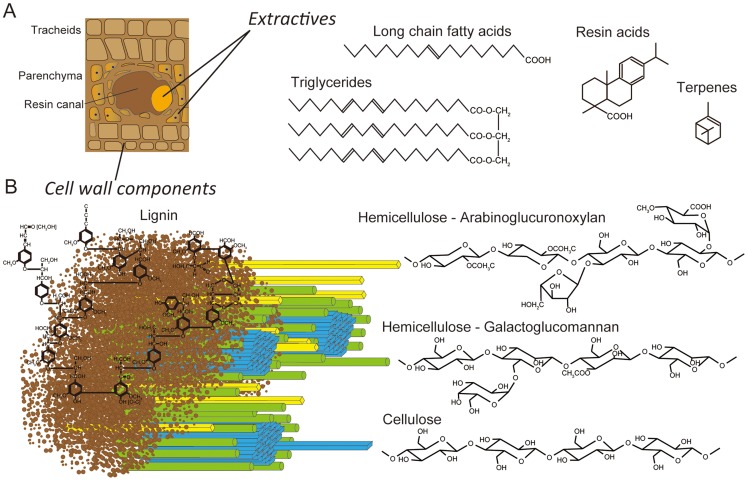
Schematic representations of lignocellulose components in cell walls of pine wood. Panel A: The extractives (long chain fatty acids, triglycerides, resin acids and terpenes) are found primarily in the resin ducts, but damage to pine wood causes the release of these compounds across wounded areas. Panel B: In tracheid cell walls, the amorphous, phenylpropanoid polymer lignin (brown) form a matrix around the more structured carbohydrate polymers, hemicellulose (yellow and green) and cellulose (blue).

White-rot basidiomycetes may differ in their substrate preference and morphological patterns of decay (for review see [1], [2]). The majority of lignin-degrading fungi, including *Phanerochaete chrysosporium* and *Ceriporiopsis subvermispora*, are unable to colonize freshly cut wood unless inhibitory compounds (extractives) are removed or transformed [2]–[5]. A few basidiomycetes, including *Phlebiopsis gigantea*, are pioneer colonizers of softwood because they tolerate and utilize resinous extractives (e.g., resin acids, triglycerides, long chain fatty acids, see Figure 1) which cause pitch deposits in paper pulp manufacturing [6]. It is this unusual capability that also led to the development of *P. gigantea* as a biocontrol agent against subsequent colonization of cut stumps by the root rot pathogen *Heterobasidium annosum* sensu lato (now considered several species) [7], [8] and of harvested wood by blue stain fungi [9], [10]. It seems likely that when applied to freshly cut wood, *P. gigantea* is able to rapidly metabolize accessible extractives and hemicellulose. As the hyphae continue to invade tracheids and ray parenchyma cells, the more recalcitrant cell wall polymers (cellulose, lignin; Figure 1) are eroded. Little is known of how some white-rot fungi degrade conifer extractives [11], [12] or interact with other fungi such as *H. annosum* [13].

White-rot fungi degrade major cell wall polymers through concerted action of hydrolytic and oxidative enzymes (reviewed in [14], [15]). Cellulose is attacked by a combination of exo-cellobiohydrolases and endoglucanases assigned to glycoside hydrolase families GH5, GH6, GH7 and possibly GH9, GH12, GH44 and GH45 [16], [17]. In addition to these hydrolases, recent evidence strongly supports the involvement of lytic polysaccharide monooxygenases (LPMOs) in cellulose degradation [18]–[20]. Lignin degradation is catalyzed by an array of oxidative enzymes, especially lignin peroxidase (LiP), manganese peroxidase (MnP) and versatile peroxidase (VP) belonging to class II of the plant-fungal-prokaryotic peroxidase superfamily. Recent genome investigations reveal that all efficient lignin degraders possess some combination of these class II ligninolytic peroxidases [21], [22]. In *P. gigantea*, four MnP sequences were previously identified [23].

In addition to peroxidases, laccases have been implicated in lignin degradation [24]–[26]. To date, multiple laccase isozymes and/or the corresponding genes have been characterized from most white-rot fungi except *P. chrysosporium*, an efficient lignocellulose degrader that lacks such enzymes [27]–[29]. The mechanism(s) by which laccases might degrade lignin remain unclear as the enzyme lacks sufficient oxidation potential to cleave non-phenolic linkages within the polymer. Interestingly, laccase activity has not been reported in *P. gigantea*.

Additional ‘auxiliary activities’ [30] commonly ascribed to ligninolytic systems include extracellular enzymes capable of generating H_2_O_2_. These enzymes may be physiologically coupled to peroxidases. Among them, aryl-alcohol oxidase (AAO), methanol oxidase (MOX), pyranose 2-oxidase (P2O), and copper radical oxidases (such as glyoxal oxidase, GLX) have been extensively studied. With the exception of P2O [31], none of these activities have been reported in *P. gigantea* cultures. In short, the repertoire of extracellular enzymes produced by *P. gigantea* is largely unknown, and its mechanism(s) for cell wall degradation remain unexplored.

Beyond extracellular systems, the complete degradation of lignin requires many intracellular enzymes for the complete mineralization of monomers to CO_2_ and H_2_O. Examples of enzymes that have been characterized from *P. chrysosporium* include cytochromes P450 (CYPs) [32]–[34], glutathione transferases [35], and aryl alcohol dehydrogenase (AAD) [36]. The role of such enzymes in *P. gigantea*, if any, is unknown.

Herein, we report analysis of the *P. gigantea* draft genome. Gene annotation, transcriptome analyses and secretome profiles identified numerous genes involved in lignocellulose degradation and in the metabolism of conifer extractives.

## Results

### Genome assembly and annotation

**Figure 2:**
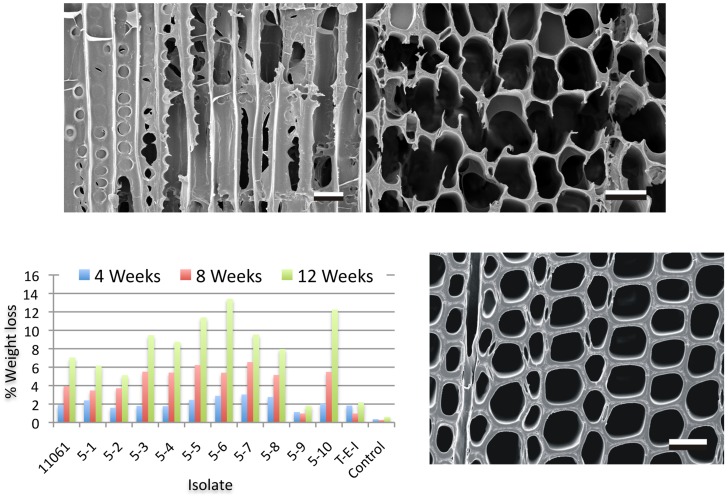
Wood decay characteristics. Comparative weight loss of parental strain 11061 and single basidiospore derivatives on colonized loblolly pine wood (*Pinus taeda*) wood wafers were determined after 4, 8 and 12 weeks incubation (bottom left panel) as described in Methods. Single basidiospore strain 5–6 also aggressively decayed birch and spruce (Text S1) and was selected for sequencing. Upper panels show scanning electron microscopy [68] of radial (left) and transverse (right) sections of pine wood tracheids that were substantially eroded or completely degraded by *P. gigantea* strain 5–6 by week twelve. Transverse section of sound wood (bottom photo) provides comparison. (Bar  = 40 µm).

Following an assessment of wood decay properties (Figure 2), *P. gigantea* single basidiospore strain 5–6 was selected for sequencing using Illumina reads assembled with AllPathsLG. Genome size was estimated to be approximately 30 Mbp (Text S1), somewhat lower than closely related members of the ‘*Phlebia* clade’ [23], [37] such as *C. subvermispora* (39 Mbp) and *P. chrysosporium* (35 Mbp) [22], [27]. Aided by 17,915 mapped EST clusters, the JGI annotation pipeline predicted 11,891 genes. Proteins were assigned to 6412, 5615, 6932 and 2253 KOG categories, GO terms, pfam domains and EC numbers, respectively. Significant synteny with *P. chrysosporium* was observed (Figure S1). Detailed information on the assembly and annotations is available via the JGI portal MycoCosm [38].

### Gene families

**Figure 3:**
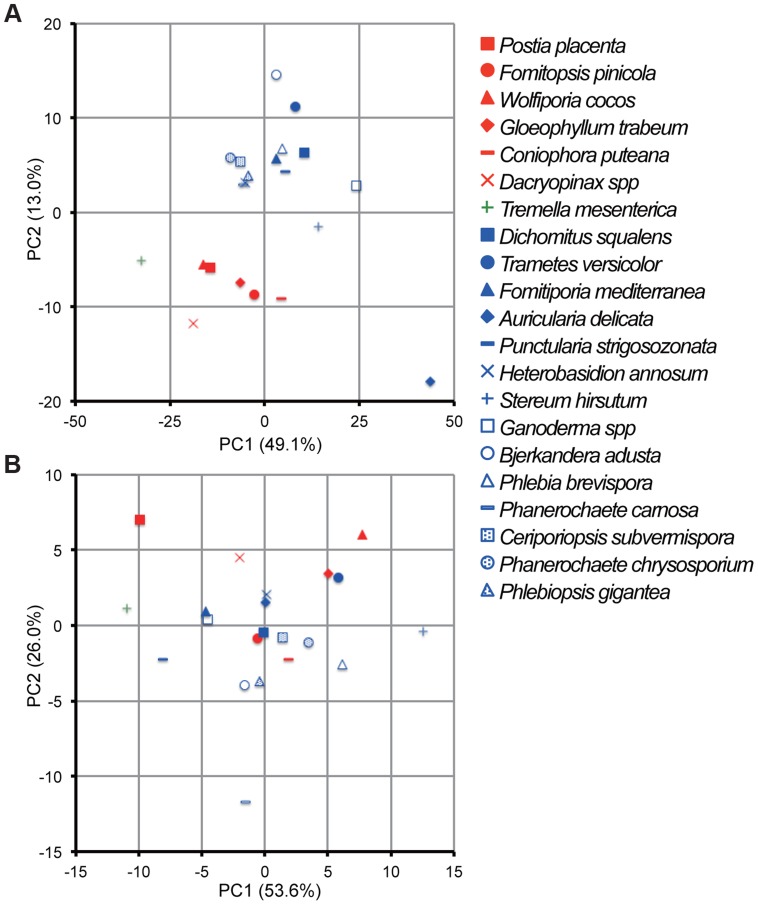
Comparative analysis of gene repertoires associated with degradation of plant cell wall polymers and extractives in 21 fungal genomes. (A) Principal component analysis (PCA) of 21 fungi using 73 CAZy and 12 AA families (Dataset S1). GMC oxidoreductases methanol oxidase, glucose oxidase and aryl alcohol oxidase were excluded because confident functional assignments could not be made and/or their inclusion did not contribute to separation of white- and brown-rot species. (B) PCA of 21 fungi using genes encoding 14 enzymes involved in lipid metabolism (KEGG reference pathway 00071, Dataset S1). There is no significant segregation of white-rot and brown-rot fungi although *P. gigantea* was positioned in the third quadrant with *B. adusta* and *P. carnosa*. Symbols for white rot and brown rot fungi appear in blue and red, respectively. *Tremella mesenterica* is a mycoparasite. For raw data and contributions of the top 20 families see Dataset S1, Text S1 and Figures S2 and S3.

**Figure 4:**
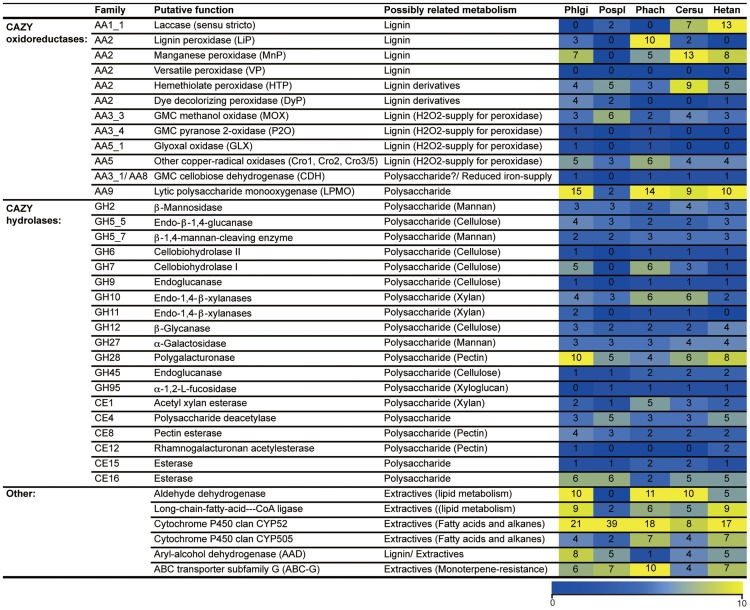
Number of genes identified in white rot fungi *P. gigantea* (Phlgi), *P. chrysosporium* (Phach)[27], *C. subvermispora* (Cersu)[22], and *H. annosum* (Hetan)[75], and the brown rot fungus *P. placenta* (Pospl)[45]. CROs were distinguished as previously described [76]. Lytic polysaccharide monooxygenases were formerly classified as GH61 within the CAZy system (http://www.cazy.org/; [16]). Glycoside hydrolase family GH5 was subdivided as described [77] (Figure S22).

Principal component analysis (PCA), based on 73 and 12 families of carbohydrate active enzymes (CAZys, [16]) and auxiliary activities (AAs), [30]), respectively, clustered *P. gigantea* with other efficient lignin degraders ([39], Figures 3A and S2). Gene numbers were extracted from 21 fungal genomes and excluded genes encoding putative GMC oxidases such as methanol oxidase, alcohol oxidase and glucose oxidase (Dataset S1). Highest contribution of PC1 (50% of variance separating white-rot and brown-rot fungi) and PC2 (13.0% of variance)) values were those genes associated with degradation of plant cell wall polysaccharides and lignin, respectively (Text S1). Hierarchical clustering analysis with this dataset also categorized *P. gigantea* into a clade of white-rot fungi that included the polypore *P. chrysosporium*. The precise number and distribution of *P. gigantea* genes likely involved in lignocellulose degradation were similar, but not identical, to other polypores such as *P. chrysosporium* and *C. subvermispora* (Figure 4). Like *P. chrysosporium* and *Phanerochaete flavido-alba*, *P. gigantea* had no laccase *sensu stricto* genes. Interestingly, while both *P. gigantea* and the white-rot Russulales *H. annosum* are adapted to colonization of conifers, significant numbers of laccase *sensu stricto* genes were only observed in *H. annosum* (Figure 4). This important conifer pathogen also lacked GLX, LiP and representatives of GH5 subfamiles 15 and 31.

With regard to hemicellulose degradation, the genomes of conifer-adapted *P. gigantea* and *H. annosum* revealed increased numbers of genes involved in pectin degradation such as GH28 polygalacturonase, CE8 pectin methylesterase and CE12 rhamnogalacturonan acetylesterase (Figure 4). The major hemicellulose of conifer is galactoglucomannan ([40], Figure 1) but, in the case of mannan degradation, no significant increase in genes encoding GH2 β-mannosidase, GH5_7 endo-mannanase and GH27 α-galactosidase was observed relative to other wood decay fungi (Figure 4). Similarly, no significant differences in the number of genes involved in arabinoglucuronoxylan hydrolysis were identified, except for two transcriptionally convergent GH11 genes present in *P. gigantea* (Text S1). Encoding putative endo-1,4-β-xylanases, wood decay fungi typically harbor one or no GH11 genes. *Auricularia delicata* is another exception with three of these endoxylanases. Also unusual among white-rot fungi, none of the *P. gigantea* protein models were assigned to GH95 (Dataset S1). This family includes 1,2-α-fucosidases that hydrolyze the α-Fuc-1,2-Gal linkages in plant xyloglucans.

The *P. gigantea* genome includes representatives for all the peroxidase families reported in basidiomycetes, including LiP, MnP, heme-thiolate peroxidases, and dye-decolorizing type peroxidases (DyP), with the only exception of VP (Text S1; Figures S8–S13). MnP gene expansion is similar to that found in the *C. subvermispora* and *H. annosum* genomes. Beyond class II peroxidases and multicopper oxidases (MCOs), genes encoding auxiliary enzymes involved in ligninolysis were also found such as GMC oxidoreductases (Figures S14–S19; Table S5) and copper radical oxidases (CRO, Figure 4; Table S4). Among the latter group, GLX is coupled to *P. chrysosporium* LiPs via extracellular H_2_O_2_ generation [41]. Consistent with this physiological connection, the *P. gigantea* genome features both GLX- and LiP-encoding genes. GMC genes encoding putative AAO, MOX and glucose oxidase (GOX) may also be involved in H_2_O_2_ production by oxidation of low molecular weight aliphatic and aromatic alcohols. The P2O gene (protein model Phlgi1_130349) lies immediately adjacent to a putative pyranosone dehydratase (Phlgi1_16096) gene. This arrangement is conserved in several wood decay fungi and, in addition to peroxide generation, suggests a route for conversion of glucose to the pyrone antibiotic, cortalcerone [42], [43]. Genes encoding AAD, members of the zinc-type alcohol dehydrogenase superfamily [44], are also abundant in *P. gigantea*. Relatively few genes were predicted to encode CYPs which are generally considered important in the intracellular metabolism of lignin derivatives and related aromatic compounds (Figure S19; Dataset S2).

The repertoire of *P. gigantea* genes contrasts sharply with that of brown-rot polypores, such as *Postia placenta* [45], which lack ligninolytic class II peroxidases, cellobiohydrolases (GH6, GH7), and endoglucanases fused to cellulose binding modules [21], [46] (Figure 4). Unlike *P. gigantea* and other white-rot fungi, brown-rot fungi often lack genes encoding cellobiose dehydrogenase (CDH) and have relatively few lytic polysaccharide monooxygenase genes (LPMOs). Formerly classified as GH61 ‘hydrolases’, the LPMOs are now known to be copper-dependent monooxygenases [18]–[20] capable of enhancing cellulose attack by CDH and cellobiohydrolase (CBH) [47], [48]. With the exception of *Gloeophyllum trabeum*, genes encoding GH74 enzymes have not been found in brown-rot fungi. Two such xyloglucanase genes were identified in *P. gigantea* (Text S1).

In contrast to analysis of genes involved in lignocellulose degradation (Figure 3A), white-rot and brown-rot fungi were not clearly separated by principal component analysis of 14 enzymes involved in lipid metabolism (Figures 3B and S3). However, *P. gigantea* was grouped near *B. adusta* and *P. carnosa.* These associations seem in line with the preferential colonization of softwood substrates by *P. carnosa* [49] and with the efficient degradation of conifer extractives by *B. adusta* culture supernatants [50].The highest contribution to PC1 (26.0% variance) and PC2 (6.8% variance) were aldehyde dehydrogenase and long chain fatty acid CoA ligase, respectively (Figures 3A and S3, Text S1). Also potentially involved in intracellular lipid metabolism, CYP52 and CYP505 clans of cytochrome P450s are associated with degradation of fatty acids and alkanes. Relative to other white-rot fungi, *P. gigantea* had a slightly greater number of CYP52-encoding genes whereas CYP505 gene numbers were similar (Figure 4; Dataset S1; Figures S31, S32; Tables S13–S15).

*P. gigantea* also diverges from other Agaricomycetes with respect to genes encoding proteins that are more distantly connected to lignocellulose degradation, including hydrophobins (Figures S33 and S34; Tables S17–S19), transporters (Table S20) and non laccase MCOs (Figure S20). Detailed analyses are provided for CAZys (Tables S7–S10; Figures S22–S30; Dataset S1), peroxidases (Figures S8–S13), auxiliary proteins, cytochrome P450s (Figures S31–S32; Table S13–S15), potential regulatory genes (Figures S4–S7; Tables S3, S11–S12) and genes involved in secondary metabolite synthesis (Table S16).

### Differential gene expression of *P. gigantea* in response to substrate

Transcript levels were determined in cultures in which the sole carbon source was glucose (Glc), freshly harvested loblolly pine wood (*Pinus taeda*; LP) extracted with acetone (ELP), or freshly harvested but not extracted loblolly pine wood (NELP) (Text S1). GC-MS analysis [51] identified the major extract components as resin acids (46%), triglycerides (13%) and fatty acids (11%) (Text S1; Figure S35; Table S21).

**Figure 5:**
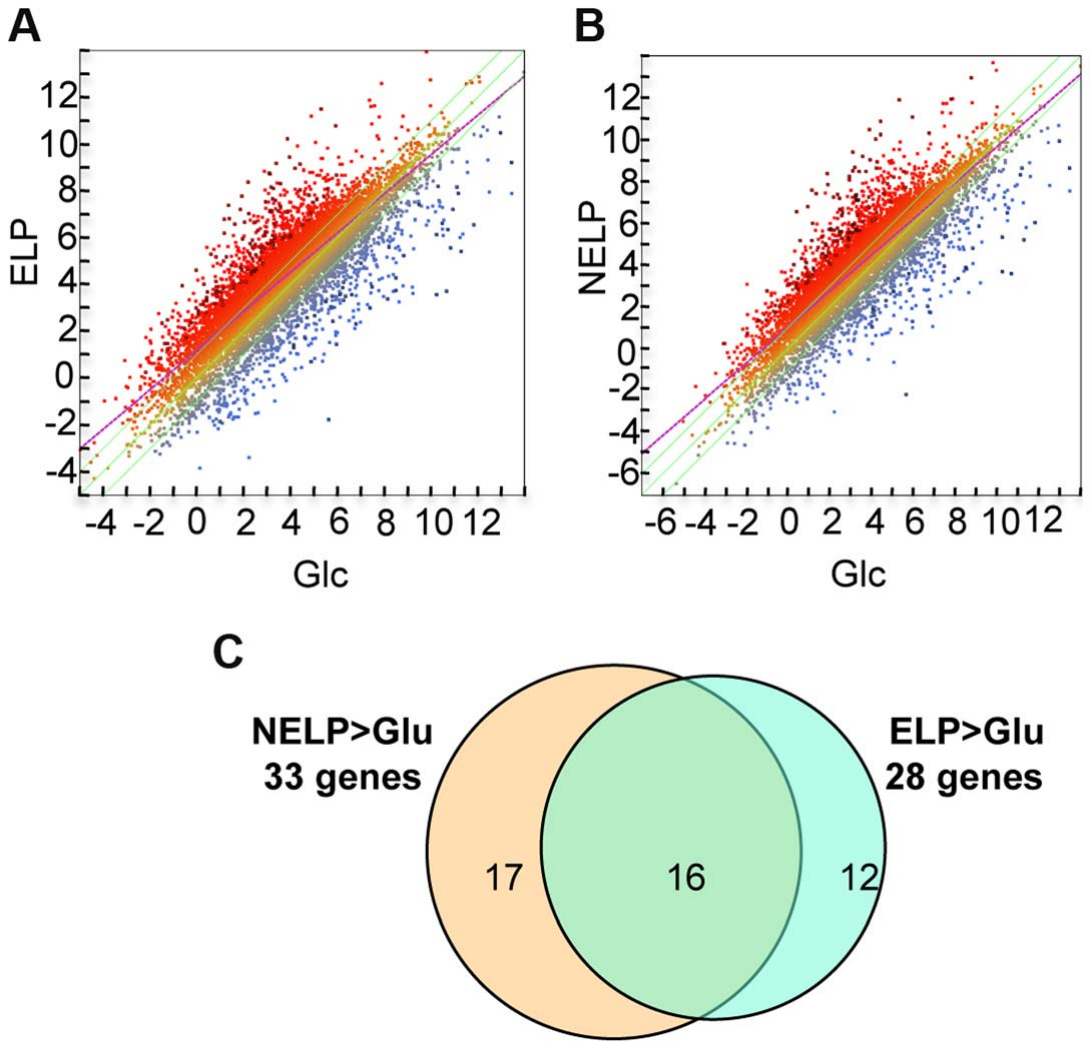
*P. gigantea* transcriptome. Scatterplots show the distribution of RNA-seq RPKM values (log_2_) for 11,376 *P. gigantea* genes when grown on basal salts containing A, acetone-extracted loblolly pine wood (ELP) or B, non-extracted loblolly pine wood (NELP) relative to glucose (Glc). Plot lines define 2-fold borders and best fit regression. Darkened points represent 164 (A) and 145 (B) transcripts accumulating>4-fold at p<0.01. Venn diagram (C) illustrates genes with RPKM signals>10 and upregulated>4-fold in NELP or ELP relative to Glc.

Excluding genes with relatively low transcript levels (RPKM values <10) in LP-containing media, transcripts of 187 genes were increased>2-fold (p<0.05) in NELP or ELP relative to Glc. Of those Glc-derived transcripts with RPKM values>10, 146 genes had higher transcripts in Glc relative to NELP or ELP (Figure 5; Dataset S2).

Mass spectrometry (nanoLC-MS/MS) identified extracellular peptides corresponding to a total of 319 gene products in NELP and ELP cultures (Dataset S2). Most proteins were observed in both NELP and ELP culture filtrates, which contained 294 and 268 proteins, respectively. Approximate protein abundance, expressed as the exponentially modified protein abundance index (emPAI) [52], varied substantially within samples. As expected, gene products with predicted secretion signals and high transcript levels were often detected. Other detected proteins (e.g. MOX model Phlgi1_120749; [53]) may be loosely associated with cell walls and/or secreted via ‘non-classical’ mechanisms ([54]; www.cbs.dtu.dk/services/SecretomeP). Still other peptides correspond to true intracellular proteins released by cell lysis, e.g. ribosomal proteins (Dataset S2).

**Figure 6:**
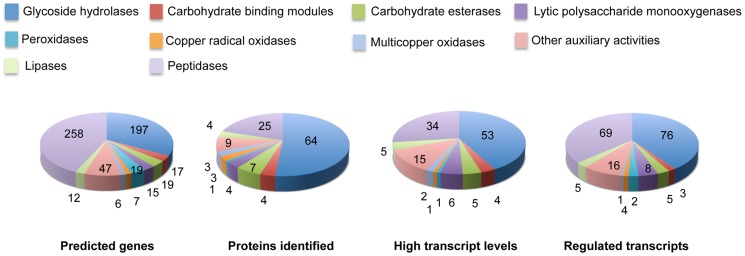
Number and expression of genes likely involved in lignocellulose degradation. The number of genes encoding mass spectrometry-identified proteins was limited to those matching≥2 unique peptides after 5–9 days growth in media containing NELP or ELP. RPKM values>100 for RNA derived from these cultures were arbitrarily selected as the threshold for high transcript levels. Genes designated as ‘regulated’ showed significant accumulation (p<0.05;>2-fold) in NELP or ELP relative to glucose containing media. Methods and complete data are presented in Text S1 and Dataset S2.

Glycoside hydrolase gene expression was heavily influenced by media composition with transcripts corresponding to 76 genes increasing>2-fold in NELP- or ELP-containing media relative to glucose medium (Figure 6). Some of these genes were highly expressed with RPKM values well over 100. For example, transcript and peptide levels matching GH7 cellobiohydrolase (CBH1; model Phlgi1_34136) were among the ten most highly expressed genes (Table 1). Indicative of a complete cellulolytic system, this CBH1 was accompanied by upregulated transcripts and extracellular proteins corresponding to another CBH1 (Phlgi1_13298), a GH6 family member CBH2 (Phlgi1_17701) and GH5_5 β-1,4 endoglucanases (EGs; Phlgi1_86144, Phlgi1_84111), all of which feature a family 1 carbohydrate binding module (CBM1). Also highly expressed were putative β-glucosidases (Phlgi1_127564, Phlgi1_18210) and a GH12 (Phlgi1_34479). Other glycoside hydrolases likely involved in degradation of cell wall hemicelluloses include GH5_7 endomannanases (Phlgi1_97727, Phlgi1_110296), a GH74 xyloglucanase (Phlgi1_98770), a GH27 α-galactosidase (Phlgi1_72848) and a GH10 endoxylanase (Phlgi1_85016).

**Table 1:** Differentially regulated genes in media containing non-extracted loblolly pine wood (NELP), solvent extracted loblolly pine wood (ELP), or glucose (Glc) as sole carbon source.

		emPAI value of filtrates from pine cultures						RNA-seq 5 Day			Transcript ratios (R) & probabilities (P)					
		NELP			ELP			RPKM value			NELP/Glc		NELP/ELP		ELP/Glc	
PRO id	Putative function	5 Day	7 Day	9 Day	5 Day	7 Day	9 Day	NELP	ELP	Glc	Prob	R	Prob	R	Prob	R
Fifteen highly expressed (>100 RPKM) genes exhibiting significant transcript accumulation (p<0.01;>4-fold) in NELP medium relative to Glc medium:																
79150	GH61 LPMO							114	51	2	<0.01	58.6	0.286	2.2	0.021	26.2
74623	Carotenoid ester lipase	0.00	0.39	0.18	0.36	0.00	0.00	313	159	8	<0.01	40.5	0.046	2.0	0.013	20.7
128120	GH15 Glucoamylase	3.06	1.45	0.53	2.35	4.76	4.75	170	47	5	<0.01	32.2	0.010	3.6	0.023	8.9
20514	Cytochrome P450*							368	30	14	<0.01	27.2	<0.01	12.3	0.037	2.2
88425	GH55 β- 1,3 glucanase	0.76	1.48	0.68	0.55	0.81	0.74	149	49	6	<0.01	26.3	0.120	3.0	0.057	8.7
109878	SDH							510	1000	20	<0.01	26.0	0.474	0.5	0.025	51.0
88507	GH18 Chitinase*							409	88	19	<0.01	21.6	<0.01	4.7	0.046	4.6
123837	Zn finger domain*							8223	1745	436	<0.01	18.9	<0.01	4.7	0.057	4.0
36218	Hypothetical	2.48	0.57	0.19	0.85	0.00	0.00	3992	2334	227	<0.01	17.6	0.209	1.7	0.014	10.3
123273	Epoxide hydrolase							139	112	10	<0.01	13.8	0.767	1.2	0.044	11.2
125213	GH61 (LPMO)*							1291	306	109	<0.01	11.8	<0.01	4.2	0.058	2.8
30343	AAD-like OR^1^							308	168	34	<0.01	9.2	0.364	1.8	0.083	5.0
36293	Hypothetical							371	352	46	<0.01	8.1	0.885	1.1	0.012	7.7
105051	Peptidase M							176	51	24	<0.01	7.3	<0.01	3.4	0.065	2.1
107268	Hypothetical							110	69	15	<0.01	7.3	0.035	1.6	0.013	4.6
Thirty highly expressed genes (RPKM>100) exhibiting significant transcript accumulation (p<0.01;>4-fold) in NELP and ELP medium relative to Glc medium:																
34136	GH7 Cellobiohydrolase	16.85	5.77	2.91	35.76	0.82	2.91	3927	2931	40	<0.01	97.7	0.218	1.3	<0.01	72.9
98430	Hexose transporter	0.08	0.06	0.06	0.37	0.00	0.00	2213	1794	63	<0.01	35.4	0.323	1.2	<0.01	28.7
19028	Lipase*	6.86	1.20	0.76	1.79	1.92	1.34	1903	274	22	<0.01	87.7	<0.01	7.0	<0.01	12.6
110296	GH5-7 Mannanase	5.29	24.92	19.23	2.12	0.68	0.87	1448	1243	15	<0.01	97.0	0.597	1.2	<0.01	83.3
101670	Peptidase M35	1.39	0.54	0.28	0.80	0.00	0.00	1232	583	22	<0.01	56.8	0.146	2.1	<0.01	26.9
69030	Aquaporin							1080	518	18	<0.01	58.7	0.011	2.1	<0.01	28.2
17701	GH6 Cellobiohydrolase	3.68	2.17	1.06	2.92	0.21	0.19	699	436	9	<0.01	79.9	0.244	1.6	<0.01	49.8
99876	CDH	5.31	1.37	0.63	5.63	0.12	0.23	602	327	12	<0.01	50.4	0.065	1.8	<0.01	27.4
97727	GH5-7 Mannanase	1.16	0.60	0.24	0.89	0.00	0.00	538	721	16	<0.01	34.5	0.130	0.7	<0.01	46.2
13298	GH7 Cellobiohydrolase	1.00	1.05	0.59	0.64	0.00	0.17	418	296	9	<0.01	45.3	0.215	1.4	<0.01	32.1
98770	GH74 Xyloglucanase	3.53	1.82	0.76	4.36	0.23	0.69	373	446	9	<0.01	43.4	0.307	0.8	<0.01	51.9
227588	GH61 (LPMO)^2^	0.61	0.44	0.09	0.19	0.00	0.00	367	592	11	<0.01	34.5	0.276	0.6	<0.01	55.7
18264	GH61 (LPMO)							344	150	4	<0.01	92.2	0.209	2.3	<0.01	40.2
127564	GH3 β-glucosidase	5.02	5.89	4.87	4.84	0.48	0.33	279	191	8	<0.01	35.6	0.134	1.5	<0.01	24.4
72848	GH27 α-galactosidase	0.70	1.25	0.53	0.74	0.00	0.00	244	232	28	<0.01	8.8	0.850	1.0	<0.01	8.4
86144	GH5-5 Endoglucanase	5.89	1.29	0.61	2.69	0.00	0.00	238	223	4	<0.01	62.2	0.901	1.1	<0.01	58.4
23523	Hypothetical							216	285	50	<0.01	4.3	0.169	0.8	<0.01	5.7
34479	GH12 Endoglucanase							197	114	2	<0.01	87.0	0.210	1.7	<0.01	50.4
85016	GH10 Endoxylanase	34.64	19.63	4.82	18.22	0.75	2.62	175	173	3	<0.01	68.0	0.977	1.0	<0.01	67.0
33910	Transporter							166	284	10	<0.01	16.9	0.057	0.6	<0.01	28.8
18210	GH1 β-glucosidase							164	207	10	<0.01	17.2	0.342	0.8	<0.01	21.7
77281	Hypothetical							158	339	5	<0.01	34.1	0.043	0.5	<0.01	73.5
44970	Hypothetical							157	141	15	<0.01	10.6	0.709	1.1	<0.01	9.5
84111	GH5-5 Endoglucanase	25.88	12.32	3.50	27.46	1.40	1.96	149	146	9	<0.01	15.8	0.971	1.0	<0.01	15.4
27734	Hypothetical							142	200	10	<0.01	13.7	0.045	0.7	<0.01	19.3
Nine highly expressed genes (>100 RPKM) exhibiting significant transcript accumulation (p<0.01;>4-fold) in ELP medium relative to Glc medium:																
424549	Hypothetical							65	115	3	0.014	24.7	0.060	0.6	<0.01	44.1
37108	Hypothetical							78	135	5	0.015	16.6	0.258	0.6	<0.01	28.5
124522	Hypothetical							88	164	12	0.014	7.2	0.113	0.5	<0.01	13.6
85295	Heroxidase DyP							39	145	11	0.050	3.4	0.015	0.3	<0.01	12.8
100874	Hypothetical							295	415	63	0.018	4.7	0.072	0.7	<0.01	6.6
125316	Hypothetical							140	201	35	0.009	3.9	0.060	0.7	<0.01	5.7
128752	Histidine kinase							135	225	44	0.031	3.1	0.060	0.6	<0.01	5.1
63338	PIPkin III							71	137	29	0.036	2.4	0.016	0.5	<0.01	4.7
22173	Hypothetical							68	111	27	0.024	2.5	0.021	0.6	<0.01	4.0

Abbreviations: SDH, short chain dehydrogenase; LPMO, lytic polysaccharide monooxygenase; AAD, aryl alcohol dehydrogenase; CDH, cellobiose dehydrogenase; OR, oxidoreductase; DyP, dye decolorizing peroxidase; PIPkin-III, phosphatidylinositol-3-phosphate 5-kinase.

1 Oxidoreductase is 53% identical to *S. pombe* AAD (Q9P7U2).

2 GH61 LPMO model Phlgi1_227588 is 3′-truncated.

*Protein models cross listed with Tables 2 and 3.

Expression of oxidative enzymes implicated in lignocellulose degradation was also influenced by growth on LP-media (NELP or ELP) relative to Glc-containing media. Transcripts corresponding to five LPMO-encoding genes showed significant regulation (P<0.01) in LP-medium, and three LPMO proteins were detected (Phlgi1_227588, Phlgi1_227560, Phlgi1_37310). An AAD-like oxidoreductase (Phlgi1_30343), possibly involved in the transformation of lignin metabolites, was also upregulated. However, we did not observe high expression of class II peroxidases under the conditions tested (Dataset S2). On the other hand, a DyP (Phlgi1_85295) was significantly upregulated in certain LP-containing media (Table 1). The importance of these peroxidases is further supported by the high protein levels of another DyP, Phlgi1_122124. Specifically, the latter protein showed emPAI values>17 after 5 days growth on LP media and, relative to Glc medium, its transcript ratios were>5-fold higher (p<0.04) (Dataset S2). High DyP gene expression has been observed in white-rot fungi *Trametes versicolor* and *Dichomitus squalens* [21], but no genes for these proteins are present in *P. chrysosporium* and *C. subvermispora* (Figure 4). The *P. gigantea* DyP (Phlgi1_122124) was also abundant in media containing microcrystalline cellulose (Avicel) as the sole carbon source (Dataset S2).

**Figure 7:**
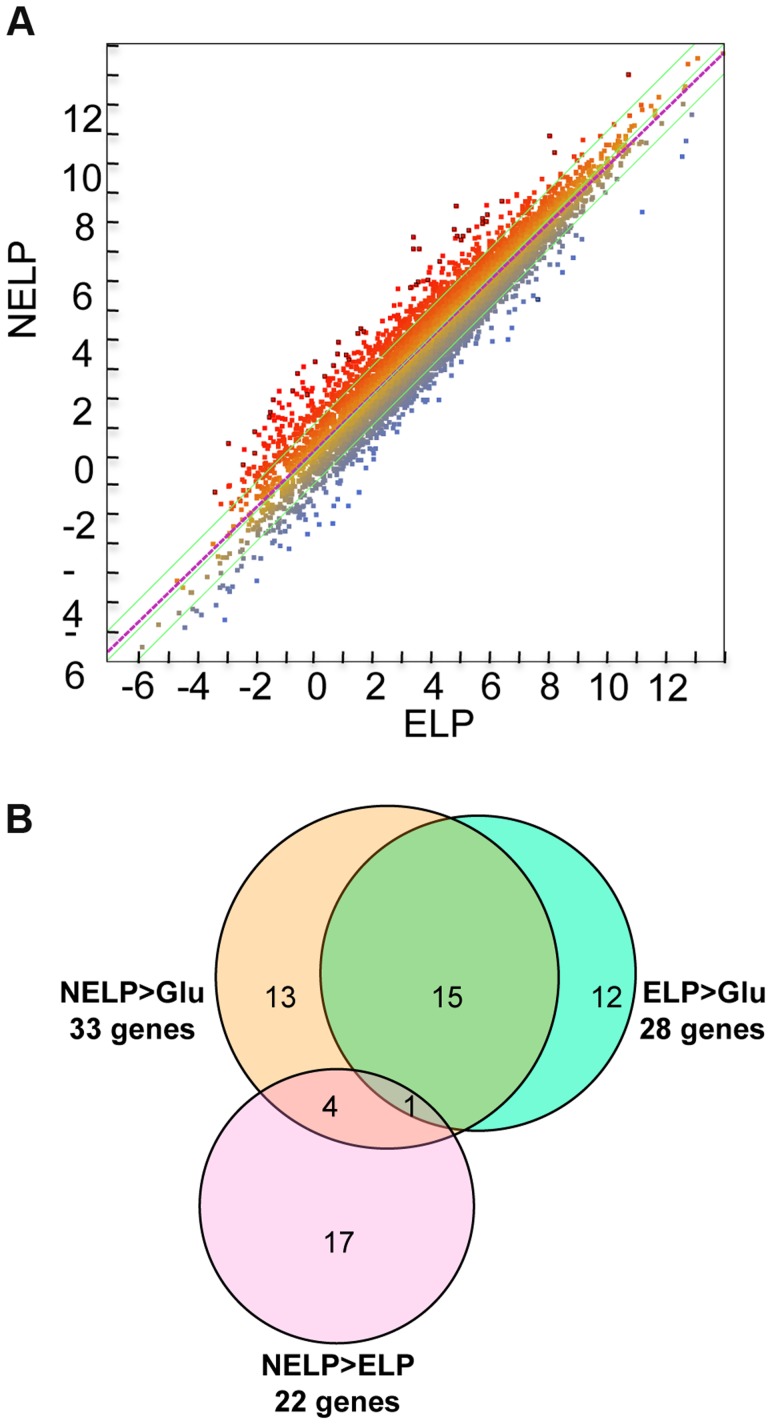
*P. gigantea* transcriptome. Scatterplot (A) shows the distribution of RNA-seq RPKM values (log_2_) for 11,376 *P. gigantea* genes when grown on basal salts containing acetone-extracted loblolly pine wood (ELP) or non-extracted loblolly pine wood (NELP). Lines define 2-fold borders and best fit regression. Darkened points represent 44 transcripts accumulating>4-fold at p<0.01. Venn diagram (B) illustrates genes with RPKM signals>10 and upregulated>4-fold in NELP relative to ELP. Twenty-two genes showed significant transcript accumulation in NELP relative to ELP suggesting potential response to resin and pitch content. Under these stringent thresholds (p<0.01;>4-fold), only one gene, a MCO model Phlgi1_129839, showed significant transcript accumulation in ELP relative to NELP. Additional detail appears in Tables 1-3. Detailed methods and complete data are presented in Text S1 and Dataset S2.

**Figure 8:**
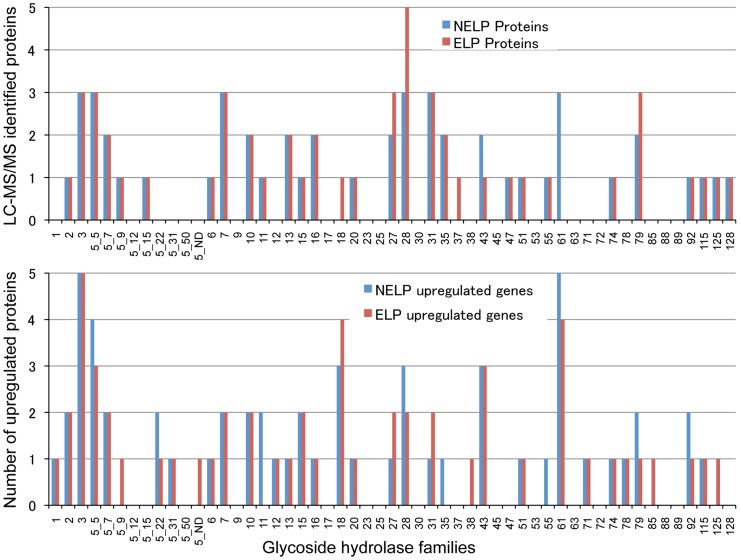
Glycoside hydrolase encoding genes show similar patterns of expression in media containing freshly ground and non-extracted loblolly pine wood (NELP) relative to the same substrate but extracted with acetone (ELP) to remove pitch and resins. Proteins (upper panel) and transcripts (lower panel) were identified by LC-MS/MS and RNA-seq, respectively. Protein identification was limited to those with>2 unique peptides after five days incubation. Transcript upregulation was limited to significant accumulation (p<0.05;>2-fold) on NELP or ELP relative to glucose-containing medium. Secretome and transcriptome experimental details and complete data are presented in Text S1 and Dataset S2.

**Figure 9:**
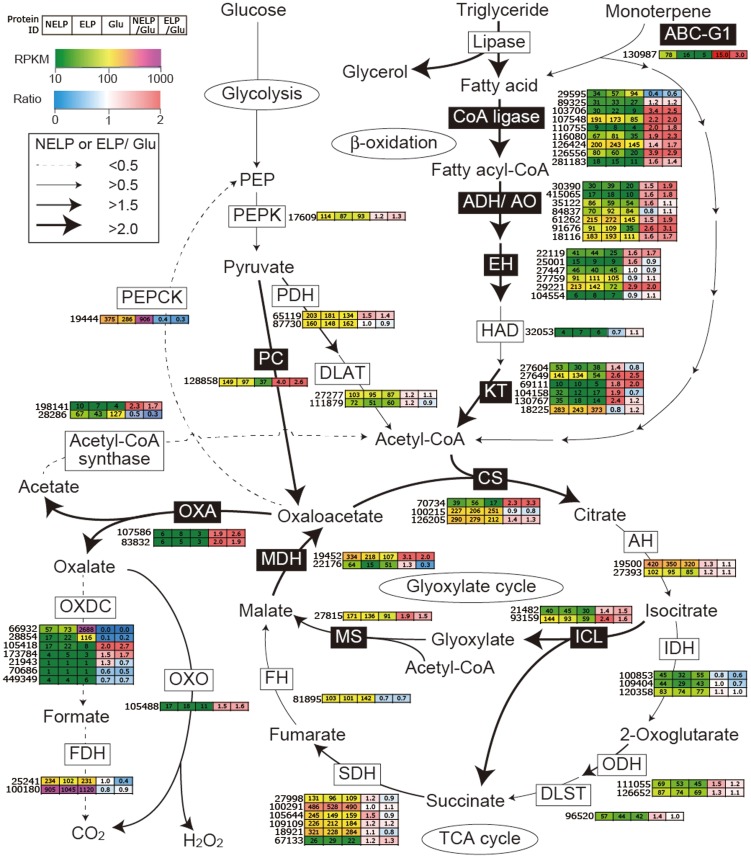
Glyoxalate shunt and proposed relationship to lipid oxidation when *P. gigantea* is cultivated on wood-containing media (ELP or NELP) relative to Glc medium. Enzymes encoded by upregulated genes are black highlighted and associated with thickened arrows. Abbreviations: ABC-G1, ABC transporter associated with monoterpene tolerance; ADH/AO, Acyl-CoA dehydrogenase/oxidase; AH, Aconitate hydratase; CoA ligase, long fatty acid-CoA ligase; DLAT, Dihydrolipoyllysine-residue acetyltransferase; DLST, Dihydrolipoyllysine-residue succinyltransferase; EH, Enoyl-CoA hydratase; FDH, Formate dehydrogenase; FH, Fumarate hydratase; KT, Ketothiolase (acetyl-CoA C-acyltransferase); HAD, 3-Hydroxyacyl-CoA dehydrogenase; ICL, Isocitrate lyase; IDH, Isocitrate dehydrogenase; MDH, Malate dehydrogenase; MS, Malate synthase; ODH, Oxoglutarate dehydrogenase; OXA, Oxaloacetase; OXDC, Oxalate decarboxylase; OXO, Oxalate oxidase; PC, Pyruvate carboxylase; PDH, Pyruvate dehydrogenase; PEP, Phosphoenolpyruvate; PEPCK, Phosphoenolpyruvate carboxykinase; PEPK, Phosphoenolpyruvate kinase; SDH, succinate dehydrogenase. See Dataset S2 for detailed gene expression data.

To identify enzymes involved in tolerance to and/or degradation of extractives, comparisons were made of gene expression in ground loblolly pine wood that had been extensively extracted with acetone (ELP) versus non-extracted loblolly pine wood (NELP) (Figure 7A). In general, this treatment had little impact on gene expression. For example, glycoside hydrolase transcript and protein patterns showed only minor differences (Figure 8). Nevertheless, transcripts corresponding to 22 genes showed significantly increased levels (>4-fold; p<0.01) in NELP relative to ELP (Figure 7B; Table 2). Of particular interest were genes potentially involved in metabolism of resin acids (e.g. CYPs; [55]), in altering the accessibility of cell wall components (e.g., an endoxylanase), and in regulating gene expression (e.g. proteins containing putative Zn finger domains or HMG-Box transcription factors). Integration of transcript profiles of genes involved in intracellular lipid and oxalate metabolism, together with TCA and glyoxylate cycles, strongly supports a central role for β-oxidation in triglyceride and terpenoid transformation by *P. gigantea* (Figure 9).

**Table 2:** Transcripts accumulating>4-fold in non-extracted loblolly pine wood (NELP) relative to extracted loblolly pine wood (ELP).^1^

			RPKM		Ratio	
PRO ID^3^	Putative function	Comments	NELP	ELP	NELP/ELP	Probability
385265	Hypothetical		174.01	10.84	16.058	0.001
20514*	Hytochrome P450	CYP512B	368.06	29.90	12.310	<0.001
118355	Hypothetical	72 aa; IVS	132.48	10.98	12.068	<0.001
21241	GH11 endo-β-1,4-xylanase		133.85	12.68	10.559	0.006
118300	Hypothetical	84 aa	210.69	28.40	7.419	<0.001
119094	Cytochrome P450	CYP5148A	180.08	31.01	5.808	0.008
128107	Hypothetical		53.61	10.25	5.229	0.001
35298	Hypothetical		204.88	40.46	5.064	<0.001
75047	Hypothetical		178.94	35.38	5.057	<0.001
116265	Hypothetical		61.31	12.38	4.954	0.002
99997	Hypothetical		162.61	33.31	4.882	0.008
19184	Cytochrome P450	CYP5136A	99.22	20.33	4.880	0.002
19877	Hypothetical		57.37	11.83	4.849	0.007
123837*	Zn finger domain protein		8222.76	1745.42	4.711	0.002
340250	HMG-Box transcription factor		295.72	63.02	4.693	<0.001
88507*	GH18 Chitinase	CBM5	409.32	87.55	4.675	0.002
122504	Hypothetical		245.20	55.28	4.436	0.006
125213*	GH61 LPMO^2^		1290.80	305.62	4.223	<0.001
36458	HMG-Box transcription factor		256.76	61.38	4.183	0.001

1 Listing limited to genes with RPKM values>10 and high confidence differential expression (p<0.01). Complete listings for 11,892 genes provided in Dataset S2. Abbreviations: aa, amino acids; CYP, cytochrome P450; IVS, long intervening sequence in gene model; CBM, carbohydrate binding module;

2 Truncated gene model predicts incomplete protein (117aa).

3 Nineteen of 22 accumulating in NELP relative to ELP as illustrated in Figure 4B. Three additional upregulated genes were associated with LC-MS/MS-detected proteins and listed in Table 3. Proteins with asterisks are also listed in Table 2.

Relaxing the transcript fold-change threshold (>2-fold; p<0.01) and focusing on mass spectrometry-identified proteins revealed 14 additional genes potentially involved in metabolism and/or tolerance to loblolly pine wood extractives (Table 3).Among these extract-induced genes, lipases Phlgi1_19028 and Phlgi1_36659 likely hydrolyze the significant levels of triglycerides. The substrate specificity of aldehyde dehydrogenases such as Phlgi1_115040 is difficult to assess based on sequence, although several have been implicated in the degradation of pine wood resins by bacteria [56]. Secretome patterns in media containing microcrystalline cellulose (Avicel) as sole carbon source generally supported the importance of the same proteins in the metabolism of pine wood extractives (Table 3, Dataset S2). Specifically, lipases Phlgi1_19028 and Phlgi1_36659 and aldehyde dehydrogenase Phlg1_115040 were more abundant in loblolly pine wood and in Avicel media relative to the same media without extractives. The role of peroxiredoxin (Phlgi1_95619) and glutathione S-transferase (Phlgi1_113065) are less clear, but transformations involving H_2_O_2_ reduction and glutathione conjugation are possible. A single MCO (Phlgi1_129839) and its corresponding transcripts, were observed to be upregulated in ELP relative to NELP. Although lacking the L2 signature common to laccases [57], the MCO4 protein may have iron oxidase activity provided that an imperfectly aligned Glu residue serves in catalysis (Text S1; Figures S20 and S21; Table S6).

**Table 3:** Genes encoding LC-MS/MS detected proteins and exhibiting>2-fold regulation in comparisons of NELP and ELP cultures.^1^

		Pine emPAI values						RNA-seq 5 days			Transcript ratio (R) & Probabilities (Prob)						emPAI values	
		Days in NELP			Days in ELP			RPKM			NELP/Glc		NELP/ELP		ELP/Glc		5 day Avicel	
PRO id	Putative function	5	7	9	5	7	9	NELP	ELP	Glc	Prob	R	Prob	R	Prob	R	+ extr	- extr
19028	Lipase	6.86	1.20	0.76	1.79	1.92	1.34	1903	274	22	0.001	87.7	0.000	7.0	0.007	12.6	2.24	0.80
126044	Cyclophilin	6.77	0.70	0.34	3.63	0.58	0.54	272	56	195	0.637	1.4	0.007	4.9	0.124	0.3	5.89	8.78
22176	Malate dehydrogenase	0.44	0.00	0.07	0.14	1.34	0.27	64	15	51	0.705	1.3	0.003	4.2	0.092	0.3	3.70	7.12
26602	GH17 β-(1-6) endohydrolase	0.00	0.09	0.09	0.00	0.00	0.00	478	124	30	0.119	15.9	0.003	3.8	0.331	4.1	0.00	0.00
95619	Peroxiredoxin	0.26	0.19	0.23	0.00	0.00	0.00	98	26	108	0.773	0.9	0.001	3.8	0.023	0.2	1.35	1.35
113065	Glutathione S-transferase	0.97	0.69	0.05	0.00	0.00	0.00	52	14	45	0.569	1.2	0.000	3.6	0.023	0.3	0.00	0.00
36659	Lipase	0.93	0.00	0.12	0.00	0.00	0.00	285	86	178	0.473	1.6	0.005	3.3	0.292	0.5	0.66	0.22
33454	GH13-CBM20 α-amylase	1.23	1.44	0.67	0.78	1.34	2.16	501	186	168	0.405	3.0	0.005	2.7	0.941	1.1	0.68	0.77
115040	Aldehyde dehydrogenase	0.60	0.00	0.02	0.00	0.00	0.00	101	38	14	0.012	7.4	0.001	2.7	0.057	2.8	1.89	0.51
64365	Ribonuclease T2	0.64	0.25	0.15	0.38	1.06	0.98	80	32	18	0.015	4.5	0.009	2.5	0.107	1.8	0.69	2.31
70525	ATP synthase	0.54	0.12	0.04	0.00	0.00	0.00	22	10	29	0.246	0.8	0.004	2.2	0.020	0.3	0.91	1.37
36220	Glycoprotein	0.17	0.17	0.04	0.12	0.38	0.23	106	48	365	0.204	0.3	0.009	2.2	0.071	0.1	0.00	0.00
19596	Uncharacterized protein	0.97	0.69	0.40	0.45	0.00	0.00	1937	900	2056	0.807	0.9	0.002	2.2	0.035	0.4	0.00	0.00
129839	Multicopper oxidase (MCO4)	1.45	1.03	0.25	2.61	8.79	6.10	40	209	180	0.091	0.2	0.010	0.2	0.852	1.2	0.50	1.02

1 Listing limited to genes with RPKM values>10 and, in comparisons of NELP and ELP cultures, with high confidence of differential expression (p<0.01). Transcript values for cultures grown on microcrystalline cellulose (Avicel) as sole carbon source unavailable. The composition of loblolly pine wood extract (extr) is listed in Text S1. Complete listings for 11,892 genes provided in Dataset S2.

## Discussion

The distinctive repertoire and regulation of *P. gigantea* genes underlie a unique and efficient system for degrading all components of conifer sapwood. Transcriptome and proteome analyses demonstrate an active system of hydrolases and LPMOs involved in the complete deconstruction of cellulose and hemicellulose. The overall enzymatic strategy is therefore similar to most cellulolytic microbes, but unlike closely related brown-rot decay Agaricomycetes such as *P. placenta*.

With regard to ligninolysis, key genes were identified including LiPs, MnPs, CROs and GMC oxidoreductases. As in *P. chrysosporium*, the presence of both LiP- and GLX-encoding genes is consistent with a close physiological connection involving peroxide generation [41]. We also annotated non-class II peroxidases HTPs and DyPs some of which have been implicated in metabolism of lignin derivatives [58], [59]. The distribution and expression of DyP-encoding genes are notable; with no genes present in *P. chrysosporium* and *C. subvermispora* but several highly expressed genes in *T. versicolor, D. squalens* [21] and *P. gigantea* (Table 2). Physiological roles of DyP are likely diverse, but oxidation of lignin-related aromatic compounds has been demonstrated [59].

In addition to lignin, oxidative mechanisms likely play a central role in *P. gigantea* cellulose attack. Of 15 LPMO-encoding genes, transcripts of six genes were regulated (>2-fold; p<0.01) and peptides corresponding to three were unambiguously identified in NELP- or ELP-containing media. Our inability to detect any LPMO proteins in Avicel media (Dataset S2) suggests induction by substrates other than cellulose [60]. Beyond this, the CDH gene was highly expressed (transcripts and protein) in LP media. The observed coordinate expression of CDH and LPMO may reflect oxidative ‘boosting’ as recently demonstrated [19], [20], [47], [61]. However, we did not detect elevated transcripts or peptides corresponding to the two *P. gigantea* aldose 1-epimerase genes even though these were previously observed in culture filtrates of various white-rot fungi [21], [62], including *Bjerkandera adusta, Ganoderma* sp, and *Phlebia brevispora* [17]. Thus, it seems unlikely that enzymatic conversion of oligosaccharides to their β-anomers is necessary for efficient CDH catalysis.

Softwood hemicellulose composition typically includes 15-20% galactoglucomannan while hardwoods contain 15–30% glucuronoxylan [40]. Consistent with an adaption to conifer hemicellulose, GH5_7 β-mannanases were highly expressed in both NELP and ELP cultures, together with a GH27 α-galactosidase (Table 1). GH11 endoxylanase and CE carbohydrate esterase peptides were also detected in the pine wood-containing media, but not in Avicel cultures (Dataset S2). In aggregate, these results demonstrate *P. gigantea* adaptation to conifer hemicellulose degradation.

*P. gigantea*'s gene expression patterns reveal multiple strategies for overcoming the challenging composition of resinous sapwood. Tolerance to monoterpenes may be mediated in part by a putative ABC efflux transporter (Phlbi1_130987, Figure 9). Of the 51 ABC proteins of *P. gigantea*, this protein is most closely related to the *GcABC-G1* gene of the ascomycete *Grosmannia clavigera*, a pathogen of *Pinus contorta* [63]. The *GcABC-G1* gene is upregulated in response to various terpenes and appears to be a key element against the host defenses. Consistent with a similar function, our analysis showed the *P. gigantea* homolog to be upregulated>4.9-fold (p = 0.02) in NELP relative to ELP media (Dataset S2). Other transcripts accumulating in NELP-derived mycelia included three CYPs (Table 2) potentially involved in the hydroxylation of diterpenoids and related resin acids [55]. Differential regulation also implicates glutathione S-transferase, aldehyde dehydrogenase and peroxiredoxin in the transformation and detoxification of extractives (Table 2). Three putative transcription regulators were similarly regulated (Table 3). Aldehyde dehydrogenase- and AAD-encoding genes, some of which are upregulated in *P. gigantea* LP cultures relative to Glc cultures (Tables 1), are induced by aromatic compounds in *P. chrysosporium* [64], [65].

Predicted to degrade triglycerides, a total of nine lipase-encoding genes were identified in the *P. gigantea* genome and four of these were upregulated>2-fold (p<0.01) in LP media compared to Glc medium (Dataset S2). Two lipases displayed similar patterns of transcript and protein upregulation on NELP relative to ELP (Table 3), and the pine wood extractive also induced accumulation of these lipases in Avicel media (Table 3). Further metabolism of triglycerides is uncertain, although a putative glycerol uptake facilitator (Phlbi1_99331) was highly expressed (RPKM value of 2532) and significantly (p<0.02) upregulated (2.1-fold) in NELP compared to ELP (Dataset S2). Fatty acids activation and β-oxidation can be inferred by the expression of fatty acid CoA ligase (Phlgi1_107548, Phlgi1_126556, Phlgi1_89325), β-ketothiolase (Phlgi1_27649, Phlgi1_130767), and fatty acid desaturase (Phlgi1_100083, Phlgi1_115799). Upregulation of a mitochondrial malate dehydrogenase (Phlgi1_22176, Table 3), together with relatively high transcript levels of other TCA cycle components (citrate synthases Phlgi1_126205, Phlgi1_100215; 2-oxoglutarate dehydrogenase, Phlgi1_126652) may complete fatty acid oxidation. In this connection, we also observed high expression of isocitrate lyase (Phlgi1_21482, Phlgi1_93159) and malate synthase (Phlgi1_27815), which partially explain oxalate accumulation [66] and strongly support an active glyoxylate shunt [45], [67] (Figure 9). Upregulation of glycoside hydrolases, transcription factors, cyclophilins, ATP synthase and ribonuclease may also reflect broad shifts in metabolism or reduced accessibility of the unextracted substrate (Tables 2 and 3).

Beyond genetic regulation, certain constitutively expressed genes are also likely involved in the degradation of all plant cell wall components, including complex resins and triglycerides. For example MOX (Phlgi1_120749) is among the most abundant transcripts in both NELP and ELP (Dataset S2), suggesting an important role in H_2_O_2_ production associated with lignin demethylation [53]. Extracellular peroxide generation is key to peroxidase activity, and MOX fulfills this role along with CRO, AAO, and P2O. Along these lines, we also observed high extracellular protein levels of DyP (Phlgi1_122124) under all culture conditions.

Most problematic, many *P. gigantea* genes and proteins exhibited little or no homology to NCBI NR or Swiss-Prot entries. Some of these ‘hypothetical’ or ‘uncharacterized’ proteins are undoubtedly important, particularly those that are highly expressed, regulated and/or secreted. For example, of 92 genes upregulated (>2-fold; p<0.01) in NELP relative to ELP, 51 were designated as hypothetical (Table 2; Dataset S2). Three of these featured predicted secretion signals and peptides were detected in one case. In the absence of biochemical characterization and/or genetic evidence, assigning function to these genes represents a major undertaking. Nevertheless, high throughput transcript and secretome profiling substantially filtered the number of potential targets from a genome-wide estimate of 4744 ‘hypothetical’ genes to the more manageable numbers reported here. More broadly, the results advance understanding of the early and exclusive colonization of coniferous wood by *P. gigantea* and also provide a framework for developing effective wood protection strategies, improving biocontrol agents and identifying useful enzymes [6], [9], [10].

## Methods

### Wood colonization assays

Wood wafers (1 cm by 1 cm by 2 mm) were cut from the sapwood of aspen (*Populus tremuloides*), pine (*P. taeda*) and spruce (*Picea glauca*) and sterilized by autoclaving. Following inoculation by contact with mycelium growing on malt extract agar (15 g malt extract [Difco, Detroit, MI] and 15 g agar per liter of water) in Petri dishes, colonized wafers were harvested 30, 60 and 90 days. Noninoculated wood wafers placed on the same media in Petri dishes served as controls. Wafers were removed 30, 60 or 90 days later, weighed and percent weight loss was determined. Additional wafers were removed at the same time period, immediately frozen to −20°C and prepared for scanning electron microscopy as previously described [68].

### Sequencing and annotation

The genome was sequenced using Illumina and annotated using the JGI Annotation Pipeline [69]. Assembly and annotations are available from JGI portal MycoCosm [38] and deposited to DDBJ/EMBL/GenBank under accession AZAG00000000. The version described in this paper is version AZAG01000000. The completeness of the *P. gigantea* genome was assessed by finding 99.1% of CEGMA proteins conserved across sequenced genomes of eukaryotes [70](Text S1; Tables S1, S2).

### RNA-seq

Mycelium was derived from triplicate cultures of 250 ml basal salts containing: i. 1.25 g freshly-harvested, ground (1mm mesh) loblolly pine wood that had been ‘spiked’ with acetone and thoroughly dried (NELP); or ii. the same material following extended acetone extraction in a Soxhlet apparatus and drying (ELP). The composition of the extract (Text S1) was determined by GC-MS [51]. Duplicate cultures of basal salts medium with glucose as sole carbon source served as a reference. After 5 days incubation, total RNA was purified from frozen mycelium as described [22], [71]. Multiplexed libraries were constructed and sequenced on an Illumina HiSeq2000. DNAStar Inc (Madison, WI) modules SeqNGen and Qseq were used for mapping reads and statistical analysis. Transcriptome data was deposited to the NCBI Gene Expression Omnibus (GEO) database and assigned accession GSE53112 (Reviewer access via http://www.ncbi.nlm.nih.gov/geo/query/acc.cgi?token=ilovmswixtajjez&acc=GSE53112). Experimental details are provided in Text S1 and all transcriptome analyses are summarized in Dataset S2.

### Secretome analysis

With minor modification, NanoLC-MS/MS analysis identified extracellular proteins in culture filtrates as described [22], [72]. For each of the two woody substrates (e.g NELP and ELP), cultures were harvested after 5, 7 and 9 days. Mass spectrometric protein identifications were accepted if they could be established at greater than 95.0% probability within 0.9% False Discovery Rate and contained at least two identified peptides. Protein probabilities were assigned by the Protein Prophet algorithm [73]. To verify the effects of pine wood extractives in a well-defined substrate, media containing microcrystalline cellulose (Avicel) were also employed [22], [45], [74]. Filtrates from these cultures, with or without addition of loblolly pine wood acetone extract, were collected after 5 days and analyzed. Approximate protein abundance in each of the cultures was expressed as the number of unique peptide and the exponentially modified protein abundance index (emPAI) value [52] (See Text S1 for detailed methods).

## Supporting Information

Figure S1 Vista dot plot illustrating syntenic relationship between 12 longest scaffolds of *P. gigantea* and *P. chrysosporium*.
(EPS)

Figure S2 Top 20 families of contributing to PC1 and PC2 values in Figure 3A. The x-axis designates each enzyme family and y-axis indicates the squared rotation values for PC1 and PC2. As shown in Figure 2, the PC2 value mainly separated the white- and brown-rot fungi.
(EPS)

Figure S3 Ten genes encoding enzymes potentially involved in lipid metabolism contributing to PC1 and PC2 values in Figure 3B. The x-axis designates each enzyme family and y-axis indicates the squared rotation values for PC1 and PC2.
(EPS)

Figure S4 Phylogenetic analysis of opsin genes from *P. gigantea* (Phlgi), *C. subvermispora* (Cersu), *P. placenta* (Pospl), *A. nidulans* (AN), *Sordaria macrospora* (SM) and *Neurospora crassa* (NCU). The evolutionary history was inferred using the Minimum Evolution method [78]. The bootstrap consensus tree inferred from 500 replicates (MEGA4) is taken to represent the evolutionary history of the taxa analyzed (MEGA4). Branches corresponding to partitions reproduced in less than 50% bootstrap replicates are collapsed. The percentage of replicate trees in which the associated taxa clustered together in the bootstrap test (500 replicates) are shown next to the branches (MEGA4). The tree is drawn to scale, with branch lengths in the same units as those of the evolutionary distances used to infer the phylogenetic tree. The evolutionary distances were computed using the Poisson correction method [79] and are in the units of the number of amino acid substitutions per site. The ME tree was searched using the Close-Neighbor-Interchange (CNI) algorithm [4] at a search level of 1. The Neighbor-joining algorithm [80] was used to generate the initial tree. All positions containing gaps and missing data were eliminated from the dataset (Complete deletion option). There were a total of 183 positions in the final dataset. Phylogenetic analyses were conducted in MEGA4 [81].
(EPS)

Figure S5 Phylogenetic analysis of putative photoreceptors of *Ceriporiopsis subvermispora* (Cersu), *Phlebiopsis gigantea* (Phlgi), *Postia placenta* (Pospl), *Cryptococcus neoformans* (CN), *Laccaria bicolor* (LB), *Phycomyces blakesleeanus* (PB), *Neurospora crassa* (NCU), *Aspergillus nidulans* (ANIDU) and *Trichoderma reesei* (TR). Along with the species, the name is given of the respective protein (if known) and the GenBank accession number or protein ID in JGI genome databases.
(EPS)

Figure S6 Alignment of the homologous region comprising the PAS/LOV domain (NCRFLQ) in photoreceptor orthologues. LOV signatures are highlighted.
(EPS)

Figure S7 Genomic locus comprising the cluster of response regulator genes.
(EPS)

Figure S8 Homology models for the molecular structures of class II heme peroxidases from the *P. gigantea* genome. Ligninolytic peroxidases, including LiP models - **A**) 150531 peroxidase, **B**) 121662 peroxidase and **C**) 30372 peroxidase - harboring an exposed tryptophan potentially involved in oxidation of high redox-potential substrates, and **MnP** models - **D**) 75566 peroxidase, **E**) 75572 peroxidase, **F**) 115591 peroxidase, **G**) 115592 peroxidase and **H**) 117668 peroxidase - harboring a putative Mn^2+^ oxidation site (formed by two glutamates and one aspartate); and **I**) manually curated GP (32509). Note that an alanine and an asparagine residues in the LiP models occupy the position of the catalytic glutamate and aspartate involved in Mn^2+^ oxidation by MnP, and a serine residue in the MnP models occupies the position of the putative catalytic tryptophan characterizing LiP. The amino acid numbering refers to putative mature sequences, after manual processing of their peptide sequences.
(EPS)

Figure S9 Dendrogram showing evolutionary relationships among 478 basidiomycete heme peroxidases, including structural-functional classification based on Ruiz-Dueñas et al. (2) (GeneBank and JGI references in parentheses and *P. gigantea* genome references on yellow background). Amino-acid sequence comparisons as Poisson distances and clustering based on UPGMA and "pairwise deletion" option of MEGA5 [81]. Compressed sub-trees are shown to facilitate the *P. gigantea* peroxidases analysis. Numbers on branches represent bootstrap values (based on 1000 replications) supporting that branch; only the values ≥50% are presented. **Fungal abbreviations:** AGABI, *Agaricus bisporus*; AURDE, *Auricularia delicata*; BJEAD, *Bjerkandera adusta*; CERSU, *Ceriporiopsis subvermispora*-B; COPCI, *Coprinopsis cinerea*; DACSP, *Dacryopinax* sp.; DICSQ, *Dichomitus squalens* v1.0; FOMME, *Fomitiporia mediterranea* v1.0; FOMPI, *Fomitopsis pinicola* SS1 v1.0; IZU, basidiomycete IZU-154; LACBI, *Laccaria bicolor v2.0*; LENED, *Lentinula edodes*; PHACH, *Phanerochaete chrysosporium*; PHASO, *Phanerochaete sordida*; PHLBR, *Phlebia brevispora* HHB-7030 SS6 v1.0; PHLGI, *Phlebiopsis gigantea*; POSPL, *Postia placenta*; PUNST, *Punctularia strigosozonata* v1.0; SERLA, *Serpula lacrymans*; SPOSP, *Spongipellis* sp.; STEHI, *Stereum hirsutum* FP-91666 SS1 v1.0; TRACE, *Trametopsis cervina*; TREME, *Tremella mesenterica*; USTMA, *Ustilago maydis*. Most of the sequences included in the dendrogram were obtained from the analysis of fungal genome sequences. The genome version from which the peroxidase sequence was obtained is in some cases indicated as v1.0 and v2.0. **Peroxidase abbreviations**: i) GP, generic peroxidase; ii) MnP-short, MnP-long and MnP-extralong, three different mangenese peroxidase (MnP) subfamilies including a typical Mn(II)-oxidation site, formed by two glutamates and one aspartate residues, and differing in the length of their C-terminal tails; iii) LiP, lignin peroxidase harboring an exposed tryptophan residue located at the same position described for the catalytic Trp171 of *P. chrysosporium* LiP; iv) VP, versatile peroxidase including a Mn(II)-oxidation site like in MnP, and a catalytic tryptophan like in LiP; v) VP-LiP intermediate states, two *Ceriporiopsis subvermispora* peroxidases occupying an intermediate position between typical LiPs and VPs according to their structural and catalytic properties [22]; and vi) MnP-atypical and VP-atypical, MnP and VP lacking one of the three acid residues forming the typical Mn(II)-oxidation site present in MnP and VP (Glu35/36, Glu39/40 and Asp179/175 in *P. chrysosporiun* MnP/*P.eryngii* VP).
(PDF)

Figure S10 **Homology models for the molecular structures of heme-thiolate peroxidases (HTPs) from the *P. gigantea* genome.** A) Phlgi1_131735 peroxidase, B) Phlgi1_18201 peroxidase, C) Phlgi1_19534 peroxidase and D) Phlgi1_104428 peroxidase, including proximal cysteine residue acting as the fifth heme iron ligand and a few more amino acid residues of the active center.
(EPS)

Figure S11 **Homology models for the molecular structures of DyP peroxidases from the *P. gigantea* genome.** A) Phlgi1_122124 peroxidase (old Phlgi1_78526 peroxidase), B) Phlgi1_71660 peroxidase, C) Phlgi1_85295 peroxidase and D) Phlgi1_125681 peroxidase, including amino acid residues located at the proximal and distal sides of the heme active site involved in catalysis.
(EPS)

Figure S12 **Dendrogram focused on class II heme peroxidases (a total of 219) showing evolutionary relationships and structural-functional classification.** A) Short, long and extralong MnPs have a Mn^2+^-oxidation site formed by two glutamic and one aspartic residues, and differ in the length of the C-terminal tail; B) LiPs contain a catalytic tryptophan, with the only exception of TRACE-LiP being the “unique” ligninolytic peroxidase with a catalytic tyrosine (3); C) VPs harbor the catalytic sites described above for both MnPs and LiPs; D) GPs do not contain any of the above two catalytic sites; and E) atypical MnPs and VPs lack one of the three acidic residues forming the Mn^2+^-oxidation site. The analysis is described in **Figure S9**. **Fungal abbreviations**: AGABI, *Agaricus bisporus*; AURDE, *Auricularia delicata*; BJEAD, *Bjerkandera adusta*; BJESP, *Bjerkandera sp*; CERRI, *Ceriporiopsis rivulosa*; CERSU, *Ceriporiopsis subvermispora*-B; CERUN, *Cerrena unicolor*; COPCI, *Coprinopsis cinerea*; COPDI, *Coprinellus disseminatus*; DICSQ, *Dichomitus squalens* v1.0; FOMME, *Fomitiporia mediterranea* v1.0; FOMPI, *Fomitopsis pinicola* SS1 v1.0; GANAP, *Ganoderma applanatum*; GANAU, *Ganoderma australe*; GANFO, *Ganoderma formosanum*; GANLU, *Ganoderma lucidum*; GANSP, *Ganoderma* sp.; HETAN, *Heterobasidion annosum* v2.0; IZU, basidiomycete IZU-154; LACBI, *Laccaria bicolor v2.0*; LENED, *Lentinula edodes*; PHACH, *Phanerochaete chrysosporium*; PHASO, *Phanerochaete sordida*; PHLBR, *Phlebia brevispora* HHB-7030 SS6 v1.0; PHLGI, *Phlebiopsis gigantea*; PHLRA, *Phlebia radiata*; PLEER, *Pleurotus eryngii*; PLEOS, *Pleurotus ostreatus*; PLEPU, *Pleurotus pulmonarius*; PLESA, *Pleurotus sapidus*; POSPL, *Postia placenta*; PUNST, *Punctularia strigosozonata* v1.0; SPOSP, *Spongipellis* sp.; STEHI, *Stereum hirsutum* FP-91666 SS1 v1.0; TAICA, *Taiwanofungus camphoratus*; TRACE, *Trametopsis cervina*; TRAVE, *Trametes versicolor*; WOLCO, *Wolfiporia cocos* MD-104 SS10 v1.0. GeneBank and JGI references are shown in parentheses and *P. gigantea* genome references on yellow background.
(PDF)

Figure S13 **Dendrogram focused on DyP peroxidases (a total of 64) showing evolutionary relationships.** The analysis is described in Figure S7. **Fungal abbreviations**: AURAU, *Auricularia auricula-judae*; AURDE, *Auricularia delicata*; BJEAD, *Bjerkandera adusta*; COPCI, *Coprinopsis cinerea*; DICSQ, *Dichomitus squalens* v1.0; FOMME, *Fomitiporia mediterranea* v1.0; GANLU, *Ganoderma lucidum*; GANSP, *Ganoderma* sp.; HETAN, *Heterobasidion annosum* v2.0; LACBI, *Laccaria bicolor v2.0*; MARSC, *Marasmius scorodonius*; MELLA, *Melampsora laricis-populina v1.0*; PHLBR, *Phlebia brevispora* HHB-7030 SS6 v1.0; PHLGI, *Phlebiopsis gigantea*; PLEOS, *Pleurotus ostreatus*; POSPL, *Postia placenta*; PUNST, *Punctularia strigosozonata* v1.0; STEHI, *Stereum hirsutum* FP-91666 SS1 v1.0; TERAL, *Termitomyces albuminosus*; TRAVE, *Trametes versicolor*. GenBank and JGI references are shown in parentheses and *P. gigantea* genome references on yellow background.
(PDF)

Figure S14 **Multiple alignments of aryl alcohol oxidases (AAO) sequences from *P. gigantea* (Phlgi128071, Phlgi121514) and *P. eryngii* (AAOpe) [82].** Highly conserved histidine active site residues [83] are in red. Bottom lines show conserved motifs in GMC family [84]. Obtained using Clustal W [85].The Phlgi128071 and Phlgi121514 models conserve the substrate-binding pocket reported for AAO from *P. eryngii* and the motifs in GMC family.
(EPS)

Figure S15 **Multiple alignments of methanol oxidase (MOX) sequences from *P. gigantea* (Phlgi120749, Phlgi108516 and Phlgi72751) and *G. trabeum* (ABI14440.1)[53].** Highly conserved histidine/asparagine active site residues [84] are in red. Bottom lines show conserved motifs in GMC family [84]. Obtained using Clustal W [85]. The MOX models conserve motifs in GMC family.
(EPS)

Figure S16 **Multiple alignments of cellobiose dehydrogenase (CDH) sequence from *P. gigantea* (model Phlgi99876) and *Gelatoporia subvermispora* (ACF60617).** Highly conserved active site residues are in red [86]. Obtained using Clustal W [85].
(EPS)

Figure S17 **Multiple alignments of pyranose oxidse (POX) sequences from *P. gigantea* (Phlgi130349), *Peniphora sp.* (AAO13382.1) and *Trametes ochracea* (AAP40332.1).** Obtained using Clustal W [85].
(EPS)

Figure S18 **Multiple alignments of glucose oxidase (GOX) sequences from *P. gigantea* (Phlgi128108), *B. fuckeliana* (CDA88590.1) and *C. immitis* (EAS27606) and *A. niger* (AAF59929.2) [1].** Highly conserved histidine active site residues [84] are in red. Bottom lines show conserved motifs in GMC family [84]. Obtained using Clustal W [85].
(EPS)

Figure S19 **Multiple alignments of eight putative aryl alcohol dehydrogenase (AAD) sequences from *P. gigantea* (Phlgi1) and *P. chrysosporium* (AAA61931.1)[36].** Obtained using Clustal W [85].
(EPS)

Figure S20 **Phylogenetic tree of multicopper oxidases from *Acremonium* sp. (Acr), *Aspergillus nidulans* (Ani), *Cryptococcus neoformans* (Cne), *Coprinopsis cinerea* (Cci), *Pleurotus ostreatus* (Pos), *Phanerochaete carnosa* (Pca), *Phanerochaete chrysosporium* (Pch), *Phanerochaete flavido-alba* (Pfa), *Phlebiopsis gigantea* (Pgi), *Postia placenta* (Ppl), *Saccharomyces cerevisiae* (Sce), *Schizophyllum commune* (Sco), *Serpula lacrymans* (Sla), *Sporobolomyces roseus* (Sro), *Tremella mesenterica* (Tme), and *Ustilago maydis* (Uma). Alignments were produced in program ClustalW, manually adjusted in Genedoc and computed in MEGA4.0 for phylogenetic tree production (neighbour-joining, bootstrap values 500).** ID numbers refer to protein models in the JGI MycoCosm (http://genome.jgi-psf.org/programs/fungi/index.jsf), other accession numbers to the NCBI database (http://www.ncbi.nlm.nih.gov/), specific names to proteins specified with accession numbers in Hoegger et al. [29] and Lettera et al. [87]. Blue coloring marks enzymes with laccase activity experimentally shown, light brown enzymes with proven ferroxidase activity, purple enzymes with ascorbate activity, olive enzymes acting in fungal pigment synthesis, and two colors dual enzymatic activities with the left color marking the respective major performance ([88], [89] and references in the review of Kües and Rühl 2011 [90]). Proteins from *P. gigantea* are highlighted in red.
(EPS)

Figure S21 **Sequence alignment for four regions of *S. cerevisiae* ferroxidase Fet3 with corresponding regions of enzymes of *P. gigantea* and *Phanerochaete* species. Marked in yellow are residues that in Fet3 of *S. cerevisiae* are critical for Fe^2+^ binding and the electron-transfer pathway (for references see [90]).** Three groups of enzymes become obvious: i) the Fet3-type ferroxidases; ii) within the cluster of ferroxidases/laccases one subgroup that is more similar to the Fet3-type ferroxidases to which the ferroxidase Mco1 of *P. chrysosporium* belongs to; and iii) one more distinct subgroup that misses three amino acids in the second region of importance for binding pocket formation and to which *P. favido-alba bona fide* laccase PfaL belongs.
(EPS)

Figure S22 **Phylogenetic analysis and subfamily assignments of GH5 protein models of *P. gigantea* (Phlgi), *H. annosum* (Hetan) and *Stereum hirsutum* (stehi).**
(EPS)

Figure S23 **Phylogenetic analysis of LPMO proteins of *P. gigantea*, *C. subvermispora*, and *P. Chrysosporium.***
(EPS)

Figure S24 **Phylogeny and differential expression of carbohydrate esterase family 1 (CE1) genes. CDS sequences were obtained from each genome database according to assigned protein IDs.** Incomplete CDS sequences (partial fragments) were eliminated from the analysis. For each CE family, a multiple alignment was performed using MegAlign version 10 software. The phylogenetic tree was then constructed from the multiple alignment using Clustal W [85]. Numbers at the nodes represent bootstrap values, based on 1000 replications. Species: Aurde, *Auricularia delicata* SS-5; Conpu, *Coniophora puteana*; CC1G, *Coprinopsis cinerea*; Dicsq, *Dichomitus squalens*; Fomme, *Fomitiporia mediterranea*; Fompi, *Fomitopsis pinicola* FP-58527 SS1; Glotr, *Gloeophyllum trabeum*; Hetan, *Heterobasidion annosum*; Lacbi, *Laccaria bico*lor; Phaca, *Phanerochaete carnosa* HHB-10118; Phach, *Phanerochaete chrysosporium* RP78; Phlgi, *Phlebiopsis gigantea*; Punst, *Punctularia strigosozonata*; Schco, *Schizophyllum commune*; Serla, *Serpula lacrymans* S7.3; Stehi, *Stereum hirsutum* FP-91666 SS1; Trave, *Trametes versicolor*; Wolco, *Wolfiporia cocos* MD-104 SS10. Ecology: W, white rot; B, brown rot; M, mycorrhiza; S, non-wood decay saprotroph. Location of comprised CBM1 was indicated as N or C-terminal. Differential regulation of Phlgi CE transcripts between the cultivations tested in this study were also indicated as up- (U) or down- (D) regulated. Asterisk was accompanied if the p value was <0.05. Possible CE1 gene Phlgi_121418 was excluded as the model was severely truncated (91 amino acid residues).
(PDF)

Figure S25 **Phylogeny and differential expression of CE4 genes. Analysis and abbreviations as in Figure S24.**
(PDF)

Figure S26 **Phylogeny and differential expression of CE8 genes. Analysis and abbreviations as in Figure S24.** Possible CE8 gene Phlgi_132681 was excluded as the model was severely truncated (79 residues).
(PDF)

Figure S27 **Phylogeny and differential expression of CE9 genes. Analysis and abbreviations as in Figure S24.**
(PDF)

Figure S28 **Phylogeny and differential expression of CE12 genes. Analysis and abbreviations as in Figure S24.**
(PDF)

Figure S29 **Phylogeny and differential expression of CE15 genes. Analysis and abbreviations as in Figure S24.**
(PDF)

Figure S30 **Phylogeny and differential expression of CE16 genes. Analysis and abbreviations as in Figure S24.** Possible CE16 gene Phlgi_73119 was excluded as the model was severely truncated (95 residues).
(PDF)

Figure S31 **Phylogenetic tree of the cytochrome P450 proteins (P450ome) in *P. gigantea*.** Tree was constructed using 124 P450 sequences (which were full-length or near full-length) and evolutionary history was inferred using bootstrap Neighbor-Joining method. Phylogenetic analyses were conducted using MEGA4 [81]. The P450 listing in the tree is based on the corresponding protein ID with the CYP name in parenthesis. P450s that belong to a new subfamily are indicated with the abbreviation NS.
(EPS)

Figure S32 **Comparative evolutionary analysis of the P450omes of *P. gigantea* and *Phanerochaete* species (*P. chrysosporium* and *P. carnosa)*.** Clan level comparison was made for this analysis.
(EPS)

Figure S33 **Multiple alignment of representative protein sequences of *P. gigantea* hydrophobins.** Sequences were aligned using MUSCLE alignment tool implemented in Molecular Evolutionary Genetic Analysis software (MEGA 5.0). MUSCLE was chosen because it is computationally more suitable for multiple sequence alignments and gives a better accuracy than the conventional CLUSTAL alignment [91]. The aligned protein sequences were viewed with the Biological sequence alignment editor (Bioedit), windows 95/98/NT/2K/XP. Identical amino acid residues are marked in black, conserved cysteine residues are marked with asterisks.
(EPS)

Figure S34 **The evolutionary history of *P. gigantea* hydrophobins with a selected set of closely related basidiomycetes and *Acremonium alcalophilum*, an ascomycete.** Evolutionary relatedness was inferred using the Neighbor-Joining method [80]. The percentage of replicate trees in which the associated taxa clustered together in the bootstrap test (500 replicates) are shown next to the branches. Branches with blue colour represent hydrophobin sequences from *P. gigantea* and branches without support values are less than 50%. The tree is drawn to scale, with branch lengths in the same units as those of the evolutionary distances used to infer the phylogenetic tree. The evolutionary distances were computed using the Poisson correction method [79], and are in the units of the number of amino acid substitutions per site. The analysis involved 174 amino acid sequences. All ambiguous positions were removed for each sequence pair. There were a total of 155 positions in the final dataset. Evolutionary analyses were conducted in MEGA5 [92]. Fungal species IDs: |Cersu| (*Ceriporiopsis subvermispora*), |Phlgi| (*Phlebiopsis gigantea*), |Phchr|(*Phanerochaete chrysosporium*), |Gansp| (*Ganoderma sp*.), |Phlbr| (*Phlebia brevispora*), |Serla_varsha|(*Serpula lacrymans*), |Wolco| (*Wolfiporia cocos*), |Hetan| (*Heterobasidion annosum*), |Schco| (*Schizophyllum commune*), |Copci| (*Coprinopsis cinerea*), |Lacbi| (*Laccaria bicolor*), |Ustma| (*Ustilago maydis*), |Acral| (*Acremonium alcalophilum)*. The tree is rooted at *Acremonium alcalophilum*, representing class II of hydrophobins.
(EPS)

Figure S35 **Chromatogram of loblolly pine wood acetone extract.**
(EPS)

Table S1 **Genome assembly.**
(DOCX)

Table S2 **Annotation.**
(DOCX)

Table S3 **Transcript profiles of response regulator genes clustered on scaffold.**
(DOCX)

Table S4 **Copper radical oxidases (CROs) of *P. Gigantea.***
(DOCX)

Table S5 **Expression of *P. gigantea* GMC oxidoreductases in solvent extracted lodgepole pine wood (ELP) and non-extracted lodgepole pine wood (NELP).**
(DOCX)

Table S6 **Multicopper oxidases of *P. gigantea.***
(DOCX)

Table S7 **Glycoside hydrolase comparisons of brown-rot (BR) and white-rot (WR) fungi.**
(DOCX)

Table S8 **Carbohydrate esterase comparisons of brown-rot (BR) and white-rot (WR) fungi.**
(DOCX)

Table S9 **Polysaccharide lyase comparisons of brown-rot (BR) and white-rot (WR) fungi.**
(DOCX)

Table S10 **Protein features and regulation of lytic polysaccharide monooxygenases (LPMO) in *P. gigantea.***
(DOCX)

Table S11 **Properties of the transcription factors for which binding sites have been detected in CAZymes that are significantly regulated during growth on ground pine wood.**
(DOCX)

Table S12 **Transcript levels of potential regulators in *P. gigantea* cultures.**
(DOCX)

Table S13 **Overview of the *P. gigantea* P450ome and comparison with *P. chrysosporium and P. carnosa.***
(DOCX)

Table S14 **Clan-, family-, and subfamily- level classification of the P450ome of *P. gigantea* and comparison with *P. chrysosporium* and *P. carnosa.***
(DOCX)

Table S15 **Expression profile of *P. gigantea* P450ome.**
(DOCX)

Table S16 **Products of annotated putative secondary metabolite genes in the *P. gigantea* genome.**
(DOCX)

Table S17 ***P. gigantea* hydrophobin models.**
(DOCX)

Table S18 **Fungal species and hydrophobin gene number used for phylogenomics analysis.**
(DOCX)

Table S19 **RNAseq analysis of *P. gigantea* hydrophobins.**
(DOCX)

Table S20 ***P. gigantea* ABC models.**
(DOCX)

Table S21 **Chemical composition of lipids from Loblolly pine wood (*Pinus taeda*).**
(DOCX)

Text S1 **Detailed description of methods and annotated gene families.**
(DOCX)

Dataset S1 **Number and distribution of genes used for PCA.**
(XLSX)

Dataset S2 **Complete listing of *P. gigantea* protein models and expression data.**
(XLSX)
