# Supplementary figures and images for: Analysis of the Phlebiopsis gigantea Genome, Transcriptome and Secretome Provides Insight into Its Pioneer Colonization Strategies of Wood
Source: PLoS Genet. 2014 Dec 4;10(12):e1004759. doi: 10.1371/journal.pgen.1004759 (PMC4256170; doi:10.1371/journal.pgen.1004759)

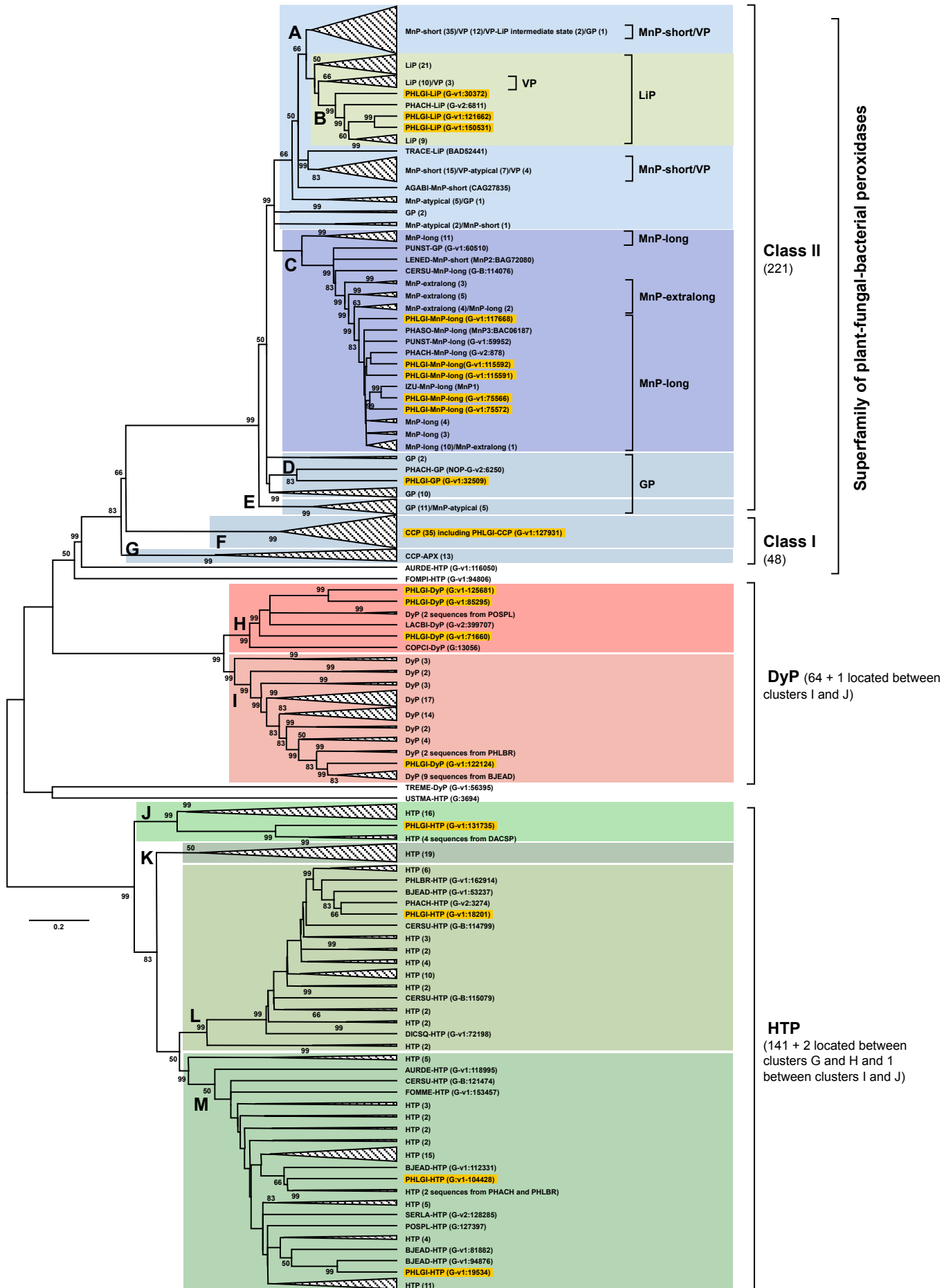

Supplement: Figure S9 — Dendrogram showing evolutionary relationships among 478 basidiomycete heme peroxidases, including structural-functional classification based on Ruiz-Dueñas et al. (2) (GeneBank and JGI references in parentheses and P. gigantea genome references on yellow background). Amino-acid sequence comparisons as Poisson distances and clustering based on UPGMA and "pairwise deletion" option of MEGA5 [81]. Compressed sub-trees are shown to facilitate the P. gigantea peroxidases analysis. Numbers on branches represent bootstrap values (based on 1000 replications) supporting that branch; only the values ≥50% are presented. Fungal abbreviations: AGABI, Agaricus bisporus; AURDE, Auricularia delicata; BJEAD, Bjerkandera adusta; CERSU, Ceriporiopsis subvermispora-B; COPCI, Coprinopsis cinerea; DACSP, Dacryopinax sp.; DICSQ, Dichomitus squalens v1.0; FOMME, Fomitiporia mediterranea v1.0; FOMPI, Fomitopsis pinicola SS1 v1.0; IZU, basidiomycete IZU-154; LACBI, Laccaria bicolor v2.0; LENED, Lentinula edodes; PHACH, Phanerochaete chrysosporium; PHASO, Phanerochaete sordida; PHLBR, Phlebia brevispora HHB-7030 SS6 v1.0; PHLGI, Phlebiopsis gigantea; POSPL, Postia placenta; PUNST, Punctularia strigosozonata v1.0; SERLA, Serpula lacrymans; SPOSP, Spongipellis sp.; STEHI, Stereum hirsutum FP-91666 SS1 v1.0; TRACE, Trametopsis cervina; TREME, Tremella mesenterica; USTMA, Ustilago maydis. Most of the sequences included in the dendrogram were obtained from the analysis of fungal genome sequences. The genome version from which the peroxidase sequence was obtained is in some cases indicated as v1.0 and v2.0. Peroxidase abbreviations: i) GP, generic peroxidase; ii) MnP-short, MnP-long and MnP-extralong, three different mangenese peroxidase (MnP) subfamilies including a typical Mn(II)-oxidation site, formed by two glutamates and one aspartate residues, and differing in the length of their C-terminal tails; iii) LiP, lignin peroxidase harboring an exposed tryptophan residue located at the same position [file pgen.1004759.s009.pdf]

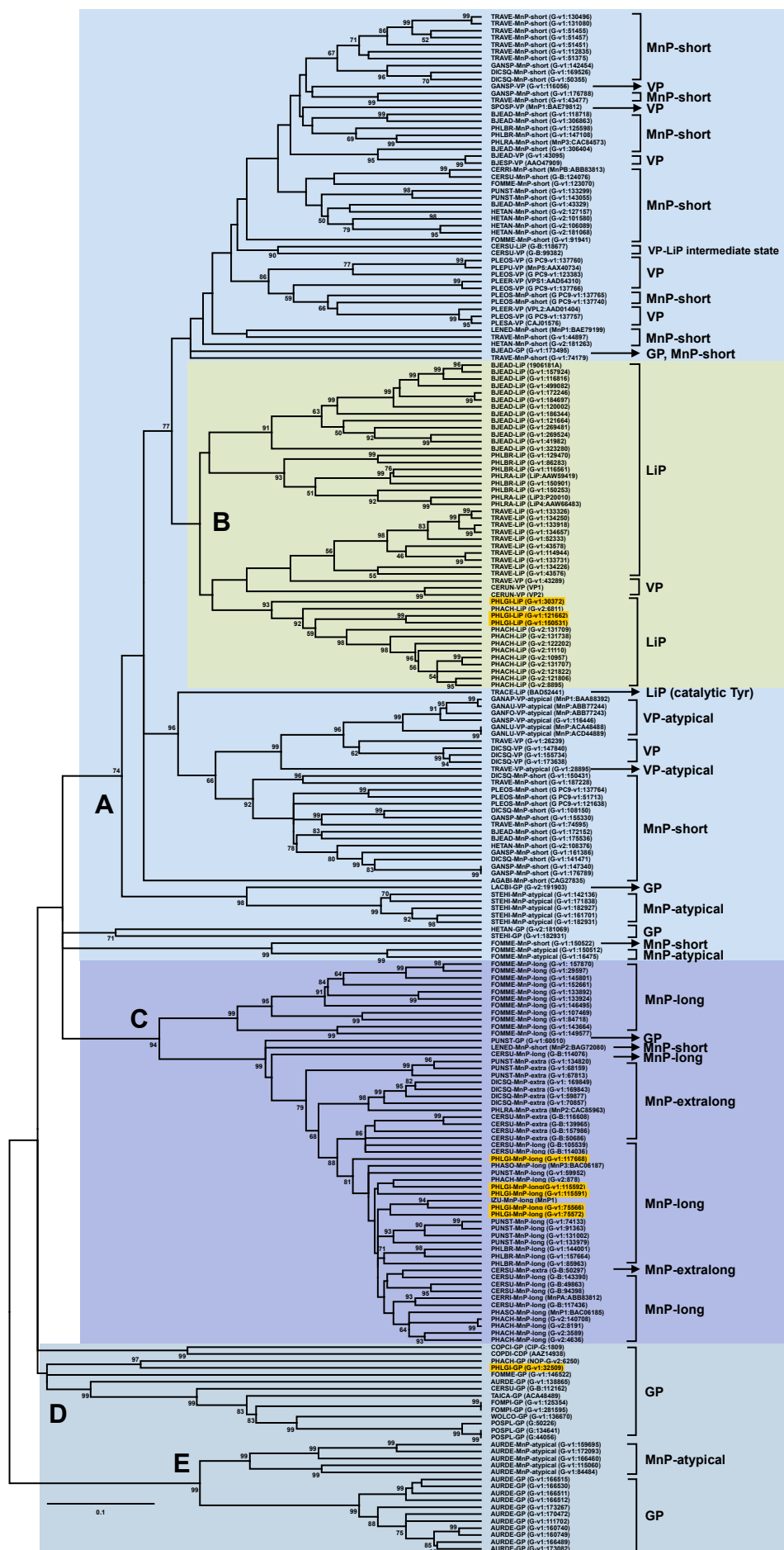

Supplement: Figure S12 — Dendrogram focused on class II heme peroxidases (a total of 219) showing evolutionary relationships and structural-functional classification. A) Short, long and extralong MnPs have a Mn2+-oxidation site formed by two glutamic and one aspartic residues, and differ in the length of the C-terminal tail; B) LiPs contain a catalytic tryptophan, with the only exception of TRACE-LiP being the “unique” ligninolytic peroxidase with a catalytic tyrosine (3); C) VPs harbor the catalytic sites described above for both MnPs and LiPs; D) GPs do not contain any of the above two catalytic sites; and E) atypical MnPs and VPs lack one of the three acidic residues forming the Mn2+-oxidation site. The analysis is described in Figure S9. Fungal abbreviations: AGABI, Agaricus bisporus; AURDE, Auricularia delicata; BJEAD, Bjerkandera adusta; BJESP, Bjerkandera sp; CERRI, Ceriporiopsis rivulosa; CERSU, Ceriporiopsis subvermispora-B; CERUN, Cerrena unicolor; COPCI, Coprinopsis cinerea; COPDI, Coprinellus disseminatus; DICSQ, Dichomitus squalens v1.0; FOMME, Fomitiporia mediterranea v1.0; FOMPI, Fomitopsis pinicola SS1 v1.0; GANAP, Ganoderma applanatum; GANAU, Ganoderma australe; GANFO, Ganoderma formosanum; GANLU, Ganoderma lucidum; GANSP, Ganoderma sp.; HETAN, Heterobasidion annosum v2.0; IZU, basidiomycete IZU-154; LACBI, Laccaria bicolor v2.0; LENED, Lentinula edodes; PHACH, Phanerochaete chrysosporium; PHASO, Phanerochaete sordida; PHLBR, Phlebia brevispora HHB-7030 SS6 v1.0; PHLGI, Phlebiopsis gigantea; PHLRA, Phlebia radiata; PLEER, Pleurotus eryngii; PLEOS, Pleurotus ostreatus; PLEPU, Pleurotus pulmonarius; PLESA, Pleurotus sapidus; POSPL, Postia placenta; PUNST, Punctularia strigosozonata v1.0; SPOSP, Spongipellis sp.; STEHI, Stereum hirsutum FP-91666 SS1 v1.0; TAICA, Taiwanofungus camphoratus; TRACE, Trametopsis cervina; TRAVE, Trametes versicolor; WOLCO, Wolfiporia cocos MD-104 SS10 v1.0. GeneBank and JGI references are shown in parentheses and P. gigantea genome references on yel [file pgen.1004759.s012.pdf]

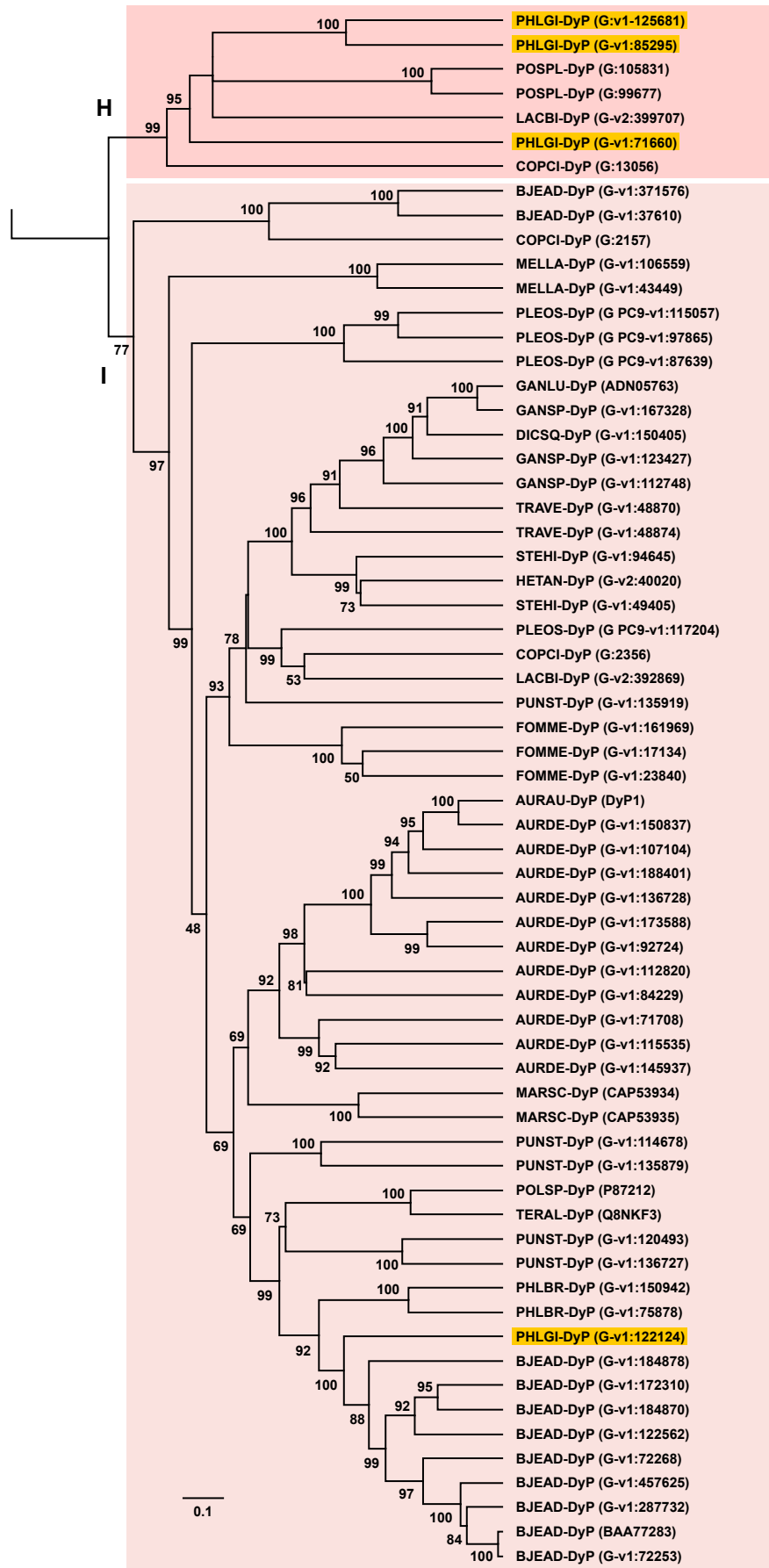

Supplement: Figure S13 — Dendrogram focused on DyP peroxidases (a total of 64) showing evolutionary relationships. The analysis is described in Figure S7. Fungal abbreviations: AURAU, Auricularia auricula-judae; AURDE, Auricularia delicata; BJEAD, Bjerkandera adusta; COPCI, Coprinopsis cinerea; DICSQ, Dichomitus squalens v1.0; FOMME, Fomitiporia mediterranea v1.0; GANLU, Ganoderma lucidum; GANSP, Ganoderma sp.; HETAN, Heterobasidion annosum v2.0; LACBI, Laccaria bicolor v2.0; MARSC, Marasmius scorodonius; MELLA, Melampsora laricis-populina v1.0; PHLBR, Phlebia brevispora HHB-7030 SS6 v1.0; PHLGI, Phlebiopsis gigantea; PLEOS, Pleurotus ostreatus; POSPL, Postia placenta; PUNST, Punctularia strigosozonata v1.0; STEHI, Stereum hirsutum FP-91666 SS1 v1.0; TERAL, Termitomyces albuminosus; TRAVE, Trametes versicolor. GenBank and JGI references are shown in parentheses and P. gigantea genome references on yellow background. (PDF) [file pgen.1004759.s013.pdf]

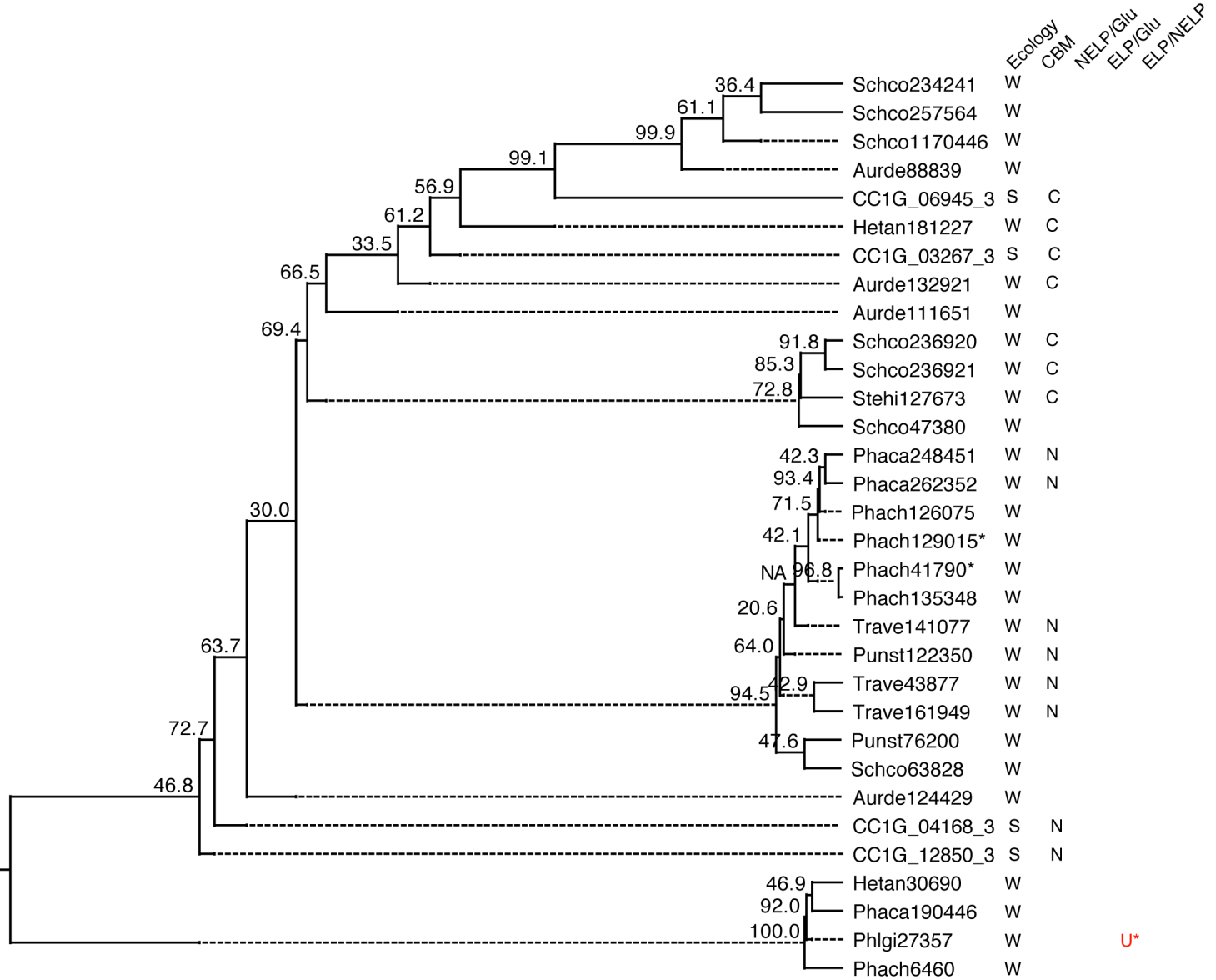

Supplement: Figure S24 — Phylogeny and differential expression of carbohydrate esterase family 1 (CE1) genes. CDS sequences were obtained from each genome database according to assigned protein IDs. Incomplete CDS sequences (partial fragments) were eliminated from the analysis. For each CE family, a multiple alignment was performed using MegAlign version 10 software. The phylogenetic tree was then constructed from the multiple alignment using Clustal W [85]. Numbers at the nodes represent bootstrap values, based on 1000 replications. Species: Aurde, Auricularia delicata SS-5; Conpu, Coniophora puteana; CC1G, Coprinopsis cinerea; Dicsq, Dichomitus squalens; Fomme, Fomitiporia mediterranea; Fompi, Fomitopsis pinicola FP-58527 SS1; Glotr, Gloeophyllum trabeum; Hetan, Heterobasidion annosum; Lacbi, Laccaria bicolor; Phaca, Phanerochaete carnosa HHB-10118; Phach, Phanerochaete chrysosporium RP78; Phlgi, Phlebiopsis gigantea; Punst, Punctularia strigosozonata; Schco, Schizophyllum commune; Serla, Serpula lacrymans S7.3; Stehi, Stereum hirsutum FP-91666 SS1; Trave, Trametes versicolor; Wolco, Wolfiporia cocos MD-104 SS10. Ecology: W, white rot; B, brown rot; M, mycorrhiza; S, non-wood decay saprotroph. Location of comprised CBM1 was indicated as N or C-terminal. Differential regulation of Phlgi CE transcripts between the cultivations tested in this study were also indicated as up- (U) or down- (D) regulated. Asterisk was accompanied if the p value was <0.05. Possible CE1 gene Phlgi_121418 was excluded as the model was severely truncated (91 amino acid residues). (PDF) [file pgen.1004759.s024.pdf]

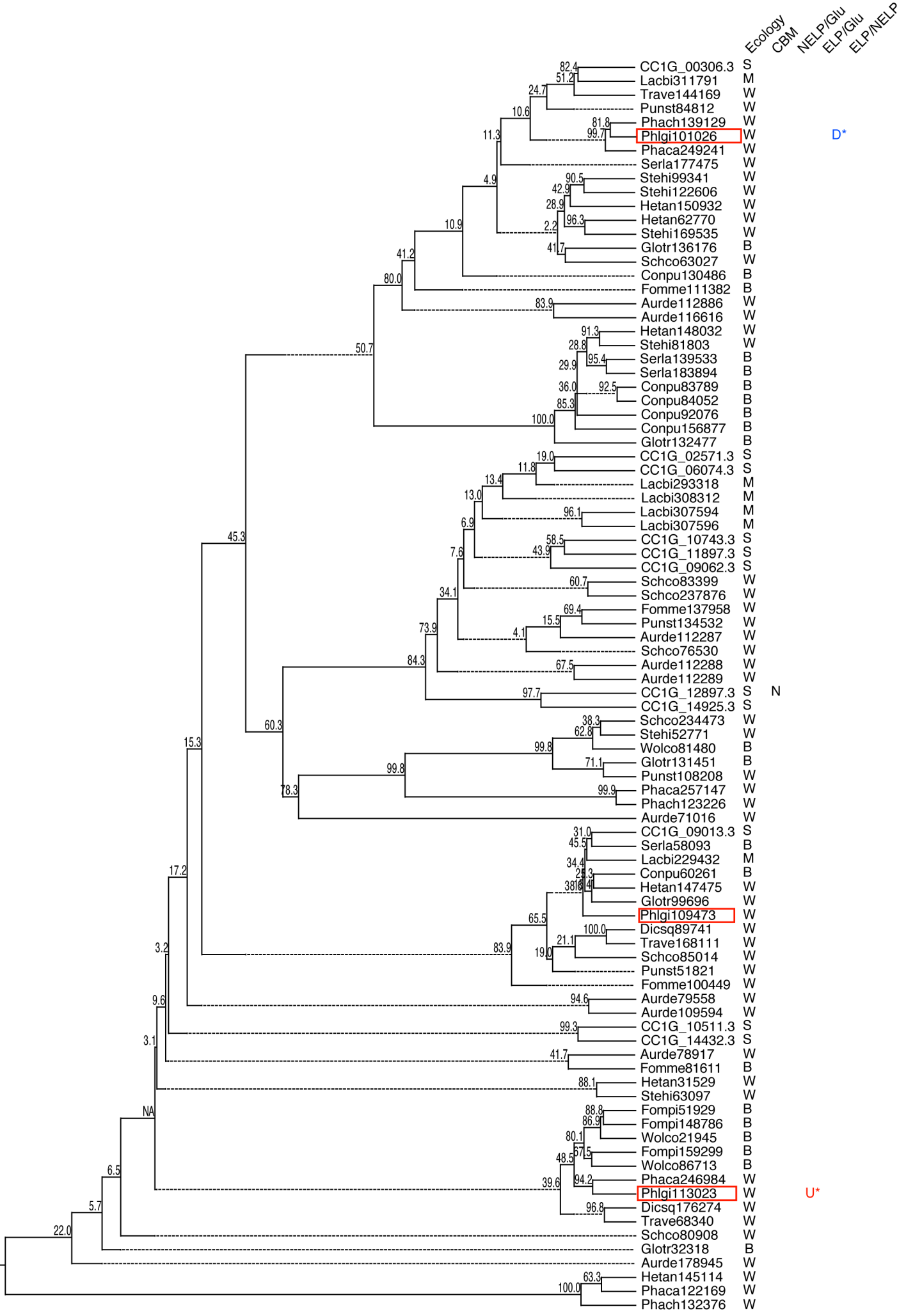

Supplement: Figure S25 — Phylogeny and differential expression of CE4 genes. Analysis and abbreviations as in Figure S24. (PDF) [file pgen.1004759.s025.pdf]

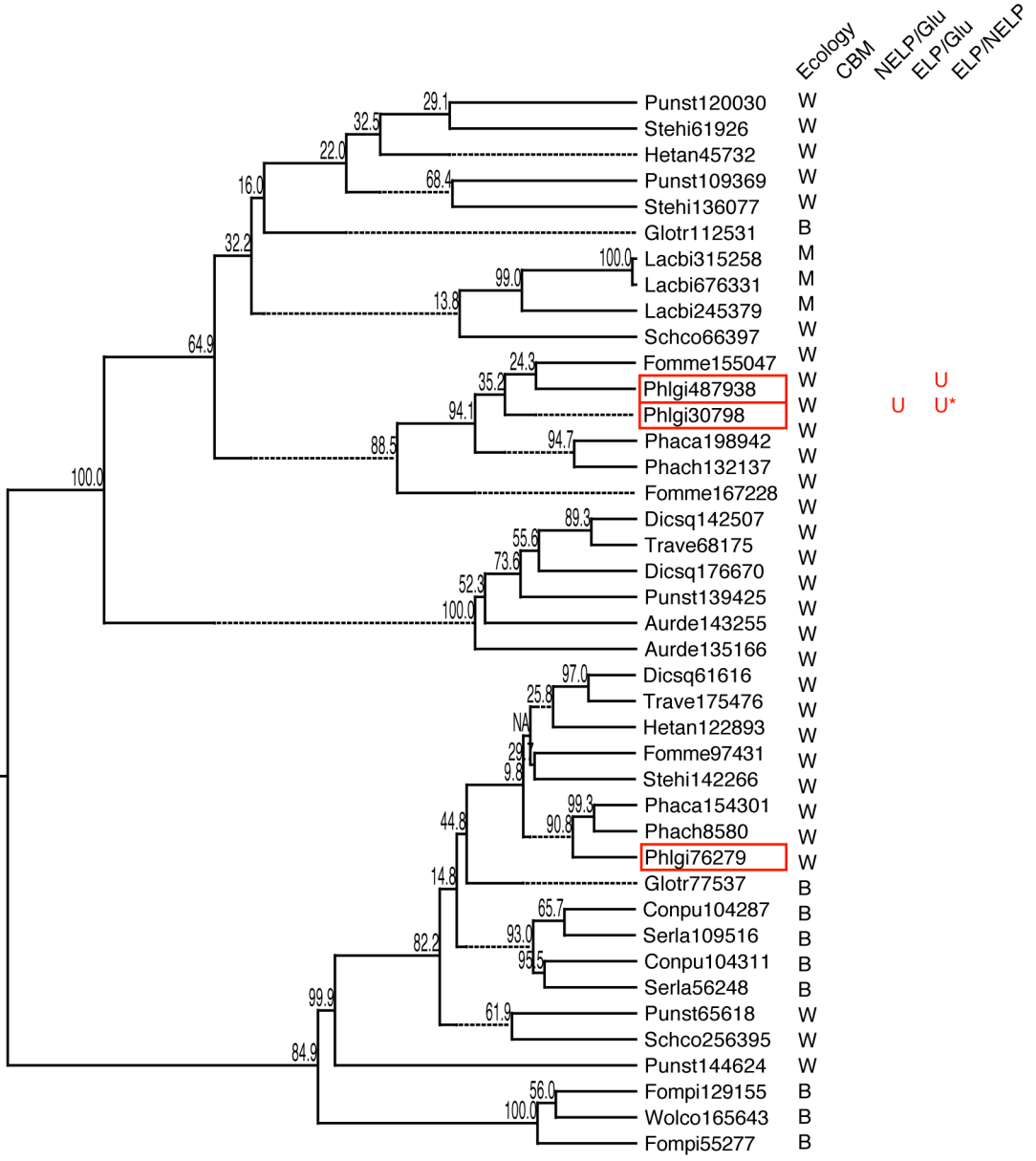

Supplement: Figure S26 — Phylogeny and differential expression of CE8 genes. Analysis and abbreviations as in Figure S24. Possible CE8 gene Phlgi_132681 was excluded as the model was severely truncated (79 residues). (PDF) [file pgen.1004759.s026.pdf]

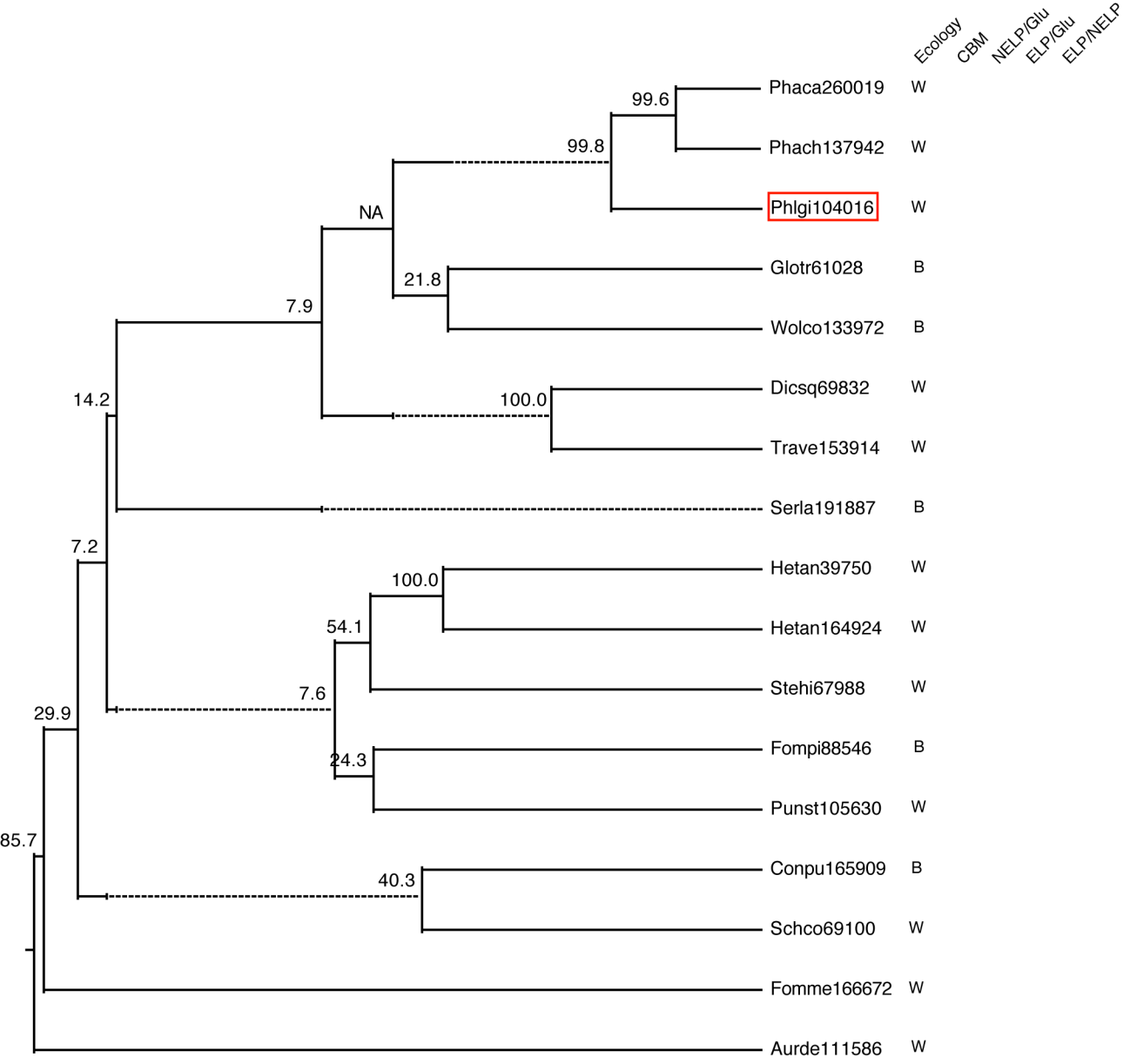

Supplement: Figure S27 — Phylogeny and differential expression of CE9 genes. Analysis and abbreviations as in Figure S24. (PDF) [file pgen.1004759.s027.pdf]

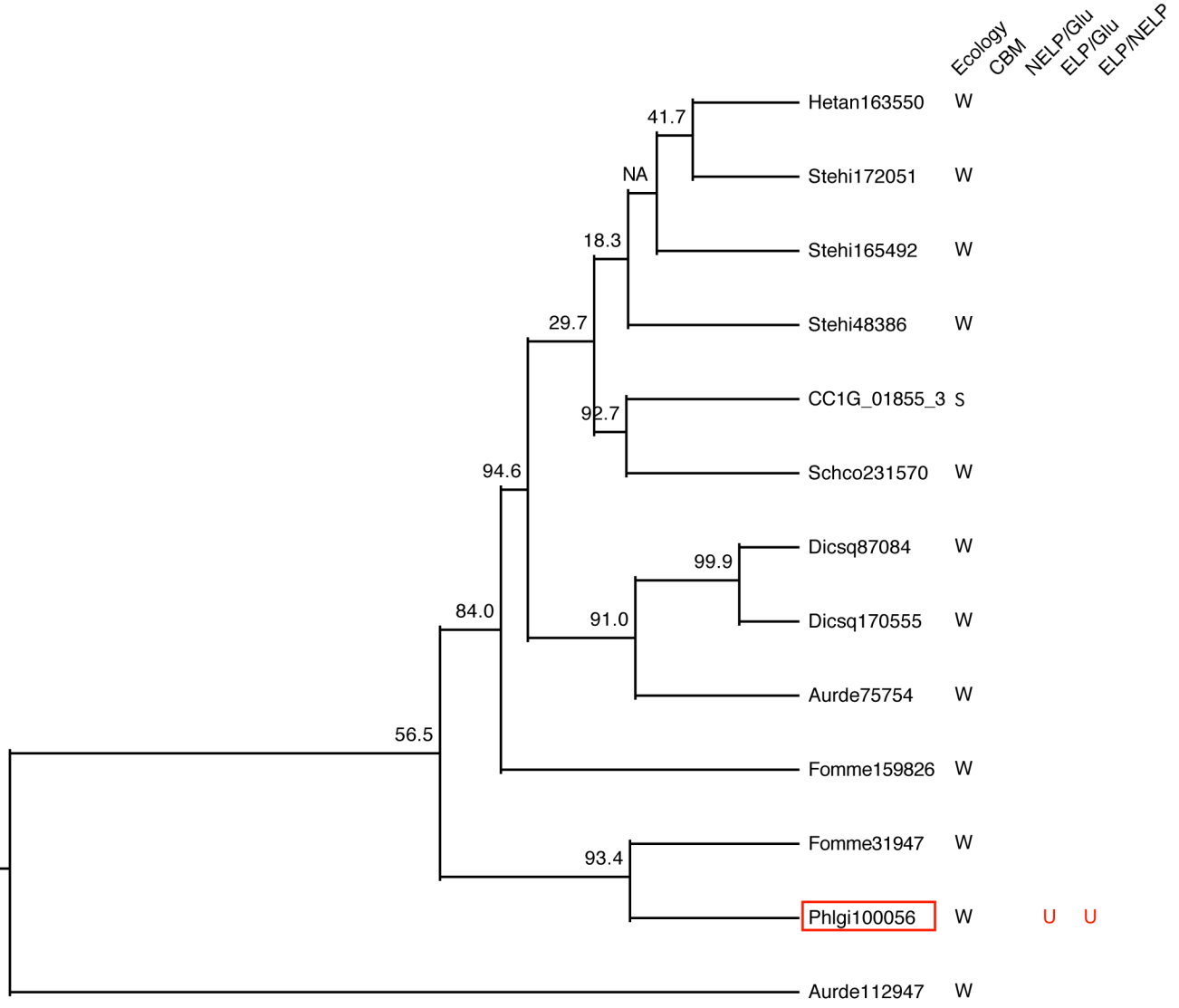

Supplement: Figure S28 — Phylogeny and differential expression of CE12 genes. Analysis and abbreviations as in Figure S24. (PDF) [file pgen.1004759.s028.pdf]

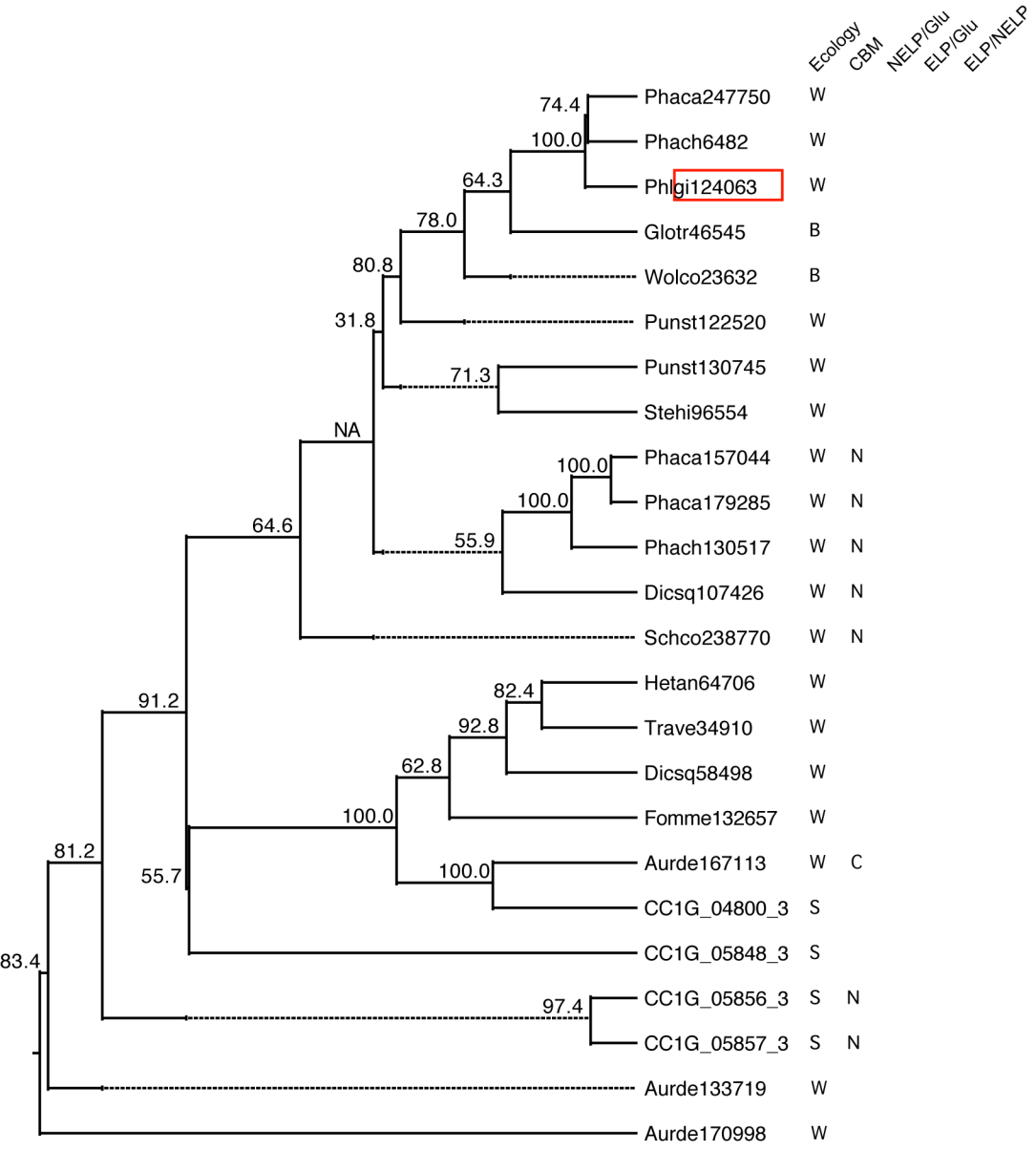

Supplement: Figure S29 — Phylogeny and differential expression of CE15 genes. Analysis and abbreviations as in Figure S24. (PDF) [file pgen.1004759.s029.pdf]

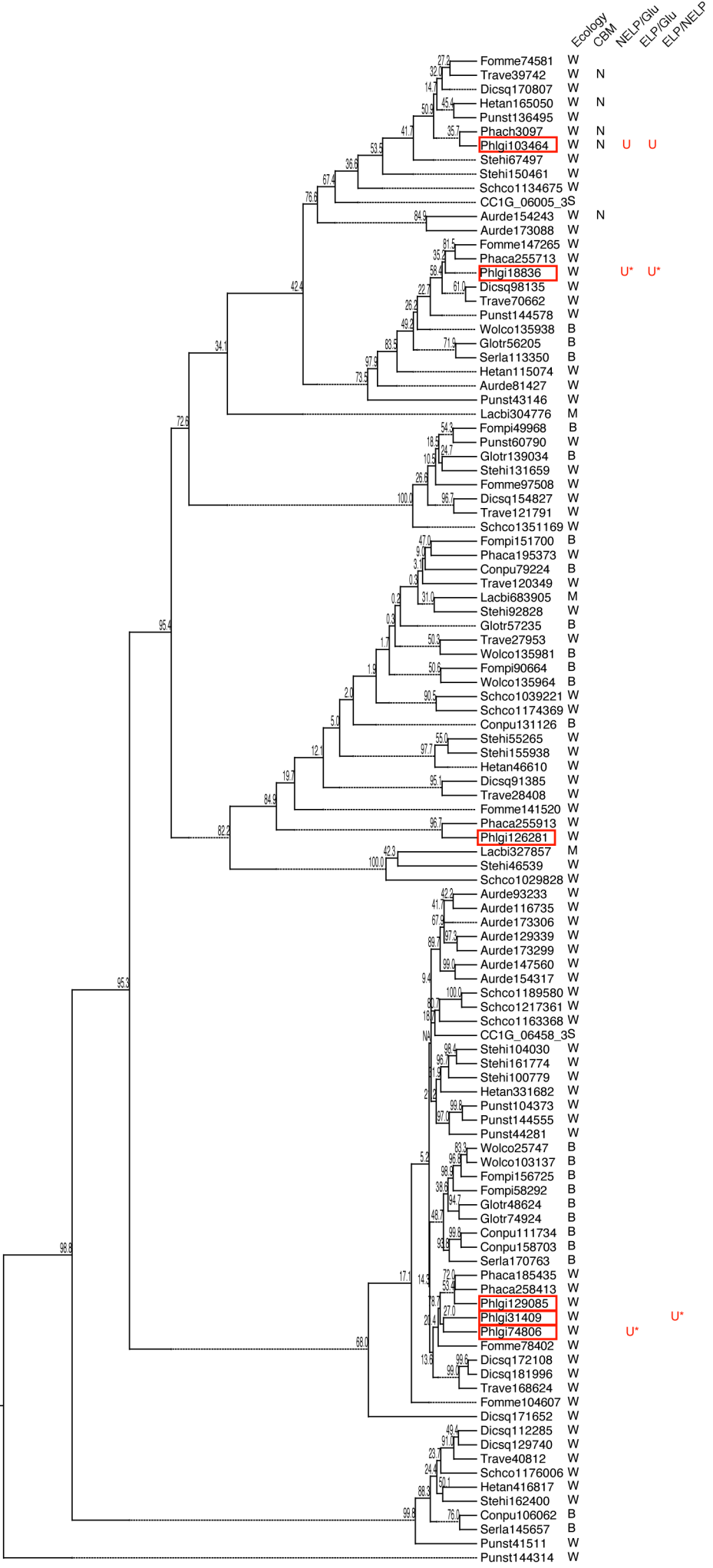

Supplement: Figure S30 — Phylogeny and differential expression of CE16 genes. Analysis and abbreviations as in Figure S24. Possible CE16 gene Phlgi_73119 was excluded as the model was severely truncated (95 residues). (PDF) [file pgen.1004759.s030.pdf]
