# Supplementary material for: Analysis of the Phlebiopsis gigantea Genome, Transcriptome and Secretome Provides Insight into Its Pioneer Colonization Strategies of Wood
Source: PLoS Genet. 2014 Dec 4;10(12):e1004759. doi: 10.1371/journal.pgen.1004759 (PMC4256170; doi:10.1371/journal.pgen.1004759)
Supplement: Table S1 — Genome assembly. (DOCX) [file pgen.1004759.s036.docx]

| Table S1. Genome Assembly | |
| --- | --- |
| Genome Assembly size (Mbp} | 30.14 |
| Sequencing read coverage depth | 144.9 |
| # of contigs | 1195 |
| # of scaffolds | 573 |
| # of scaffolds >= 2Kbp | 506 |
| Scaffold N50 | 72 |
| Scaffold L50 (Mbp) | 0.12 |
| # of gaps | 622 |
| % of scaffold length in gaps | 1.5% |
| Three largest Scaffolds (Mbp) | 0.61, 0.54, 0.46 |
