# Supplementary material for: Analysis of the Phlebiopsis gigantea Genome, Transcriptome and Secretome Provides Insight into Its Pioneer Colonization Strategies of Wood
Source: PLoS Genet. 2014 Dec 4;10(12):e1004759. doi: 10.1371/journal.pgen.1004759 (PMC4256170; doi:10.1371/journal.pgen.1004759)
Supplement: Table S2 — Annotation. (DOCX) [file pgen.1004759.s037.docx]

| Table S2. Annotations | | |
| --- | --- | --- |
| Gene Models | Filtered Models2 |  |
| length (bp) of: | average | median |
| gene | 1714 | 1459 |
| transcript | 1380 | 1164 |
| exon | 230 | 145 |
| intron | 69 | 56 |
| description: |  |  |
| protein length (aa) | 411 | 338 |
| exons per gene | 6.00 | 5 |
| # of gene models | 11891 |  |
