# Supplementary material for: Analysis of the Phlebiopsis gigantea Genome, Transcriptome and Secretome Provides Insight into Its Pioneer Colonization Strategies of Wood
Source: PLoS Genet. 2014 Dec 4;10(12):e1004759. doi: 10.1371/journal.pgen.1004759 (PMC4256170; doi:10.1371/journal.pgen.1004759)
Supplement: Table S3 — Transcript profiles of response regulator genes clustered on scaffold. (DOCX) [file pgen.1004759.s038.docx]

| **Table S3**. Transcript profiles of response regulator genes clustered on scaffold 1 | | | | | | | | | | | | | |
| --- | --- | --- | --- | --- | --- | --- | --- | --- | --- | --- | --- | --- | --- |
|  |  |  |  |  | RPKM value | | | NELP/Glu | | NELP/ELP | | ELP/Glu | |
| Pro Id | Type | Description | strand | start | NELP | ELP | Glu | Prob | R | Prob | R | Prob | R |
| 36445 |  | Fungal specific transcription factor, conserved, related to NCU01478 | - | 353 | 18.90 | 24.91 | 14.83 | 0.34 | 1.27 | 0.24 | 0.76 | 0.11 | 1.68 |
| 74327 |  | Fungal specific transcription factor, conserved, related to NCU01478 and NCU02934 (somewhat up on miscantus) | - | 4978 | 6.74 | 13.11 | 7.57 | 0.75 | 0.89 | 0.02 | 0.51 | 0.16 | 1.73 |
| 60579 |  | Fungal specific transcription factor, conserved, related to NCU01478 | + | 18749 | 80.23 | 72.36 | 48.01 | 0.16 | 1.67 | 0.43 | 1.11 | 0.24 | 1.51 |
| 92206 |  | Fungal specific transcription factor, conserved, related to NCU01478 (NCR: transcribed but not regulated) | + | 23699 | 68.51 | 73.54 | 41.51 | 0.19 | 1.65 | 0.68 | 0.93 | 0.15 | 1.77 |
| 151979 |  | Fungal specific transcription factor, conserved, related to *N crassa acu-15* (NCU06656) (NCR: somewhat up on miscantus) | + | 28005 | 69.93 | 139.13 | 12.13 | 0.03 | 5.76 | 0.50 | 0.50 | 0.08 | 11.47 |
| 152085 |  | Fungal specific transcription factor, conserved, also related to NCU01478 | + | 31062 | 83.30 | 141.01 | 13.66 | 0.01 | 6.10 | 0.49 | 0.59 | 0.05 | 10.32 |
| 92212 |  | Fungal specific transcription factor, conserved, related to NCU01478 (Schmoll et al., 2012), TR_2148 (carbon regulated, enhanced on cellulose) | + | 34212 | 56.46 | 71.26 | 34.80 | 0.12 | 1.62 | 0.17 | 0.79 | 0.06 | 2.05 |
| 152158 |  | Hypothetical | - | 38909 | 5.32 | 3.83 | 2.28 | 0.17 | 2.34 | 0.18 | 1.39 | 0.36 | 1.68 |
| 152163 |  | Hypothetical | - | 40895 | 19.60 | 29.68 | 7.08 | 0.02 | 2.77 | 0.10 | 0.66 | 0.01 | 4.19 |
| 152182 | RR |  | - | 42080 | 3.31 | 4.59 | 1.97 | 0.23 | 1.68 | 0.14 | 0.72 | 0.09 | 2.33 |
| 152213 | RR |  | + | 45224 | 6.17 | 9.37 | 3.33 | 0.25 | 1.85 | 0.03 | 0.66 | 0.09 | 2.81 |
| 119846 | RR |  | - | 48611 | 5.33 | 3.94 | 3.28 | 0.22 | 1.63 | 0.12 | 1.35 | 0.64 | 1.20 |
| 490114 | RR |  | - | 50377 | 3.90 | 7.35 | 3.13 | 0.45 | 1.25 | 0.07 | 0.53 | 0.05 | 2.35 |
| 119848 |  | Conserved hypothetical | + | 52065 | 21.33 | 27.59 | 26.99 | 0.41 | 0.79 | 0.23 | 0.77 | 0.92 | 1.02 |
| 30927 |  | Putative galactokinase | - | 53899 | 3.07 | 1.73 | 4.66 | 0.18 | 0.66 | 0.11 | 1.78 | 0.06 | 0.37 |
| 152279 |  | Hypothetical | + | 56755 | 0.23 | 0.19 | 0.38 | 0.24 | 0.61 | 0.74 | 1.19 | 0.24 | 0.51 |
| 128901 |  | Possible lectin | + | 58838 | 0.96 | 0.84 | 1.46 | 0.19 | 0.66 | 0.81 | 1.14 | 0.36 | 0.58 |
| 128902 |  | Aldose epimerase | + | 60286 | 5.97 | 1.45 | 3.75 | 0.42 | 1.59 | 0.01 | 4.13 | 0.16 | 0.39 |
| 14562 |  | Conserved hypothetical | + | 63336 | 5.83 | 5.18 | 5.72 | 0.93 | 1.02 | 0.66 | 1.13 | 0.78 | 0.91 |
| 152334 | RR |  | - | 66571 | 27.69 | 30.07 | 17.88 | 0.37 | 1.55 | 0.71 | 0.92 | 0.29 | 1.68 |
| 128904 | RR |  | + | 69489 | 32.84 | 31.44 | 26.23 | 0.51 | 1.25 | 0.75 | 1.04 | 0.60 | 1.20 |
| 119856 |  | DNA binding domain, putative AAA ATPase, several homologues in P placenta, related to N. crassa NCU02965 (DNO-1; DNA replication origin 1; somewhat light regulated). | + | 73415 | 4.37 | 3.44 | 2.28 | 0.10 | 1.91 | 0.41 | 1.27 | 0.20 | 1.51 |
| 152434 |  | Hypothetical | - | 76433 | 2.63 | 2.04 | 2.70 | 0.95 | 0.97 | 0.51 | 1.29 | 0.35 | 0.76 |
| 152457 | RR |  | - | 77043 | 11.73 | 12.08 | 12.22 | 0.88 | 0.96 | 0.87 | 0.97 | 0.97 | 0.99 |
| 36456 | RR |  | - | 79742 | 6.64 | 5.35 | 5.04 | 0.23 | 1.32 | 0.16 | 1.24 | 0.82 | 1.06 |
| 119859 |  | Hypothetical | - | 83037 | 0.45 | 0.20 | 0.30 | 0.56 | 1.50 | 0.12 | 2.26 | 0.48 | 0.66 |
| 152532 |  | Hypothetical conserved in basidiomycetes | + | 84189 | 9.46 | 5.18 | 7.54 | 0.41 | 1.25 | 0.04 | 1.83 | 0.15 | 0.69 |
| 108310 |  | Mitochondrial substrate carrier | - | 86483 | 10.66 | 11.13 | 12.16 | 0.70 | 0.88 | 0.83 | 0.96 | 0.83 | 0.91 |
| 25105 |  | Contains winged helix DNA binding domain | + | 87878 | 4.83 | 2.44 | 2.77 | 0.30 | 1.75 | 0.12 | 1.98 | 0.86 | 0.88 |
| 36458 |  | Transcriptional regulator, possibly involved in chromatin rearrangement, contains HMG box | - | 89279 | 256.76 | 61.38 | 64.27 | 0.04 | 4.00 | 0.00 | 4.18 | 0.92 | 0.96 |
| 36459 |  | Putative C-type lectin, conserved in basidiomycetes | + | 90330 | 13.17 | 12.13 | 1230.72 | 0.00 | 0.01 | 0.76 | 1.09 | 0.00 | 0.01 |

The putative aldose epimerase (Phlgi1_128902) may be upregulated on LP but RPKM values were below 10 (red highlighting). Statistically significant (P<0.01) values appear with yellow highlight
