# Supplementary material for: Analysis of the Phlebiopsis gigantea Genome, Transcriptome and Secretome Provides Insight into Its Pioneer Colonization Strategies of Wood
Source: PLoS Genet. 2014 Dec 4;10(12):e1004759. doi: 10.1371/journal.pgen.1004759 (PMC4256170; doi:10.1371/journal.pgen.1004759)
Supplement: Table S4 — Copper radical oxidases (CROs) of P. Gigantea. (DOCX) [file pgen.1004759.s039.docx]

| **Table S4** Copper radical oxidases (CROs) of *P. gigantea* | | | | |
| --- | --- | --- | --- | --- |
| Phlgi Protein ID | Gene location | strand | N-terminal characterization |  |
| 380490 | 43:65759-68018 | minus | 0 Tm; SignalP 22/23 |  |
| 327975 | 3:77544-80926-80926 | minus | 1 transmembrane domain, Signalp 86/87 |  |
| 453167 | 79:24216-26908 | minus | 0 TM; Signalp 22/23 |  |
| 19348 | 72:14332-16862 | minus | 0 TM; SignalP 16/17 |  |
| 128606 | 94:59684-62642 | plus | 0 TM; SignalP 16/17 |  |
| 513029 | 65:107892-112595 | minus | 0 TM; Signalp 21/22 |  |
| 118353  /xx gap xx/  71528 | scaffold_65:126629-126950  xxx  scaffold_65:124504-124659 |  |  |  |
