# Supplementary material for: Analysis of the Phlebiopsis gigantea Genome, Transcriptome and Secretome Provides Insight into Its Pioneer Colonization Strategies of Wood
Source: PLoS Genet. 2014 Dec 4;10(12):e1004759. doi: 10.1371/journal.pgen.1004759 (PMC4256170; doi:10.1371/journal.pgen.1004759)
Supplement: Table S5 — Expression of P. gigantea GMC oxidoreductases in solvent extracted lodgepole pine wood (ELP) and non-extracted lodgepole pine wood (NELP). (DOCX) [file pgen.1004759.s040.docx]

| **Table S5**. Expression of *Phlebiopsis gigantea* GMC oxidoreductases in solvent extracted lodgepole pine (ELP) and non-extracted lodgepole pine (NELP)^1^ | | | | | | | | | | | | | | | | |
| --- | --- | --- | --- | --- | --- | --- | --- | --- | --- | --- | --- | --- | --- | --- | --- | --- |
|  |  | LC-MS/MS emPAI values | | | | | | RNAseq | | | | | | | | |
|  |  | NELP | | | ELP | | | RPKM values | | | NELP/Glu | | NELP/ELP | | ELP/Glu | |
| Pro ID | Putative function | Day 5 | Day 7 | Day 9 | Day 5 | Day 7 | Day 9 | NELP | ELP | Glu | Prob | Ratio | Prob | Ratio | Prob | Ratio |
| 121514 | AAO | 0.00 | 0.00 | 0.00 | 0.15 | 0.00 | 0.00 | 18.03 | 21.07 | 35.08 | 0.061 | 0.51 | 0.411 | 0.86 | 0.097 | 0.60 |
| 128071 | AAO | 0.59 | 0.16 | 0.04 | 3.45 | 6.99 | 5.97 | 9.26 | 13.05 | 9.39 | 0.970 | 0.99 | 0.165 | 0.71 | 0.291 | 1.39 |
| 120749 | MOX | 0.00 | 0.00 | 0.00 | 0.00 | 2.86 | 2.17 | 10480.06 | 7015.26 | 1014.82 | 0.251 | 10.33 | 0.155 | 1.49 | 0.316 | 6.91 |
| 108516 | MOX |  |  |  |  |  |  | 29.95 | 22.52 | 7.22 | 0.008 | 4.15 | 0.363 | 1.33 | 0.051 | 3.12 |
| 72751 | MOX |  |  |  |  |  |  | 14.02 | 14.89 | 3.81 | 0.209 | 3.68 | 0.834 | 0.94 | 0.176 | 3.91 |
| 99876 | CDH | 5.31 | 1.37 | 0.63 | 5.63 | 0.12 | 0.23 | 601.86 | 327.35 | 11.95 | 0.000 | 50.38 | 0.065 | 1.84 | 0.001 | 27.40 |
| 130349 | POX | 0.00 | 0.00 | 0.00 | 0.00 | 0.62 | 0.00 | 17.75 | 22.35 | 12.65 | 0.185 | 1.40 | 0.155 | 0.79 | 0.067 | 1.77 |
| 128108 | GOX | 0.07 | 0.21 | 0.18 | 0.07 | 0.00 | 0.00 | 41.92 | 46.48 | 6.92 | 0.025 | 6.06 | 0.514 | 0.90 | 0.017 | 6.72 |
| 89048 | AAD |  |  |  |  |  |  | 312.71 | 155.91 | 87.27 | 0.010 | 3.58 | 0.029 | 2.01 | 0.092 | 1.79 |
| 115124 | AAD |  |  |  |  |  |  | 2.55 | 2.46 | 2.71 | 0.783 | 0.94 | 0.843 | 1.03 | 0.678 | 0.91 |
| 127405 | AAD |  |  |  |  |  |  | 82.72 | 94.14 | 56.10 | 0.128 | 1.47 | 0.510 | 0.88 | 0.118 | 1.68 |
| 406426 | AAD |  |  |  |  |  |  | 2.77 | 3.18 | 2.56 | 0.793 | 1.08 | 0.680 | 0.87 | 0.629 | 1.24 |
| 147847 | AAD |  |  |  |  |  |  | 2.88 | 4.32 | 1.97 | 0.227 | 1.46 | 0.150 | 0.67 | 0.081 | 2.19 |
| 101518 | AAD |  |  |  |  |  |  | 37.68 | 63.87 | 70.51 | 0.280 | 0.53 | 0.511 | 0.59 | 0.926 | 0.91 |
| 148078 | AAD |  |  |  |  |  |  | 0.48 | 0.56 | 0.71 | 0.754 | 0.67 | 0.723 | 0.85 | 0.857 | 0.78 |
| 105456 | AAD |  |  |  |  |  |  | 193.37 | 227.73 | 92.05 | 0.149 | 2.10 | 0.653 | 0.85 | 0.078 | 2.47 |

^1^Particularly abundant transcripts (>100 RPKM values) and significant regulation (P<0.05; >2-fold) are highlighted in red and yellow, respectively. Abbreviations: AAO, aryl alcohol oxidase; MOX, methanol oxidase; CDH, cellobiose dehydrogenase; POX, pyranose 2-oxidase; GOX, glucose oxidase; AAD, aryl alcohol dehydrogenase.
