# Supplementary material for: Analysis of the Phlebiopsis gigantea Genome, Transcriptome and Secretome Provides Insight into Its Pioneer Colonization Strategies of Wood
Source: PLoS Genet. 2014 Dec 4;10(12):e1004759. doi: 10.1371/journal.pgen.1004759 (PMC4256170; doi:10.1371/journal.pgen.1004759)
Supplement: Table S6 — Multicopper oxidases of P. gigantea. (DOCX) [file pgen.1004759.s041.docx]

| **Table S6.** Multicopper oxidases of *P. gigantea* | | |
| --- | --- | --- |
| *P. gigantea* ID | Position | Description, notes |
| 29483  (Alt. model=  534797) | scaffold_40:41138-44168 | This model appears similar to a MCO of *Phanerochaete flavido-alba* with laccase activity ([34](#_ENREF_34)). Nevertheless, L1 and L2 signatures (([80](#_ENREF_80)); Figure S21) are more similar to *P. chrysosporium* MCOs rather than laccases motifs. The same observation can be concluded from signature L4. Phlgi1_29483 possesses a putative N-terminal signal peptide. So most likely, the encoded protein is secreted. Irrespective, it cannot be classified as a canonical laccase.  No transcript accumulation. No detectable peptides. |
| 32068  (Alt. model= 534793) | scaffold_217:1620-4283 | Similar to MCO2 from *P. chrysosporium*. Nevertheless, it possesses a putative N-terminal TM domain (0.99 prob N-in). The L2 signature of ID29483 and ID32068 looks different from *P. chrysosporium* MCOs. The encoded MCO enzyme putatively possesses ferroxidase activity .  Peptide evidence after 7 days growth in ELP. Transcripts elevated in LP media, but not statitistically significant. |
| 38701 | scaffold_61:102144-102365 | Similar to a Fet3 protein. The model is incomplete, and no FTR1 sequence was identified in the neighborhood. The L4 signature is in agreement with this observation. |
| 77659 | scaffold_181:611-2793 | Fet3, single TM domain (C-terminus). In opposite transcriptional orientation with ID77664 (FTR1), the iron permease. This latter protein possesses as expected, 7TM domains. Thus, this pair seems to be the canonical Fet3/Ftr1 system. No significant transcript accumulation observed. |
| 110266  (Alt. model= 534796) | scaffold_156:6181-9001 | Similar to MCO4 from *P. chrysosporium*. Peptides were detected. No significant transcript regulation. |
| 129839  (Alt. model= 534795) | scaffold_156:12623-15622 | Similar to *P. chrysosporium* MCO4. Phlgi1_129839 also possesses a putative N-terminal TM domain (1) (Signal P predicts a signal peptide). Several peptides were detected. Significant transcript and protein accumulation in ELP relative to NELP. |
