# Supplementary material for: Analysis of the Phlebiopsis gigantea Genome, Transcriptome and Secretome Provides Insight into Its Pioneer Colonization Strategies of Wood
Source: PLoS Genet. 2014 Dec 4;10(12):e1004759. doi: 10.1371/journal.pgen.1004759 (PMC4256170; doi:10.1371/journal.pgen.1004759)
Supplement: Table S7 — Glycoside hydrolase comparisons of brown-rot (BR) and white-rot (WR) fungi. (DOCX) [file pgen.1004759.s042.docx]

| \| **Table S7.** Glycoside hydrolase comparisons of brown-rot (BR) and white-rot (WR) fungi \| \| \| \| \| \| \| \| \| \| \| \| \| \| \| \| \| \| \| \| \| \| \| \| \| \| \| \| --- \| --- \| --- \| --- \| --- \| --- \| --- \| --- \| --- \| --- \| --- \| --- \| --- \| --- \| --- \| --- \| --- \| --- \| --- \| --- \| --- \| --- \| --- \| --- \| --- \| --- \| --- \| \|  \|  \| **Glycoside hydrolase (GH) family** \| \| \| \| \| \| \| \| \| \| \| \| \| \| \| \| \| \| \| \| \| \| \| \| \| \| **Decay** \| **Species** \| **1** \| **2** \| **3** \| **5** \| **6** \| **7** \| **9** \| **10** \| **11** \| **12** \| **13** \| **15** \| **16** \| **17** \| **18** \| **20** \| **23** \| **25** \| **26** \| **27** \| **28** \| **29** \| **30** \| **31** \| **32** \| \| **BR** \| ***Postia placenta Mad-698-R*** \| **2** \| **3** \| **6** \| **17** \| **0** \| **0** \| **0** \| **3** \| **0** \| **2** \| **7** \| **2** \| **24** \| **2** \| **11** \| **3** \| **1** \| **0** \| **0** \| **3** \| **8** \| **0** \| **3** \| **4** \| **0** \| \| **BR** \| ***Fomitopsis pinicola*** \| **2** \| **4** \| **12** \| **19** \| **0** \| **0** \| **0** \| **2** \| **0** \| **2** \| **7** \| **4** \| **28** \| **3** \| **19** \| **4** \| **1** \| **0** \| **0** \| **4** \| **12** \| **0** \| **10** \| **5** \| **3** \| \| **BR** \| ***Wolfiporia cocos*** \| **1** \| **3** \| **8** \| **18** \| **0** \| **0** \| **0** \| **4** \| **0** \| **2** \| **11** \| **2** \| **19** \| **2** \| **11** \| **2** \| **1** \| **0** \| **0** \| **3** \| **9** \| **0** \| **2** \| **5** \| **0** \| \| **BR** \| ***Gloeophyllum trabeum*** \| **5** \| **4** \| **11** \| **19** \| **0** \| **0** \| **1** \| **3** \| **0** \| **2** \| **9** \| **2** \| **29** \| **2** \| **11** \| **4** \| **1** \| **2** \| **0** \| **3** \| **10** \| **1** \| **3** \| **5** \| **1** \| \| **BR** \| ***Coniophora puteana*** \| **3** \| **5** \| **13** \| **21** \| **2** \| **2** \| **1** \| **3** \| **0** \| **4** \| **6** \| **2** \| **24** \| **4** \| **28** \| **4** \| **1** \| **2** \| **0** \| **4** \| **13** \| **4** \| **7** \| **13** \| **2** \| \| **BR** \| ***Dacryopinax sp.*** \| **1** \| **3** \| **9** \| **24** \| **0** \| **0** \| **1** \| **3** \| **0** \| **1** \| **11** \| **2** \| **14** \| **3** \| **7** \| **3** \| **2** \| **0** \| **1** \| **2** \| **6** \| **2** \| **4** \| **6** \| **1** \| \| **MP** \| ***Tremella mesenterica*** \| **1** \| **1** \| **3** \| **13** \| **0** \| **0** \| **1** \| **0** \| **0** \| **0** \| **10** \| **2** \| **9** \| **1** \| **4** \| **1** \| **1** \| **0** \| **0** \| **0** \| **0** \| **0** \| **0** \| **2** \| **0** \| \| **WR** \| ***Dichomitus squalens*** \| **4** \| **4** \| **8** \| **19** \| **1** \| **4** \| **0** \| **5** \| **0** \| **3** \| **10** \| **2** \| **35** \| **2** \| **21** \| **4** \| **1** \| **2** \| **0** \| **6** \| **7** \| **0** \| **2** \| **6** \| **2** \| \| **WR** \| ***Trametes versicolor*** \| **2** \| **5** \| **13** \| **22** \| **1** \| **4** \| **1** \| **6** \| **0** \| **5** \| **7** \| **4** \| **31** \| **3** \| **20** \| **6** \| **1** \| **1** \| **0** \| **4** \| **11** \| **0** \| **4** \| **5** \| **3** \| \| **WR** \| ***Fomitiporia mediterranea*** \| **5** \| **2** \| **8** \| **20** \| **2** \| **2** \| **1** \| **4** \| **0** \| **3** \| **6** \| **1** \| **28** \| **4** \| **17** \| **6** \| **1** \| **1** \| **0** \| **4** \| **17** \| **0** \| **2** \| **5** \| **0** \| \| **WR** \| ***Auricularia delicata*** \| **1** \| **7** \| **14** \| **43** \| **2** \| **8** \| **0** \| **4** \| **3** \| **1** \| **10** \| **2** \| **44** \| **7** \| **36** \| **4** \| **2** \| **1** \| **0** \| **5** \| **14** \| **3** \| **1** \| **11** \| **2** \| \| **WR** \| ***Punctularia strigosozonata*** \| **1** \| **4** \| **14** \| **18** \| **1** \| **5** \| **1** \| **5** \| **1** \| **2** \| **10** \| **4** \| **26** \| **2** \| **14** \| **6** \| **1** \| **1** \| **0** \| **5** \| **13** \| **1** \| **3** \| **8** \| **1** \| \| **WR** \| ***Heterobasidion annosum*** \| **2** \| **3** \| **12** \| **16** \| **1** \| **1** \| **1** \| **2** \| **0** \| **4** \| **8** \| **5** \| **23** \| **2** \| **10** \| **4** \| **1** \| **0** \| **0** \| **4** \| **8** \| **2** \| **2** \| **10** \| **1** \| \| **WR** \| ***Stereum hirsutum*** \| **3** \| **3** \| **17** \| **20** \| **1** \| **3** \| **1** \| **6** \| **1** \| **5** \| **14** \| **3** \| **26** \| **4** \| **21** \| **4** \| **1** \| **0** \| **0** \| **5** \| **17** \| **4** \| **5** \| **8** \| **1** \| \| **WR** \| ***Ganoderma sp. 10597*** \| **3** \| **3** \| **13** \| **18** \| **1** \| **3** \| **1** \| **10** \| **0** \| **3** \| **8** \| **3** \| **36** \| **3** \| **38** \| **5** \| **1** \| **2** \| **0** \| **3** \| **10** \| **0** \| **3** \| **7** \| **1** \| \| **WR** \| ***Bjerkandera adusta*** \| **2** \| **3** \| **9** \| **19** \| **1** \| **5** \| **1** \| **4** \| **0** \| **2** \| **10** \| **2** \| **21** \| **3** \| **16** \| **4** \| **1** \| **1** \| **0** \| **3** \| **6** \| **0** \| **1** \| **4** \| **0** \| \| **WR** \| ***Phlebia brevispora*** \| **2** \| **2** \| **8** \| **23** \| **1** \| **4** \| **1** \| **8** \| **0** \| **2** \| **8** \| **4** \| **30** \| **2** \| **22** \| **6** \| **1** \| **1** \| **0** \| **2** \| **5** \| **1** \| **2** \| **5** \| **0** \| \| **WR** \| ***Phanerochaete_carnosa*** \| **2** \| **2** \| **11** \| **24** \| **1** \| **6** \| **1** \| **5** \| **1** \| **3** \| **10** \| **2** \| **24** \| **2** \| **11** \| **3** \| **1** \| **0** \| **0** \| **3** \| **4** \| **0** \| **3** \| **8** \| **0** \| \| **WR** \| ***Ceriporiopsis subvermispora*** \| **3** \| **4** \| **6** \| **18** \| **1** \| **3** \| **0** \| **6** \| **1** \| **2** \| **7** \| **3** \| **24** \| **3** \| **14** \| **3** \| **1** \| **1** \| **0** \| **4** \| **6** \| **0** \| **1** \| **5** \| **0** \| \| **WR** \| ***Phanerochaete chrysosporium*** \| **2** \| **2** \| **11** \| **19** \| **1** \| **6** \| **1** \| **6** \| **1** \| **2** \| **9** \| **2** \| **23** \| **2** \| **11** \| **3** \| **0** \| **1** \| **0** \| **3** \| **4** \| **0** \| **2** \| **6** \| **0** \| \| **WR** \| ***Phlebiopsis gigantea*** \| **2** \| **3** \| **9** \| **19** \| **1** \| **5** \| **1** \| **4** \| **2*** \| **3** \| **9** \| **2** \| **23** \| **2** \| **12** \| **4** \| **1** \| **1** \| **0** \| **3** \| **10** \| **0** \| **2** \| **6** \| **0** \| |  |  |  |  |  |  |  |  |  |  |  |  |  |  |  |  |  |  |  |  |  |  |
| --- | --- | --- | --- | --- | --- | --- | --- | --- | --- | --- | --- | --- | --- | --- | --- | --- | --- | --- | --- | --- | --- | --- | --- | --- | --- | --- | --- | --- | --- | --- | --- | --- | --- | --- | --- | --- | --- | --- | --- | --- | --- | --- | --- | --- | --- | --- | --- | --- | --- | --- | --- | --- | --- | --- | --- | --- | --- | --- | --- | --- | --- | --- | --- | --- | --- | --- | --- | --- | --- | --- | --- | --- | --- | --- | --- | --- | --- | --- | --- | --- | --- | --- | --- | --- | --- | --- | --- | --- | --- | --- | --- | --- | --- | --- | --- | --- | --- | --- | --- | --- | --- | --- | --- | --- | --- | --- | --- | --- | --- | --- | --- | --- | --- | --- | --- | --- | --- | --- | --- | --- | --- | --- | --- | --- | --- | --- | --- | --- | --- | --- | --- | --- | --- | --- | --- | --- | --- | --- | --- | --- | --- | --- | --- | --- | --- | --- | --- | --- | --- | --- | --- | --- | --- | --- | --- | --- | --- | --- | --- | --- | --- | --- | --- | --- | --- | --- | --- | --- | --- | --- | --- | --- | --- | --- | --- | --- | --- | --- | --- | --- | --- | --- | --- | --- | --- | --- | --- | --- | --- | --- | --- | --- | --- | --- | --- | --- | --- | --- | --- | --- | --- | --- | --- | --- | --- | --- | --- | --- | --- | --- | --- | --- | --- | --- | --- | --- | --- | --- | --- | --- | --- | --- | --- | --- | --- | --- | --- | --- | --- | --- | --- | --- | --- | --- | --- | --- | --- | --- | --- | --- | --- | --- | --- | --- | --- | --- | --- | --- | --- | --- | --- | --- | --- | --- | --- | --- | --- | --- | --- | --- | --- | --- | --- | --- | --- | --- | --- | --- | --- | --- | --- | --- | --- | --- | --- | --- | --- | --- | --- | --- | --- | --- | --- | --- | --- | --- | --- | --- | --- | --- | --- | --- | --- | --- | --- | --- | --- | --- | --- | --- | --- | --- | --- | --- | --- | --- | --- | --- | --- | --- | --- | --- | --- | --- | --- | --- | --- | --- | --- | --- | --- | --- | --- | --- | --- | --- | --- | --- | --- | --- | --- | --- | --- | --- | --- | --- | --- | --- | --- | --- | --- | --- | --- | --- | --- | --- | --- | --- | --- | --- | --- | --- | --- | --- | --- | --- | --- | --- | --- | --- | --- | --- | --- | --- | --- | --- | --- | --- | --- | --- | --- | --- | --- | --- | --- | --- | --- | --- | --- | --- | --- | --- | --- | --- | --- | --- | --- | --- | --- | --- | --- | --- | --- | --- | --- | --- | --- | --- | --- | --- | --- | --- | --- | --- | --- | --- | --- | --- | --- | --- | --- | --- | --- | --- | --- | --- | --- | --- | --- | --- | --- | --- | --- | --- | --- | --- | --- | --- | --- | --- | --- | --- | --- | --- | --- | --- | --- | --- | --- | --- | --- | --- | --- | --- | --- | --- | --- | --- | --- | --- | --- | --- | --- | --- | --- | --- | --- | --- | --- | --- | --- | --- | --- | --- | --- | --- | --- | --- | --- | --- | --- | --- | --- | --- | --- | --- | --- | --- | --- | --- | --- | --- | --- | --- | --- | --- | --- | --- | --- | --- | --- | --- | --- | --- | --- | --- | --- | --- | --- | --- | --- | --- | --- | --- | --- | --- | --- | --- | --- | --- | --- | --- | --- | --- | --- | --- | --- | --- | --- | --- | --- | --- | --- | --- | --- | --- | --- | --- | --- | --- | --- | --- | --- | --- | --- | --- | --- | --- | --- | --- | --- | --- | --- | --- | --- | --- | --- | --- | --- | --- | --- | --- | --- | --- | --- | --- | --- | --- | --- | --- | --- | --- | --- | --- | --- | --- | --- | --- | --- | --- | --- | --- | --- | --- | --- | --- | --- | --- | --- | --- | --- | --- | --- | --- | --- | --- | --- | --- | --- | --- | --- | --- | --- | --- | --- | --- | --- | --- | --- | --- | --- | --- | --- | --- | --- | --- | --- | --- | --- | --- | --- | --- | --- | --- | --- | --- | --- | --- | --- | --- | --- | --- | --- | --- | --- | --- | --- | --- | --- | --- | --- | --- | --- | --- | --- | --- | --- | --- | --- | --- | --- | --- | --- | --- | --- | --- | --- | --- | --- | --- | --- | --- | --- | --- | --- | --- | --- | --- | --- | --- | --- | --- | --- | --- | --- | --- | --- | --- | --- | --- |
|  |  |  |  |  |  |  |  |  |  |  |  |  |  |  |  |  |  |  |  |  |  |  |

*Number excludes fragmented model. MP, Mycoparasite.

| **Table S7 continued. Glycoside hydrolase comparisons of brown-rot (BR) and white-rot (WR) fungi** | | | | | | | | | | | | | | | | | | | |
| --- | --- | --- | --- | --- | --- | --- | --- | --- | --- | --- | --- | --- | --- | --- | --- | --- | --- | --- | --- |
|  | **Glycoside hydrolase (GH) family** | | | | | | | | | | | | | | | | | | |
| **Species** | **35** | **36** | **37** | **38** | **43** | **44** | **45** | **47** | **51** | **52** | **53** | **54** | **55** | **61** | **63** | **71** | **72** | **74** | **75** |
| ***P. placenta*** | **1** | **0** | **3** | **1** | **1** | **0** | **0** | **5** | **1** | **0** | **1** | **0** | **3** | **2** | **1** | **2** | **2** | **0** | **0** |
| ***F. pinicola*** | **2** | **0** | **2** | **1** | **7** | **0** | **1** | **5** | **4** | **0** | **1** | **0** | **3** | **4** | **1** | **4** | **1** | **0** | **0** |
| ***W. cocos*** | **2** | **0** | **4** | **1** | **1** | **0** | **0** | **5** | **4** | **0** | **1** | **0** | **3** | **2** | **1** | **1** | **2** | **0** | **0** |
| ***G. trabeum*** | **2** | **0** | **2** | **1** | **6** | **0** | **1** | **4** | **4** | **0** | **2** | **0** | **2** | **4** | **1** | **6** | **1** | **1** | **0** |
| ***C. puteana*** | **2** | **0** | **4** | **1** | **6** | **0** | **1** | **9** | **3** | **0** | **1** | **0** | **5** | **10** | **1** | **6** | **1** | **0** | **0** |
| ***Dacryopinax*** | **1** | **1** | **2** | **1** | **5** | **0** | **1** | **4** | **2** | **0** | **1** | **0** | **2** | **0** | **1** | **5** | **1** | **0** | **0** |
| ***T. mesenterica*** | **0** | **1** | **2** | **1** | **0** | **0** | **0** | **4** | **0** | **0** | **0** | **0** | **0** | **0** | **0** | **3** | **1** | **0** | **0** |
| ***D. squalens*** | **3** | **0** | **3** | **1** | **7** | **1** | **1** | **5** | **2** | **0** | **1** | **0** | **2** | **15** | **1** | **4** | **1** | **1** | **0** |
| ***T. versicolor*** | **2** | **0** | **2** | **1** | **3** | **0** | **2** | **5** | **2** | **0** | **1** | **0** | **2** | **18** | **1** | **5** | **1** | **1** | **0** |
| ***F. mediterranea*** | **2** | **0** | **3** | **1** | **7** | **0** | **0** | **5** | **1** | **0** | **1** | **0** | **1** | **13** | **1** | **2** | **1** | **4** | **0** |
| ***A. delicata*** | **6** | **0** | **3** | **2** | **28** | **1** | **2** | **12** | **3** | **1** | **1** | **0** | **5** | **20** | **1** | **12** | **1** | **1** | **3** |
| ***P. strigosozonata*** | **4** | **0** | **2** | **1** | **7** | **1** | **1** | **5** | **3** | **0** | **2** | **2** | **3** | **14** | **1** | **5** | **1** | **2** | **0** |
| ***H. annosum*** | **4** | **0** | **2** | **1** | **4** | **0** | **2** | **6** | **1** | **0** | **1** | **0** | **1** | **10** | **1** | **3** | **1** | **1** | **0** |
| ***S. hirsutum*** | **7** | **0** | **2** | **1** | **12** | **1** | **1** | **6** | **3** | **0** | **2** | **0** | **2** | **16** | **1** | **8** | **2** | **2** | **1** |
| ***Ganoderma*** | **7** | **0** | **2** | **1** | **12** | **0** | **2** | **6** | **2** | **0** | **1** | **0** | **3** | **16** | **1** | **6** | **1** | **1** | **0** |
| ***B. adusta*** | **4** | **0** | **3** | **1** | **6** | **0** | **1** | **6** | **2** | **0** | **1** | **0** | **3** | **28** | **1** | **3** | **1** | **2** | **0** |
| ***P. brevispora*** | **4** | **0** | **3** | **1** | **2** | **2** | **3** | **9** | **1** | **0** | **1** | **0** | **4** | **12** | **1** | **4** | **1** | **1** | **0** |
| ***P. carnosa*** | **4** | **0** | **2** | **3** | **4** | **0** | **1** | **5** | **2** | **0** | **1** | **0** | **2** | **11** | **1** | **2** | **1** | **2** | **0** |
| ***C. subvermispora*** | **1** | **0** | **2** | **1** | **2** | **0** | **2** | **4** | **2** | **0** | **6** | **0** | **2** | **9** | **1** | **1** | **1** | **1** | **0** |
| ***P. chrysosporium*** | **3** | **0** | **2** | **1** | **4** | **0** | **2** | **6** | **2** | **0** | **1** | **0** | **2** | **13** | **1** | **3** | **1** | **4** | **0** |
| ***P. gigantea*** | **2** | **0** | **2** | **1** | **7** | **0** | **1** | **6** | **2** | **0** | **1** | **0** | **2** | **15** | **1** | **4** | **1** | **2** | **0** |

| **Table S7 continued. Glycoside hydrolase comparisons of brown-rot (BR) and white-rot (WR) fungi** | | | | | | | | | | | | | | | | | | |
| --- | --- | --- | --- | --- | --- | --- | --- | --- | --- | --- | --- | --- | --- | --- | --- | --- | --- | --- |
|  | **Glycoside hydrolase (GH) family** | | | | | | | | | | | | | | | | | |
| **Species** | **76** | **78** | **79** | **81** | **85** | **88** | **89** | **92** | **93** | **94** | **95** | **105** | **114** | **115** | **125** | **127** | **128** | **131** |
| ***P. placenta*** | **0** | **3** | **2** | **0** | **1** | **1** | **1** | **3** | **0** | **0** | **1** | **1** | **0** | **1** | **1** | **0** | **5** | **0** |
| ***F. pinicola*** | **0** | **3** | **3** | **0** | **1** | **1** | **2** | **5** | **0** | **0** | **1** | **3** | **0** | **1** | **1** |  |  |  |
| ***W. cocos*** | **0** | **3** | **3** | **0** | **1** | **1** | **1** | **3** | **0** | **0** | **1** | **0** | **0** | **2** | **1** |  |  |  |
| ***G. trabeum*** | **4** | **2** | **6** | **1** | **1** | **1** | **0** | **4** | **0** | **0** | **1** | **2** | **0** | **2** | **1** |  |  |  |
| ***C. puteana*** | **3** | **2** | **4** | **1** | **1** | **1** | **2** | **4** | **1** | **0** | **1** | **0** | **1** | **2** | **1** |  |  |  |
| ***Dacryopinax*** | **0** | **0** | **7** | **0** | **2** | **1** | **0** | **1** | **0** | **0** | **0** | **0** | **0** | **2** | **0** |  |  |  |
| ***T. mesenterica*** | **0** | **0** | **1** | **0** | **0** | **0** | **0** | **1** | **0** | **0** | **0** | **0** | **0** | **0** | **0** |  |  |  |
| ***D. squalens*** | **1** | **5** | **13** | **0** | **1** | **1** | **1** | **5** | **1** | **0** | **1** | **1** | **0** | **2** | **1** |  |  |  |
| ***T. versicolor*** | **0** | **3** | **11** | **0** | **1** | **1** | **1** | **4** | **0** | **0** | **1** | **1** | **0** | **2** | **1** |  |  |  |
| ***F. mediterranea*** | **0** | **2** | **7** | **0** | **1** | **2** | **0** | **4** | **0** | **0** | **2** | **1** | **0** | **3** | **1** |  |  |  |
| ***A. delicata*** | **1** | **4** | **13** | **5** | **1** | **2** | **1** | **7** | **1** | **1** | **1** | **3** | **4** | **2** | **1** |  |  |  |
| ***P. strigosozonata*** | **3** | **7** | **9** | **0** | **1** | **1** | **0** | **4** | **1** | **0** | **1** | **2** | **0** | **1** | **1** |  |  |  |
| ***H. annosum*** | **1** | **2** | **9** | **2** | **1** | **1** | **0** | **4** | **0** | **0** | **1** | **2** | **0** | **1** | **1** | **0** | **1** | **2** |
| ***S. hirsutum*** | **4** | **3** | **12** | **2** | **1** | **1** | **0** | **6** | **1** | **0** | **1** | **2** | **0** | **2** | **1** |  |  |  |
| ***Ganoderma*** | **2** | **4** | **12** | **0** | **1** | **1** | **2** | **5** | **2** | **0** | **2** | **1** | **0** | **3** | **1** | **0** | **7** |  |
| ***B. adusta*** | **0** | **2** | **9** | **0** | **1** | **1** | **1** | **3** | **0** | **0** | **1** | **1** | **0** | **2** | **1** | **0** | **5** |  |
| ***P. brevispora*** | **0** | **1** | **11** | **0** | **3** | **1** | **3** | **4** | **0** | **0** | **3** | **0** | **0** | **2** | **1** | **1** | **3** |  |
| ***P. carnosa*** | **0** | **1** | **11** | **0** | **1** | **1** | **1** | **4** | **0** | **0** | **1** | **0** | **0** | **1** |  |  |  |  |
| ***C. subvermispora*** | **0** | **1** | **8** | **0** | **1** | **1** | **2** | **3** | **0** | **0** | **1** | **0** | **0** | **2** | **1** | **1** | **3** | **1** |
| ***P. chrysosporium*** | **0** | **1** | **5** | **0** | **1** | **1** | **2** | **4** | **0** | **0** | **1** | **0** | **0** | **1** | **1** | **0** | **5** | **3** |
| ***P. gigantea*** | **0** | **1** | **7** | **0** | **1** | **1** | **2** | **4** | **0** | **0** | **0** | **0** | **0** | **1** | **1** | **0** | **6** | **2** |
