# Supplementary material for: Analysis of the Phlebiopsis gigantea Genome, Transcriptome and Secretome Provides Insight into Its Pioneer Colonization Strategies of Wood
Source: PLoS Genet. 2014 Dec 4;10(12):e1004759. doi: 10.1371/journal.pgen.1004759 (PMC4256170; doi:10.1371/journal.pgen.1004759)
Supplement: Table S8 — Carbohydrate esterase comparisons of brown-rot (BR) and white-rot (WR) fungi. (DOCX) [file pgen.1004759.s043.docx]

| **Table S8.** Carbohydrate esterase comparisons of brown-rot (BR) and white-rot (WR) fungi | | | | | | | | | | |
| --- | --- | --- | --- | --- | --- | --- | --- | --- | --- | --- |
|  |  | **Carbohydrate esterase (CE) families** | | | | | | | | |
| **Decay** | **Species** | **1** | **3** | **4** | **5** | **8** | **9** | **12** | **15** | **16** |
| **BR** | ***Postia placenta*** | **0** |  | **5** |  | **3** | **2** | **0** | **1** | **6** |
| **BR** | ***Fomitopsis pinicola*** | **1** | **0** | **3** | **0** | **2** | **7** | **0** | **1** | **8** |
| **BR** | ***Wolfiporia cocos*** | **1** | **0** | **4** | **0** | **1** | **1** | **0** | **1** | **6** |
| **BR** | ***Gloeophyllum trabeum*** | **3** | **0** | **5** | **0** | **2** | **1** | **0** | **1** | **7** |
| **BR** | ***Coniophora puteana*** | **1** | **0** | **9** | **1** | **2** | **1** | **0** | **0** | **7** |
| **BR** | ***Dacryopinax sp.*** | **1** | **0** | **11** | **0** | **3** | **0** | **0** | **1** | **3** |
| **MP** | ***Tremella mesenterica*** | **1** | **0** | **11** | **0** | **0** | **1** | **0** | **0** | **0** |
| **WR** | ***Dichomitus squalens*** | **1** | **0** | **3** | **0** | **3** | **1** | **2** | **2** | **13** |
| **WR** | ***Trametes versicolor*** | **4** | **0** | **3** | **0** | **2** | **1** | **0** | **2** | **8** |
| **WR** | ***Fomitiporia mediterranea*** | **1** | **0** | **4** | **0** | **3** | **1** | **2** | **1** | **6** |
| **WR** | ***Auricularia delicata*** | **5** | **3** | **11** | **3** | **2** | **1** | **2** | **6** | **12** |
| **WR** | ***Punctularia strigosozonata*** | **3** | **0** | **5** | **1** | **6** | **1** | **1** | **2** | **12** |
| **WR** | ***Heterobasidion annosum*** | **1** | **0** | **5** | **0** | **3** | **2*** | **2** | **1** | **5** |
| **WR** | ***Stereum hirsutum*** | **2** | **0** | **7** | **1** | **5** | **1** | **3** | **1** | **14** |
| **WR** | ***Ganoderma sp.*** | **2** | **0** | **4** | **0** | **3** | **1** | **1** | **2** | **16** |
| **WR** | ***Bjerkandera adusta*** | **1** | **0** | **3** | **0** | **2** | **1** | **1** | **2** | **15** |
| **WR** | ***Phlebia brevispora*** | **1** | **0** | **2** | **1** | **3** | **1** | **0** | **2** | **8** |
| **WR** | ***Phanerochaete_carnosa*** | **3** | **0** | **3** | **0** | **2** | **1** | **0** | **3** | **5** |
| **WR** | ***Ceriporiopsis subvermispora*** | **3** | **0** | **3** | **0** | **2** | **1** | **0** | **2** | **5** |
| **WR** | ***Phanerochaete chrysosporium*** | **5** | **0** | **3** | **0** | **2** | **1** | **0** | **2** | **2** |
| **WR** | ***Phlebiopsis gigantea*** | **2** | **0** | **3** | **0** | **3*** | **1** | **1** | **1** | **6** |

MP, Mycoparasite.

*Number excludes fragmented models
