# Supplementary material for: Analysis of the Phlebiopsis gigantea Genome, Transcriptome and Secretome Provides Insight into Its Pioneer Colonization Strategies of Wood
Source: PLoS Genet. 2014 Dec 4;10(12):e1004759. doi: 10.1371/journal.pgen.1004759 (PMC4256170; doi:10.1371/journal.pgen.1004759)
Supplement: Table S9 — Polysaccharide lyase comparisons of brown-rot (BR) and white-rot (WR) fungi. (DOCX) [file pgen.1004759.s044.docx]

| **Table S9.** Polysaccharide lyase comparisons of brown-rot (BR) and white-rot (WR) fungi | | | | | | | |
| --- | --- | --- | --- | --- | --- | --- | --- |
|  |  | **Polysaccharide lyase (PL) families** | | | | | |
| **Decay** | **Species** | **1** | **3** | **4** | **7** | **8** | **14** |
| **BR** | ***Postia placenta*** |  |  |  |  |  | **8** |
| **BR** | ***Fomitopsis pinicola*** | **0** | **0** | **0** | **0** | **0** | **3** |
| **BR** | ***Wolfiporia cocos*** | **0** | **0** | **0** | **0** | **0** | **2** |
| **BR** | ***Gloeophyllum trabeum*** | **0** | **0** | **2** | **0** | **1** | **6** |
| **BR** | ***Coniophora puteana*** | **0** | **0** | **0** | **0** | **0** | **3** |
| **BR** | ***Dacryopinax sp.*** | **0** | **0** | **0** | **0** | **0** | **3** |
| **MP** | ***Tremella mesenterica*** | **0** | **0** | **0** | **0** | **0** | **2** |
| **WR** | ***Dichomitus squalens*** | **0** | **0** | **1** | **0** | **3** | **7** |
| **WR** | ***Trametes versicolor*** | **0** | **0** | **1** | **0** | **2** | **6** |
| **WR** | ***Fomitiporia mediterranea*** | **2** | **0** | **0** | **0** | **0** | **4** |
| **WR** | ***Auricularia delicata*** | **2** | **1** | **1** | **1** | **2** | **13** |
| **WR** | ***Punctularia strigosozonata*** | **4** | **0** | **3** | **0** | **1** | **4** |
| **WR** | ***Heterobasidion annosum*** | **2** | **0** | **1** | **0** | **2** | **4** |
| **WR** | ***Stereum hirsutum*** | **4** | **0** | **3** | **0** | **2** | **5** |
| **WR** | ***Ganoderma sp.*** | **0** | **0** | **0** | **0** | **3** | **6** |
| **WR** | ***Bjerkandera adusta*** | **1** | **2** | **1** | **0** | **1** | **5** |
| **WR** | ***Phlebia brevispora*** | **0** | **0** | **1** | **0** | **1** | **5** |
| **WR** | ***Phanerochaete carnosa*** | **0** | **0** | **0** | **0** | **1** | **7** |
| **WR** | ***Ceriporiopsis subvermispora*** | **0** | **0** | **0** | **0** | **2** | **4** |
| **WR** | ***Phanerochaete chrysosporium*** | **0** | **0** | **0** | **0** | **1** | **3** |
| **WR** | ***Phlebiopsis gigantea*** | **0** | **0** | **0** | **0** | **1** | **5** |
