# Supplementary material for: Analysis of the Phlebiopsis gigantea Genome, Transcriptome and Secretome Provides Insight into Its Pioneer Colonization Strategies of Wood
Source: PLoS Genet. 2014 Dec 4;10(12):e1004759. doi: 10.1371/journal.pgen.1004759 (PMC4256170; doi:10.1371/journal.pgen.1004759)
Supplement: Table S10 — Protein features and regulation of lytic polysaccharide monooxygenases (LPMO) in P. gigantea. (DOCX) [file pgen.1004759.s045.docx]

| **Table S10**: Protein features and regulation of lytic polysaccharide monooxygenases (LPMO) in *Phlebiopsis gigantea* | | | | | | |
| --- | --- | --- | --- | --- | --- | --- |
| Protein ID | Predicted  Signal peptidase cleavage site | Detected Domains^1^ | Peptide Detected | Significant Regulation^2^ | | |
|  |  |  |  | ELP/Glu | NELP/Glu | ELP/NELP |
| 227588 | TSA-HT | LPMO | yes | yes | yes | no |
| 20470 | VAA-HY | LPMO | no | no | no | no |
| 72251 | VTA-HT | LPMO | no | no | no | no |
| 227560 | SSA-HT | LPMO/CBM1 | yes | yes | yes | no |
| 27670 | VVA-HG | LPMO/CBM1 | no | no | no | no |
| 35240 | VSA-HG | LPMO | no | no | no | no |
| 37310 | VAG-HG | LPMO | yes | no^3^ | no^3^ | no^3^ |
| 118444 | ALA-HY | LPMO | yes | no | no | no |
| 112944 | VAA-HG | LPMO | no | no | no | yes - down |
| 18264 | VLA-HG | LPMO/CBM1 | no | yes | yes | no |
| 112087 | AAA-HY | LPMO | no | no | no | no |
| 79150 | AHA-TF^4^ | LPMO/CBM1tr^5^ | no | yes (p=0.021)^6^ | yes | no |
| 79143 | none | trLPMO | no | no | no | no |
| 125213 | VEG-HG | LPMOtr | no | no | yes | yes - down |
| 64774 | none | trLPMO | no | no | no | Yes - down |

^2^ Significant regulation (p≤0.01) is assumed to be ‘up’ unless otherwise stated.

^3^ Transcript is abundant on all substrates tested (RPKM values >1200).

^4^ The predicted Signal-P cleavage site would remove the family conserved N-terminal histidine (underlined).

^1^ Domains appended with a ‘tr’ designation indicates a truncation and its relative position.

^5^ Sequence alignments indicate that C-terminal CBM1 is truncated.

^6^ Although the p-value was slightly higher than the 0.01 threshold, a 26-fold increase was observed at p=0.02.
