# Supplementary material for: Analysis of the Phlebiopsis gigantea Genome, Transcriptome and Secretome Provides Insight into Its Pioneer Colonization Strategies of Wood
Source: PLoS Genet. 2014 Dec 4;10(12):e1004759. doi: 10.1371/journal.pgen.1004759 (PMC4256170; doi:10.1371/journal.pgen.1004759)
Supplement: Table S11 — Properties of the transcription factors for which binding sites have been detected in CAZymes that are significantly regulated during growth on ground pine wood. (DOCX) [file pgen.1004759.s046.docx]

| **Table S11** Properties of the transcription factors for which binding sites have been detected in CAZymes that are significantly regulated during growth on ground pine |
| --- |
| Ash1p Zinc-finger inhibitor of HO transcription; mRNA is localized and translated in the distal tip of anaphase cells, resulting in accumulation of Ash1p in daughter cell nuclei and inhibition of HO expression; potential Cdc28p substrate |
| Crz1p Transcription factor that activates transcription of genes involved in stress response; nuclear localization is positively regulated by calcineurin-mediated dephosphorylation |
| Fkh1p Forkhead family transcription factor with a minor role in the expression of G2/M phase genes; negatively regulates transcriptional elongation; positive role in chromatin silencing at HML and HMR; regulates donor preference during switching |
| Gcr1p Transcriptional activator of genes involved in glycolysis; DNA-binding protein that interacts and functions with the transcriptional activator Gcr2p |
| Gis1p JmjC domain-containing histone demethylase; transcription factor involved in the expression of genes during nutrient limitation; also involved in the negative regulation of DPP1 and PHR1 |
| Hac1p bZIP transcription factor (ATF/CREB1 homolog) that regulates the unfolded protein response, via UPRE binding, and membrane biogenesis; ER stress-induced splicing pathway utilizing Ire1p, Trl1p and Ada5p facilitates efficient Hac1p synthesis |
| Mot3p Nuclear transcription factor with two Cys2-His2 zinc fingers; involved in repression of a subset of hypoxic genes by Rox1p, repression of several DAN/TIR genes during aerobic growth, and repression of ergosterol biosynthetic genes |
| Msn2/4p/ general activator of transcription, contribution to osmotic tress (=SEB1) |
| Xbp1 Transcriptional repressor that binds to promoter sequences of the cyclin genes, CYS3, and SMF2; expression is induced by stress or starvation during mitosis, and late in meiosis; member of the Swi4p/Mbp1p family; potential Cdc28p substrate |
| Gsm1p Putative zinc cluster protein of unknown function; proposed to be involved in the regulation of energy metabolism, based on patterns of expression and sequence analysis |
