# Supplementary material for: Analysis of the Phlebiopsis gigantea Genome, Transcriptome and Secretome Provides Insight into Its Pioneer Colonization Strategies of Wood
Source: PLoS Genet. 2014 Dec 4;10(12):e1004759. doi: 10.1371/journal.pgen.1004759 (PMC4256170; doi:10.1371/journal.pgen.1004759)
Supplement: Table S12 — Transcript levels of potential regulators in P. gigantea cultures. (DOCX) [file pgen.1004759.s047.docx]

| **Table S12**. Transcript levels of potential regulators in *P. gigantea* cultures | | | | | | | | | | | | |
| --- | --- | --- | --- | --- | --- | --- | --- | --- | --- | --- | --- | --- |
|  |  | Phlgi |  | RPKM | | | NELP/Glu | NELP/Glu | NELP/ELP | NELP/ELP | ELP/Glu | ELP/Glu |
| Factor | Consensus site | Pro* | e-value | NELP | ELP | Glu | Prob | Ratio | Prob | Ratio | Prob | Ratio |
| Gsm1p | CGGNNNNNNNNCGG | [76307](http://genome.jgi-psf.org/cgi-bin/dispGeneModel?db=Phlgi1&id=76307) | 1.7E-27 | 57.90 | 55.34 | 26.08 | 0.03 | 2.22 | 0.73 | 1.05 | 0.03 | 2.12 |
| Xbp1p | CTCGA | [21919](http://genome.jgi-psf.org/cgi-bin/dispGeneModel?db=Phlgi1&id=21919) | 8.6E-13 | 8.47 | 4.64 | 19.0 | 0.26 | 0.45 | 0.11 | 1.83 | 0.07 | 0.24 |
| Mot3p | TMGGAA | [46901](http://genome.jgi-psf.org/cgi-bin/dispGeneModel?db=Phlgi1&id=46901) | 5.7E-8 | 0.78 | ------ | ------ |  |  |  |  |  |  |
| Gis1p | AGGGG | [130940](http://genome.jgi-psf.org/cgi-bin/dispGeneModel?db=Phlgi1&id=130940) | 3.2E-42 | 47.90 | 42.11 | 52.98 | 0.78 | 0.90 | 0.41 | 1.14 | 0.53 | 0.79 |
| Gcr1 | CWTCC | no hits |  |  |  |  |  |  |  |  |  |  |
| Fkh1p | RYMAAYA | [89212](http://genome.jgi-psf.org/cgi-bin/dispGeneModel?db=Phlgi1&id=89212) | 7.8E-22 | 71.77 | 80.19 | 41.96 | 0.09 | 1.71 | 0.35 | 0.90 | 0.06 | 1.91 |
| Crz1p | GNGGCKCA | [20014](http://genome.jgi-psf.org/cgi-bin/dispGeneModel?db=Phlgi1&id=20014) | 2.6E-23 | 92.42 | 68.94 | 65.07 | 0.31 | 1.42 | 0.14 | 1.34 | 0.87 | 1.06 |
| Ash1p | YTGAT | no hits |  |  |  |  |  |  |  |  |  |  |
| Msn2/4 (=SEB1) | CCCCT | 79048 | 2.1E-11 | 347.39 | 299.68 | 28.28 | 0.02 | 12.29 | 0.46 | 1.16 | 0.02 | 10.60 |
| Hac1 | CCAGC | no hits |  |  |  |  |  |  |  |  |  |  |

Using yeast protein as query.
