# Supplementary material for: Analysis of the Phlebiopsis gigantea Genome, Transcriptome and Secretome Provides Insight into Its Pioneer Colonization Strategies of Wood
Source: PLoS Genet. 2014 Dec 4;10(12):e1004759. doi: 10.1371/journal.pgen.1004759 (PMC4256170; doi:10.1371/journal.pgen.1004759)
Supplement: Table S13 — Overview of the P. gigantea P450ome and comparison with P. chrysosporium and P. carnosa. (DOCX) [file pgen.1004759.s048.docx]

| **Table S13**. Overview of the *P. gigantea* P450ome and comparison with *P. chrysosporium and P. carnosa*. | | | |
| --- | --- | --- | --- |
| Species | *P. gigantea* | *P. chrysosporium* | *P. carnosa* |
| Authentic P450s: |  |  |  |
| Clans | 11 | 10 | 11 |
| Families | 34 | 32 | 36 |
| Subfamilies | 61 | 70 | 74 |
| Member P450s | 127 | 149 | 266 |
| Tentative P450s/Pseudogenes | 9 | 10 | 19 |
