# Supplementary material for: Analysis of the Phlebiopsis gigantea Genome, Transcriptome and Secretome Provides Insight into Its Pioneer Colonization Strategies of Wood
Source: PLoS Genet. 2014 Dec 4;10(12):e1004759. doi: 10.1371/journal.pgen.1004759 (PMC4256170; doi:10.1371/journal.pgen.1004759)
Supplement: Table S14 — Clan-, family-, and subfamily- level classification of the P450ome of P. gigantea and comparison with P. chrysosporium and P. carnosa. (DOCX) [file pgen.1004759.s049.docx]

| **Table S14.** Clan-, family-, and subfamily- level classification of the P450ome of *P. gigantea* and comparison with *P. chrysosporium* and *P. carnosa* | | | | | | | | |
| --- | --- | --- | --- | --- | --- | --- | --- | --- |
| **P450 Clan** | **P450 Family** | **P450 Subfamily** | ***P. gigantea*** | | ***P. chrysosporium*** | | ***P. carnosa*** | |
|  |  |  | **Member P450s** | | **Member P450s** | | **Member P450s** | |
|  |  |  | **Subfamily-wise** | **Clan-wise** | **Subfamily-wise** | **Clan-wise** | **Subfamily-wise** | **Clan-wise** |
| **CYP51** | CYP51 | F | 1 | 5 | 1 | 5 | **2** | **5** |
|  | CYP5140 | A | 1 |  | 1 |  |  |  |
|  | CYP5157 | A | 1 |  | 1 |  | **1** |  |
|  | CYP5156 | A | 2 |  | 2 |  | **1** |  |
|  | CYP5025 | NS |  |  |  |  | **1** |  |
| **CYP52** | CYP63 | A | 3 | 21 | 4 | 18 | **7** | **32** |
|  |  | B | 1 |  | 1 |  | **1** |  |
|  |  | C |  |  | 2 |  | **1** |  |
|  | CYP5137 | A |  |  | 2 |  | **4** |  |
|  | CYP5138 | A | 1 |  | 1 |  |  |  |
|  | CYP5139 | A | 3 |  | 1 |  |  |  |
|  | CYP5141 | A |  |  | 4 |  | 6 |  |
|  |  | B |  |  | 1 |  |  |  |
|  |  | C |  |  | 1 |  | 1 |  |
|  |  | D |  |  | 1 |  | 1 |  |
|  |  | NS |  |  |  |  | 1 |  |
|  | CYP5142 | A |  |  | 3 |  | 1 |  |
|  |  | B |  |  | 1 |  | 2 |  |
|  |  | C |  |  | 1 |  | 4 |  |
|  |  | D |  |  | 1 |  |  |  |
|  |  | E |  |  | 1 |  | 1 |  |
|  | CYP5150 | A | 7 |  | 5 |  |  |  |
|  |  | B | 3 |  | 1 |  |  |  |
|  |  | C |  |  | 1 |  |  |  |
|  |  | NS | 1 |  |  |  |  |  |
|  | CYP5151 | A | 1 |  | 1 |  | **1** |  |
|  |  | B |  |  |  |  | **1** |  |
|  | CYP5155 | A | 1 |  | 1 |  |  |  |
| **CYP53** | CYP53 | C | 4 | 12 | 1 | 10 | **6** | **10** |
|  |  | NS | 2 |  |  |  | **1** |  |
|  | CYP5140 |  |  |  | A | 1 |  | 1 |
|  | CYP5142 | A | 1 |  | 3 |  |  |  |
|  |  | B |  |  | 1 |  |  |  |
|  |  | C | 3 |  | 1 |  |  |  |
|  |  | D |  |  | 1 |  |  |  |
|  |  | E |  |  | 1 |  |  |  |
|  | CYP5143 | A | 1 |  | 2 |  | **2** |  |
|  |  | NS | 1 |  |  |  |  |  |
| **CYP54** | CYP512 | B |  |  | 5 | 14 | 2 | 27 |
|  |  | C |  |  | 2 |  | 9 |  |
|  |  | D |  |  | 1 |  |  |  |
|  |  | E |  |  | 1 |  | 1 |  |
|  |  | F |  |  | 1 |  | 2 |  |
|  |  | G |  |  | 2 |  | 7 |  |
|  |  | H |  |  | 1 |  | 5 |  |
|  |  | J |  |  | 1 |  |  |  |
|  |  | NS |  |  |  |  | 1 |  |
| **CYP61** | CYP61 | A | 1 | 1 | 1 | 1 | **1** | **1** |
| **CYP64** | CYP502 | A | 1 | 50 |  | 61 |  | **124** |
|  |  | B | 1 |  | 1 |  | **1** |  |
|  | CYP620 | NS |  |  |  |  | **1** |  |
|  | CYP5037 | A | 1 |  | 1 |  | **1** |  |
|  |  | B | 5 |  | 3 |  | **5** |  |
|  |  | C |  |  | 1 |  | **1** |  |
|  |  | NS | 1 |  |  |  | **1** |  |
|  | CYP5093 | NS | 1 |  |  |  |  |  |
|  | CYP5144 | A | 4 |  | 15 |  | **19** |  |
|  |  | B |  |  | 1 |  | **7** |  |
|  |  | C | 8 |  | 8 |  | **16** |  |
|  |  | D | 2 |  | 5 |  | **3** |  |
|  |  | E | 2 |  | 1 |  | **1** |  |
|  |  | F | 1 |  | 1 |  | **5** |  |
|  |  | G | 2 |  | 1 |  | **7** |  |
|  |  | H | 4 |  | 1 |  | **12** |  |
|  |  | J |  |  | 1 |  | **1** |  |
|  |  | NS | 1 |  |  |  |  |  |
|  | CYP5145 | A | 3 |  | 3 |  | **2** |  |
|  |  | NS | 1 |  |  |  |  |  |
|  | CYP5146 | A | 1 |  | 4 |  | **5** |  |
|  |  | B | 1 |  | 1 |  | **1** |  |
|  |  | C | 1 |  | 1 |  | **9** |  |
|  | CYP5147 | A | 1 |  | 4 |  | **6** |  |
|  |  | B |  |  | 1 |  | **1** |  |
|  |  | C | 1 |  | 1 |  |  |  |
|  | CYP5148 | A | 1 |  | 2 |  | **3** |  |
|  |  | B | 2 |  |  |  | **4** |  |
|  | CYP5149 | A |  |  | 1 |  | **3** |  |
|  | CYP5152 | A | 2 |  | 2 |  | **4** |  |
|  | CYP5158 | A | 2 |  | 1 |  | **5** |  |
| **CYP67** | CYP5035 | A | 1 | 8 | 7 | 18 | **6** | **22** |
|  |  | B |  |  | 3 |  | **3** |  |
|  |  | C | 1 |  | 1 |  | **5** |  |
|  |  | D | 1 |  | 1 |  |  |  |
|  |  | E |  |  | 1 |  |  |  |
|  | CYP5036 | A | 2 |  | 3 |  | **3** |  |
|  |  | B |  |  | 1 |  |  |  |
|  |  | C | 3 |  | 1 |  | **5** |  |
| **CYP503** | CYP512 | B | 3 | 6 | 5 | 14 |  |  |
|  |  | C |  |  | 2 |  |  |  |
|  |  | D |  |  | 1 |  |  |  |
|  |  | E |  |  | 1 |  |  |  |
|  |  | F |  |  | 1 |  |  |  |
|  |  | G | 3 |  | 2 |  |  |  |
|  |  | H |  |  | 1 |  |  |  |
|  |  | J |  |  | 1 |  |  |  |
| **CYP505** | CYP505 | D | 4 | 4 | 7 | 7 | **4** | **4** |
| **CYP534** | CYP5141 | A | 6 | 10 | 4 | 18 |  | **29** |
|  |  | B |  |  | 1 |  |  |  |
|  |  | C | 1 |  | 1 |  |  |  |
|  |  | D |  |  | 1 |  |  |  |
|  | CYP5138 | A |  |  | 1 | 11 | 2 | 29 |
|  | CYP5139 | A |  |  | 1 |  | 11 |  |
|  | CYP5150 | A |  |  | 5 |  | 6 |  |
|  |  | B |  |  | 1 |  | 4 |  |
|  |  | C |  |  | 1 |  |  |  |
|  | CYP5153 | A |  |  |  |  | 1 |  |
|  | CYP5154 | A | 2 |  | 1 |  | **3** |  |
|  |  | NS | 1 |  |  |  | **1** |  |
|  | CYP5155 | A |  |  | 1 |  | **1** |  |
| **CYP547** | CYP5136 | A | 5 | 7 | 5 | 7 | **6** | **8** |
|  | NS |  |  |  |  |  | **2** |  |
|  | CYP5137 | A |  |  | 2 |  |  |  |
|  | CYP509 | NS | 2 |  |  |  |  |  |
| **UN** | CYP6001 | NS | 2 | 3 |  |  | **3** | **4** |
|  |  | C |  |  |  |  | **1** |  |
|  | CYP5108 | NS | 1 |  |  |  |  |  |
| **Total P450s** |  |  |  | **127** |  | **149** |  | **266** |
| **Tentative/Pseudo** |  |  |  | **9** |  | **10** |  | **19** |
| **P450 genes** |  |  |  |  |  |  |  |  |
| ^1^Abbreviations: NS, New subfamily; UN, Un-named. | | | | | | | | |
|  | New subfamily, not found in either *P. carnosa* or *P. chrysosporium.* | | | | | | | |
|  | Subfamily that is absent in *P. chrysosporium*, but present in *P. gigantea* and *P. carnosa.* | | | | | | | |
