# Supplementary material for: Analysis of the Phlebiopsis gigantea Genome, Transcriptome and Secretome Provides Insight into Its Pioneer Colonization Strategies of Wood
Source: PLoS Genet. 2014 Dec 4;10(12):e1004759. doi: 10.1371/journal.pgen.1004759 (PMC4256170; doi:10.1371/journal.pgen.1004759)
Supplement: Table S15 — Expression profile of P. gigantea P450ome. (DOCX) [file pgen.1004759.s050.docx]

| **Table S15**. Expression profile of *P. gigantea* P450ome. | | | | | |
| --- | --- | --- | --- | --- | --- |
| **Protein ID** | **CYP name** | **Putative function^1^** | **Regulation** | | |
|  |  |  | NELP / Glu | ELP / Glu | NELP / ELP |
| 120295 | CYP5156NS | not known (orphan) | 1.000 up | 2.199 down | 2.201 down |
| 37333 | CYP6001NS | oxylipin biosynthesis (oxidation of mono- and polyunsaturated C16/C18 fatty acids) | 2.008 up | 2.046 down | 4.109 down |
| 114194 | CYP5037B | deethylation of 7-ethoxycoumarin | 1.932 down | 1.221 up | 2.361 up |
| 121846 | CYP5144H | triterpenoid biosynthesis | 1.272 down | 1.108 up | 1.411 up |
| 20514 | CYP512B | triterpenoid biosynthesis | 27.201 up | 2.209 up | 12.308 down |
| 68457 | CYP5035A | flavone hydroxylation | 2.898 down | 5.191 down | 1.791 down |
| 25626 | CYP5144C | triterpenoid biosynthesis | 1.966 up | 3.713 up | 1.888 up |
| 119094 | CYP5148A | not known (orphan) | 6.171 up | 1.062 up | 5.806 down |
| 19184 | CYP5136A | xenobiotic compounds oxidation (polycyclic aromatic hydrocarbons and alkylphenols) | 1.069 up | 4.562 down | 4.879 down |
| 62450 | CYP5156A | not known (orphan) | 8.476 up | 6.908 up | 1.227 down |
| 131188 | CYP5154NS | not known (orphan) | 2.382 up | 2.706 up | 1.135 up |
| 20511 | CYP5150A | xenobiotic compounds oxidation (hydroxylation of 4-propylbenzoic acid) | 2.285 up | 2.548 down | 5.823 down |
| 119938 | CYP5139A | xenobiotic compounds oxidation (naphthalene, 7-ethoxycoumarin & naproxen) | 6.363 up | 5.815 up | 1.094 down |
| 89373 | CYP5150B | xenobiotic compounds oxidation (hydroxylation of 4-propylbenzoic acid) | 11.034 up | 1.630 up | 6.769 down |
| 35739 | CYP5154A | not known (orphan) | 1.891 down | 1.474 down | 1.282 up |
| 122687 | CYP5150A | xenobiotic compounds oxidation (hydroxylation of 4-propylbenzoic acid) | 10.608 up | 2.286 up | 4.640 down |
| 75338 | CYP53C | benzoate and its derivatives degradation/detoxification | 5.810 up | 1.050 down | 6.103 down |
| 98480 | CYP505D | *ω*-1 to *ω*-3 carbon hydroxylation of fatty acids | 1.054 up | 1.617 up | 1.533 up |
| 121631 | CYP5147A | steroid hydroxylation (testosterone) | 2.942 down | 4.549 down | 1.546 down |
| 62333 | CYP5144C | triterpenoid biosynthesis & polycyclic aromatic hydrocarbons oxidation | 1.605 up | 2.152 up | 1.340 up |
| 374250 | CYP5146B | not known (orphan) | 1.052 down | 2.000 up | 2.104 up |
| 101881 | CYP6001NS | oxylipin biosynthesis (oxidation of mono- and polyunsaturated C16/C18 fatty acids) | 1.085 down | 3.885 down | 3.580 down |
| 75427 | CYP5093NS | not known (orphan) | 1.614 down | 1.426 down | 1.131 up |
| 120534 | CYP512G | triterpenoid biosynthesis | 1.329 up | 1.622 up | 1.220 up |

^1^Putative function for the *P. gigantea* P450s were predicted based on functionally characterized homologs in other basidiomycetes.
