# Supplementary material for: Analysis of the Phlebiopsis gigantea Genome, Transcriptome and Secretome Provides Insight into Its Pioneer Colonization Strategies of Wood
Source: PLoS Genet. 2014 Dec 4;10(12):e1004759. doi: 10.1371/journal.pgen.1004759 (PMC4256170; doi:10.1371/journal.pgen.1004759)
Supplement: Table S16 — Products of annotated putative secondary metabolite genes in the P. gigantea genome. (DOCX) [file pgen.1004759.s051.docx]

| **Table 16.** Products of annotated putative secondary metabolite genes in the *P. gigantea* genome. | | | | | |
| --- | --- | --- | --- | --- | --- |
| **Protein** | **Protein ID** | **Protein length (aa)** | **Domain organization**  **(for PKS and NRPS)^1^** | **Preliminary gene designation** | **Remarks** |
| **polyketide synthase** |  |  |  |  |  |
|  | 36582 | n.d. | SAT-KS-AT-PT-ACP-ACP-TE | pks1 | reading frame disrupted by sequence gaps |
|  |  |  |  |  |  |
| **nonribosomal peptide synthetase-like proteins** |  |  |  |  |  |
|  | 66945 | 1424 | A-T-R | lys2 | putative α-aminoadipate reductase |
|  | 35145 | 1027 | A-T-R | nps1 |  |
|  |  |  |  |  |  |
| **terpenoid synthases** |  |  |  |  |  |
|  | 126738 | 338 |  | tes1 |  |
|  | 114823 | 335 |  | tes2 |  |
|  | 359064 | 337 |  | tes3 |  |
|  | 367715 | 352 |  | tes4 |  |
|  |  |  |  |  |  |
| **terpene cyclase** |  |  |  |  |  |
|  | 87649 | 739 |  | tcy1 | putative squalene synthase |
|  |  |  |  |  |  |
| **halogenase** |  |  |  |  |  |
|  | 534844 | 662 |  | hal1 |  |
|  | 30804 | 522 |  | hal2 |  |

^1^ Abbreviations for enzyme domains are: SAT: starter unit acyltransferases, KS: keto synthase, AT: acyl transferase, PT: product template, ACP: acyl carrier protein, TE: thioesterase, A: adenylation domain, T: thiolation domain (=peptidyl carrier protein), R: reductase. The gene designations have been assigned based on sequence homology.
