# Supplementary material for: Analysis of the Phlebiopsis gigantea Genome, Transcriptome and Secretome Provides Insight into Its Pioneer Colonization Strategies of Wood
Source: PLoS Genet. 2014 Dec 4;10(12):e1004759. doi: 10.1371/journal.pgen.1004759 (PMC4256170; doi:10.1371/journal.pgen.1004759)
Supplement: Table S17 — P. gigantea hydrophobin models. (DOCX) [file pgen.1004759.s052.docx]

| **Table S17.** *P. gigantea* hydrophobin models | | |  | | | |
| --- | --- | --- | --- | --- | --- | --- |
| **Existing model** | | | **Suggested model / changes** | | | |
| Model name | Protein ID | Location | Model name | Protein ID | Location | Remarks |
| estExt_Genemark1.C_460029 | 127149 | scaffold_46:7620876717(-) |  |  |  |  |
| fgenesh1_kg.346_#_1_#_Locus1003v1rpkm146.58 | 20660 | scaffold364:6264-6986 (+) |  |  |  | Based on homology with other basidiomycetes, the protein sequence is about 4-5 amino acids longer than orthologous sequences from other basidiomycetes. However, by shifting exon-intron boundary, the protein could be made 2 aa shorter |
| estExt_Genewise1Plus.C_460063 | 104620 | scaffold 46:77858-78299 (-) |  |  |  | The protein sequence is relatively longer than the orthologous sequences from other basidiomycetes. However, by shifting exon-intron boundary, the protein could be made 2 aa shorter: |
| MIX20949_136_97 | 509314 | scaffold 46:64398-64941 (-) | - | - | - | The protein appeared to have a relatively larger size in *H. annosum*, the first exon of the coding sequence was relatively long with two short exons at the second and third positions. In *C. subvermispora*, most of the N -terminal (5’) and C-terminal parts of the coding sequence of the protein (3’) were untranslated, although this did not significantly affect the size of the gene product. |
| gm1.922_g | 114166 | scaffold 6:114697-1151271 (+) |  |  |  | This protein has only 6 out of 8 conserved Cys residues. The coding region of the gene has 3 exons in all the tested homologues; however the gene structure appeared to be more similar with the homologue from *P. chrysosporum*. In *C. subvermispora* however*,* the first exon was relatively large in comparison with the other two homologues. This structural variation did not affect the size of the gene in the abovementioned species. |
| e_gw1.299.4.1 | 80088 | scaffold 299:4557-5695 (-) | uku_1_e_gw1.299.4.1 | 534803 | scaffold_299:5271-5695 | Model 80088 lacks a stop codon in its coding sequence, although model 534803 has only 6 out of 8 conserved Cys residues |
| gw1.30.9.1 | 39115 | Scaffold 30:47407-47800 (-) |  |  |  | Based on comparison with related basidiomycetes such as *P. chrysosporum* and *S. lacrimans*, a part of the protein was excluded in the existing model. This missing part truncated the protein length, presenting the coding sequence without a start codon. |
| estExt_Genemark1.C_80016 | 124694 | scaffold_8:30174-30968 | - | - | - | In the existing model, the protein sequence appears to have fused with some parts of another protein thereby making the N-terminal part longer than the original length. The following corrected sequence is suggested: MFSRVSVVLFYAFFAFALLAAATPAPALDNAKRWATPTTPATCNTGSIQCCQGVQSASLASSGLILGLLGIVLSTLDVLLGLQCSPIQIVGIGSGDGCEANVVCCENNSVGGLISIGCIPIIL. |
| fgenesh1_kg.6_#_15_#_Locus8475v1rpkm20.75 | 17842 | scaffold_6:117052-117473 | estExt_Genemark1.C_60039 | 124519 | scaffold_6:117000-117473 | The protein is relatively shorter than the hydrophobins from the closest ortholog, *P. chrysosporium*. In addition, the protein has only 6 out of 8 conserved Cys residues. An alternative start codon for this protein is suggested: MFSRLTAFSVLALPLFAAATPAMVARNDQPTSPTTACCDSTESANSAVGAALLGLLGIDLSDLNVLLGLTCSPISVVGVGSGTECSGTTVSCTNGVVGGIGIGCVPVSL |
| gw1.59.69.1 | 53256 | [scaffold_59:113710-114079](http://genome.jgi.doe.gov/cgi-bin/browserLoad?db=Phlgi1&position=scaffold_59:113710-114079) | gm1.4845_g | 118089 | scaffold_59:113707-114226 | A large portion of the N-terminal is missing in 53256 as is the last codon of the gene. The nucleotide sequence is relatively short when compared with the closest ortholog from *Serpula lacrymans*. |
| e_gw1.46.69.1 | 69703 | scaffold_46:67166-67626 | - | - | - |  |
| CE139609_236 | 270989 | [scaffold_207:20554-21674](http://genome.jgi.doe.gov/cgi-bin/browserLoad?db=Phlgi1&position=scaffold_207:20554-21674) | - | - | - | The existing model has only 6 out of 8 cysteine residues. |
| e_gw1.407.7.1 | 80816 | [scaffold_407:5014-5352](http://genome.jgi.doe.gov/cgi-bin/browserLoad?db=Phlgi1&position=scaffold_407:5014-5352) | gm1.10274 | scaffold_407:4908-5352 | 123518 | In the existing model, the protein sequence lacks the N-terminus with the start codon of the coding sequence conspicuously missing. In the closest ortholog. *P. chrysosporium*, the gene has 2 exons with the first exon relatively longer than the second exon. From comparison with the closest basidiomycete, *P. chrysosporium*, none of the available models seems to represent the true configuration of the protein. |
| gw1.407.4.1 | 39999 | [scaffold_407:2841-3232](http://genome.jgi.doe.gov/cgi-bin/browserLoad?db=Phlgi1&position=scaffold_407:2841-3232) |  |  |  | The coding sequence of the existing model has 4 exons with the start and stop codons missing. Based on comparison with the protein sequence from the closest ortholog (*P. chrysosporium*), a part of the N-terminus is missing. No alternative model was available. Irrespective of model #39999 problems, the protein still has the hydrophobin signature with the 8 cysteine residues. This may be a pseudogene. |
| fgenesh1_kg.13_#_24_#_Locus860v1rpkm170.19 | 18178 | [scaffold_13:193774-194615](http://genome.jgi.doe.gov/cgi-bin/browserLoad?db=Phlgi1&position=scaffold_13:193774-194615) | - | - | - | The closest homologue found was collagen type I alpha 2 from *Homo sapien* ([hsa:402382 LOC402382](http://www.genome.jp/dbget-bin/www_bget?hsa:402382)). But a small portion (18%) of the coding sequence of this protein showed homology with hydrophobins from *Coprinopsis cinerea*. The coding sequence of the gene has both start and stop codons with 4 exons and 3 introns. However the C-terminal part of the protein was exceptional longer than the normal hydrophobins identified. This was because the C-terminal part fused with another protein sequence. A thorough check on the 3-frame translation showed that a part of the ORF was removed as intron. By replacing this part and truncating the additional sequence fused to the C-terminal part, a truncated sequence that has a full hydrophobin signature could be obtained. |
| fgenesh1_kg.13_#_23_#_Locus1428v1rpkm104.99 | 18177 | [scaffold_13:189837-190981](http://genome.jgi.doe.gov/cgi-bin/browserLoad?db=Phlgi1&position=scaffold_13:189837-190981) | - | - | - | The coding sequence of the protein has unusually 7 short exons of variable sizes and 6 introns with the start and stop codons fully represented. This is quite unusual for fungal hydrophobins but the sequence has hydrophobin signature. However, in the closest ortholog, *P.chrysosporium*, the coding sequence of the protein has 4 exons of variable sizes with the 4^th^ exon being very large in size. Further examination of the protein sequence showed that the C-terminal part fused to another protein  thereby making the protein unsually longer than other known hydrophobins. |
| estExt_fgenesh1_pm.C_80009 | 27800 | [scaffold_8:34012-34783](http://genome.jgi.doe.gov/cgi-bin/browserLoad?db=Phlgi1&position=scaffold_8:34012-34783) | - | - | - | The existing protein model is fine but has some aa sequences that are obviously lacking or probably degraded in other hydrophobins analysed. This sequence looks like unspliced intron but lacks the exon –intron boundary. |
| gm1.1144_g | 114388 | scaffold_8:39934-40416 |  |  |  | The existing model appears to be fine but the protein was unusually longer than other identified hydrophobins in *P. gigantea*. The sequence also presented some regions that could not be found in other hydrophobins from *P. gigantea*, these abnormal sequences could be due to mutation or other evolutionary forces. Closer examination of the protein sequence showed the cysteine residues could not align properly with other hydrophobins analysed. |
| CE323408_258 | 454788 | [scaffold_8:36686-37411](http://genome.jgi.doe.gov/cgi-bin/browserLoad?db=Phlgi1&position=scaffold_8:36686-37411) | - | - | - |  |
| CE323442_444 | 454822 | scaffold_8:37817-38664 |  |  |  |  |
