# Supplementary material for: Analysis of the Phlebiopsis gigantea Genome, Transcriptome and Secretome Provides Insight into Its Pioneer Colonization Strategies of Wood
Source: PLoS Genet. 2014 Dec 4;10(12):e1004759. doi: 10.1371/journal.pgen.1004759 (PMC4256170; doi:10.1371/journal.pgen.1004759)
Supplement: Table S18 — Fungal species and hydrophobin gene number used for phylogenomics analysis. (DOCX) [file pgen.1004759.s053.docx]

| **Table S18**: Fungal species used for phylogenomics analysis and the number of hydrophobins in each | | | | | |
| --- | --- | --- | --- | --- | --- |
| Fungal phyllum | Fungal species | Predicted No. of hydrophobins in the genome | | | No. of hydrophobins used  in the phylogenetic analysis |
| Basidiomycota | *Ceriporiopsis subvermispora* | | 24 | 21 | |
| Basidiomycota | *Phlebiopsis gigantea* | | 20 | 14 | |
| Basidiomycota | *Phanerochaete* *chrysosporium* | | 17 | 15 | |
| Basidiomycota | *Ganoderma sp* | | 17 | 17 | |
| Basidiomycota | *Phlebia brevispora* | | 22 | 22 | |
| Basidiomycota | *Serpula lacrimans* | | 19 | 18 | |
| Basidiomycota | *Wolfiporia cocos* | | 3 | 2 | |
| Basidiomycota | *Heterobasidion annosum* | | 13 | 13 | |
| Basidiomycota | *Schizophyllum commune* | | 14 | 13 | |
| Basidiomycota | *Coprinopsis cinerea* | | 33 | 22 | |
| Basidiomycota | *Laccaria bicolor* | | 13 | 12 | |
| Basidiomycota | *Ustilago maydis* | | 2 | 1 | |
| Ascomycota | *Acremonium alcalophilum* | | 1 | 1 | |
