# Supplementary material for: Analysis of the Phlebiopsis gigantea Genome, Transcriptome and Secretome Provides Insight into Its Pioneer Colonization Strategies of Wood
Source: PLoS Genet. 2014 Dec 4;10(12):e1004759. doi: 10.1371/journal.pgen.1004759 (PMC4256170; doi:10.1371/journal.pgen.1004759)
Supplement: Table S19 — RNAseq analysis of P. gigantea hydrophobins. (DOCX) [file pgen.1004759.s054.docx]

| **Table S19**. RNAseq analysis of *P. gigantea* hydrophobins | | | | | | | | | |
| --- | --- | --- | --- | --- | --- | --- | --- | --- | --- |
|  | RPKM values | | | NELP/Glu | | ELP/Glu | | NELP/ELP | |
| Protein Ids | NELP | ELP | Glu | Ratio | Prob | Ratio | Prob | Ratio | Prob |
| 124694 | 12.92 | 5.90 | 1.92 | 6.73 | 0.02 | 3.07 | 0.05 | 2.19 | 0.005 |
| 17842 | 5.31 | 1.88 | 2.24 | 2.39 | 0.17 | 0.84 | 0.81 | 2.82 | 0.070 |
| 20660 | 14.43 | 7.04 | 49.56 | 0.29 | 0.05 | 0.14 | 0.02 | 2.05 | 0.17 |
| 127149 | 10.50 | 9.82 | 10.77 | 0.98 | 0.94 | 0.91 | 0.79 | 1.07 | 0.71 |
| 509314 | 22.41 | 21.83 | 4.52 | 4.95 | 0.09 | 4.83 | 0.07 | 1.03 | 0.94 |
| 104620 | 2.17 | 2.43 | 9.91 | 0.22 | 0.14 | 0.25 | 0.15 | 0.89 | 0.80 |
| 69703 | 2.61 | 3.05 | 0.65 | 3.00 | 0.06 | 4.68 | 0.06 | 0.85 | 0.60 |
| 39115 | 1.14 | 1.55 | 0.84 | 1.37 | 0.63 | 1.85 | 0.33 | 0.74 | 0.49 |
| 27800 | 8.82 | 10.22 | 30.42 | 0.29 | 0.17 | 0.34 | 0.19 | 0.86 | 0.36 |
| 114388 | 0.53 | 0.51 | 1.93 | 0.28 | NaN | 0.27 | NaN | 1.04 | 0.97 |
| 454788 | 4.49 | 7.02 | 4.85 | 0.93 | 0.89 | 1.45 | 0.42 | 0.64 | 0.37 |

NaN, RPKM values <1.0
