# Supplementary material for: Analysis of the Phlebiopsis gigantea Genome, Transcriptome and Secretome Provides Insight into Its Pioneer Colonization Strategies of Wood
Source: PLoS Genet. 2014 Dec 4;10(12):e1004759. doi: 10.1371/journal.pgen.1004759 (PMC4256170; doi:10.1371/journal.pgen.1004759)
Supplement: Table S20 — P. gigantea ABC models. (DOCX) [file pgen.1004759.s055.docx]

| **Table S20.** *P. gigantea* ABC models | | | | | |
| --- | --- | --- | --- | --- | --- |
| **Existing model** | | | **Suggested model / changes** | | |
| Model name | Protein ID | Location | Model name | Protein ID | Location |
| estExt_Genewise1.C_390060 | 87882 | scaffold_39:94658-97723 (-) | estExt_Genewise1Plus.C_390061 | 104024 | scaffold_39:94658-97792 (-) |
| estExt_Genewise1.C_810023 | 90887 | scaffold_81:44869-49148 (-) | estExt_Genemark1.C_810016 | 128458 | scaffold_81:44869-49150 (-) |
| fgenesh1_pm.3_#_77 | 21134 | scaffold_3:295746-296813 (-) | estExt_Genemark1.C_30113 | 124233 | scaffold_3:295746-298376 (-) |
| fgenesh1_pm.137_#_8 | 25601 | scaffold_137:31582-34289 (+) | CE62642_3357 | 194022 | scaffold_137:31433-34608 (+) |
| estExt_fgenesh1_pm.C_10057 | 27132 | scaffold_1:171968-174644 (+) | Sequencing gap (N in the position 1891) of about 30 bp. As a result, I’d suggest that there is no intron (and no frame-shift) between exons 6 and 7. |  |  |
| estExt_fgenesh1_pm.C_3030004 and CE204994_3653 | 32454 and 336374 | scaffold_303:11013-12222 (-) and scaffold_303:12521-13899 (-) | estExt_Genewise1.C_3030007 | 96729 | scaffold_303:11013-13936 (-) |
| estExt_Genewise1Plus.C_30270 | 98346 | scaffold_3:356376-357602 (-) | N-terminal part of the gene is missing from the database; the model can be extended upstream based on homology with the related *P. gigantea* proteins (proteins ID 124233 and 96729). | 534834 | scaffold_3:356376-358055 |
| estExt_Genewise1Plus.C_1940030 | 111230 | scaffold_194:39232-40470 (-) | CE123138_2353 | 254518 | scaffold_194:39105-42027 (-) |
| fgenesh1_pg.113_#_1 | 14688 | scaffold_113:148-1448 (-) | Only small fragment of the gene is present and not possible to reconstitute the entire protein sequence |  |  |
| fgenesh1_pg.113_#_2 | 14689 | scaffold_113:2091-7842 (-) | There are two sequence gaps within the gene, so it’s not possible to reconstitute the entire aa sequence. | 534835 | scaffold_113:2091-8447 (-) |
| fgenesh1_pm.41_#_23 | 23474 | scaffold_41:102552-108846 (+) | CE244673_18999 | 376053 | scaffold_41:101782-108976 (+) |
| fgenesh1_pm.30_#_32 | 22983 | scaffold_30:154474-160810 (-) | fre_1_e_gw1.30.123.1 | 534837 | scaffold_30:154474-160810 (-) |
| estExt_fgenesh1_pg.C_1130003 | 36552 | scaffold_113:14632-21227 (-) | There are two gaps within the sequence, thus it is not possible to reconstitute the entire protein sequence. | 534838 | scaffold_113:14632-21227 (-) |
| estExt_fgenesh1_pg.C_2980004 | 38168 | scaffold_298:10908-17662 (+) | fre_1_estExt_Genemark1.C_2980008 | 534839 | scaffold_298:10770-17662 (+) |
| gm1.8277_g | 121521 | scaffold_175:36930-45224 (-) | fre_1_gm1.8277_g | 534840 | scaffold_175:36930-43613 (-) |
| estExt_Genemark1.C_570036 | 127559 | scaffold_57:121648-128305 (+) | CE282348_2765 | 413728 | scaffold_57:121475-128597 (+) |
| CE33064_4524 | 164444 | scaffold_113:38450-44018 (+) | fre_1_estExt_Genemark1.C_1130009 | 534841 | scaffold_113:37502-44008 (+) |
| MIX8954_2964_12 | 497319 | scaffold_181:40693-47165 (+) | CE111560_17494 | 242940 | scaffold_181:39964-46863 (+) |
| fgenesh1_pg.118_#_1 | 14797 | scaffold_118:58-2352 (+) | More than half of the gene is missing, so it’s not possible to reconstitute the entire sequence of the deduced protein |  |  |
| fgenesh1_pm.70_#_13 | 24318 | scaffold_70:38980-41804 (+) | estExt_Genemark1.C_700014 | 127965 | scaffold_70:38894-42042 (+) |
| estExt_fgenesh1_pm.C_230030 | 28761 | scaffold_23:117477-120357 (+) | fre_1_fgeneshTR_pm.23___29 | 534842 | scaffold_23:117477-120256 (+) |
| CE60640_8249 | 192020 | scaffold_135:12700-14524 (+) | estExt_Genemark1.C_1350008 | 129467 | scaffold_135:12058-14576 (+) |
| fgenesh1_kg.274_#_2_#_Locus4143v1rpkm41.13 | 20542 | scaffold_274:5209-7367 (+) | CE182818_10251 | 314198 | scaffold_274:5006-7492 (+) |
| estExt_fgenesh1_pg.C_400020 | 34896 | scaffold_40:97140-100665 (-) | There is a sequence gap of about 270 bp within the gene, thus it’s not possible to reconstitute the entire protein sequence |  |  |
| estExt_Genewise1Plus.C_340085 | 103495 | scaffold_34:113777-115000 (-) | CE219763_4564 | 351143 | scaffold_34:113432-115436 (-) |

*-amino acids VNIQAS (positions 100 – 105) should be replaced by residues KGQLLEI as in the protein model 25601
