# Supplementary material for: Analysis of the Phlebiopsis gigantea Genome, Transcriptome and Secretome Provides Insight into Its Pioneer Colonization Strategies of Wood
Source: PLoS Genet. 2014 Dec 4;10(12):e1004759. doi: 10.1371/journal.pgen.1004759 (PMC4256170; doi:10.1371/journal.pgen.1004759)
Supplement: Table S21 — Chemical composition of lipids from Loblolly pine wood (Pinus taeda). (DOCX) [file pgen.1004759.s056.docx]

**Table S21.** Chemical composition of lipids from Loblolly pine (*Pinus taeda*), expressed as milligrams per grams of oven dry wood

| **Compounds** | **Content (mg g^-1^)** |
| --- | --- |
| **Fatty acids** | **6.34** |
| Palmitic acid (C16:0) | 0.69 |
| Margaric acid (C17:0) | 0.15 |
| Linolenic acid (C18:3) | 0.41 |
| Linoleic acid (C18:2) | 2.28 |
| Oleic acid (C18:1) | 2.58 |
| Stearic acid (C18:0) | 0.22 |
| **Resin acids** | **25.92** |
| Pimaric acid | 2.89 |
| Sandaracopimaric acid | 0.59 |
| Isopimaric acid | 0.64 |
| Palustric acid | 4.63 |
| Levopimaric acid | 3.93 |
| Dehydroabietic acid | 5.22 |
| Abietic acid | 5.06 |
| Neoabietic acid | 2.34 |
| 7-Oxo-dehydroabietic acid | 0.18 |
| 15-Hydroxydehydroabietic acid | 0.46 |
| **Monoglycerides** | **0.40** |
| **Sitosterol** | **0.15** |
| **Diglycerides** | **0.66** |
| **Sitosterol esters** | **0.44** |
| **Triglycerides** | **7.20** |
| **Total acetone extract** | **56.42** |
