# Supplementary material for: Analysis of the Phlebiopsis gigantea Genome, Transcriptome and Secretome Provides Insight into Its Pioneer Colonization Strategies of Wood
Source: PLoS Genet. 2014 Dec 4;10(12):e1004759. doi: 10.1371/journal.pgen.1004759 (PMC4256170; doi:10.1371/journal.pgen.1004759)
Supplement: Text S1 — Detailed description of methods and annotated gene families. (DOCX) [file pgen.1004759.s057.docx]

**Supporting Information**

**Appendix**

**1. Supplemental Information Text**

1.1 Strain selection, assembly and annotation, principal component analyses

1.2 Environmental signaling

1.3 Genes involved in ligninolysis

1.3.1 Heme peroxidases

1.3.2 Copper radical oxidases

1.3.3 GMC oxidoreductases

1.4 Iron homeostasis and MCOs

1.5 Monosaccharide catabolism

1.6 Carbohydrate active enzymes (CAZys)

1.7 Cytochrome P450s and secondary metabolism

1.8 Hydrophobins and ABC proteins

1.9 RNAseq and LC/MS methods

1.10 Analysis of loblolly pine extract

**2. References**

**S.1. *Phlebiopsis gigantea* strain selection, genome assembly and annotation**

Aerial spore capture was used to collect *P. gigantea* isolate 11061-1 in Macon County, Alabama. It was confirmed as a *P. gigantea* isolate by sequencing its ribosomal RNA internal transcribed spacer region. Isolate 11061-1 was cultured on 2% Bacto malt extract agar, wrapped in parafilm, and incubated upside down at room temperature (23ºC) until basidiospores collected on the lid of the petri dish (after 3-4 weeks). Single basidiospore isolates 5-1 to 5-10 were isolated from progeny of 11061-1.

Aspen (*Populus tremuloides*), pine (*Pinus taeda*) and spruce (*Picea glauca*) wood block decay studies were conducted as indicated in the methods section of the paper. Based on these experiments (Figure 1), single spore isolate 5-6 was selected for genome sequencing.

**Genome assembly and annotation**

The genome of *Phlebiopsis gigantea* 5-6 was sequenced using Illumina. A standard paired-end library with 200bp insert size was sequenced in 2x76 bp read format. General aspects of library construction and sequencing can be found at the JGI website <http://www.jgi.doe.gov/>.  No contaminants were detected. Following steps were then performed for assembly: (1) Illumina reads were assembled using Velvet (velvet 1.1.01) (2) Velvet contigs and 454 reads were assembled using Newbler (version 2.5-internal-10Apr08-1) (3) 3 kb simulated paired end reads were built from Newbler contigs, and (4) Illumina reads were assembled with simulated read pairs using Allpaths–LG (version R36892). This resulted in a 30.1Mbp genome assembly with 573 scaffolds (N50=120Kbp), 1245 contigs (N50=57.0Kb) and 144x read depth coverage. 94% RNA-Seq reads were aligned to the genome assembly. Unaligned reads may reflect missing regions in the assembly or contaminant RNA. Mito was assembled separately with AllPathsLG to produce one 86.6Kbp scaffold.

Total RNA from two *Phlebiopsis* samples (avicel- and loblolly pine-containing cultures) were used to generate two stranded RNASeq libraries. mRNA was purified from total RNA using the Absolutely mRNA™ purification kit (Stratagene). The isolation procedure was performed twice to ensure the sample is free of rRNA. Subsequently, the mRNA samples were chemically fragmented to the size range 200-250 bp using 1x fragmentation solution for 5 minutes at 70 C (RNA Fragmentation Reagents, AM8740 – Zn, Ambion). First strand cDNA was synthesized using Superscript II Reverse Transcriptase (Invitrogen) and random hexamers. cDNA was purified with Ampure SPRI beads. Then the second strand was synthesized using a dNTP mix (with dTTP replaced with dUTP) *E.coli* RnaseH, DNA Ligase, and DNA polymerase I for nick translation. The dscDNA were purified and selected for fragments in the range 200-300bp using a double Ampure SPRI bead selection. The dscDNA fragments were then blunt-ended, poly A tailed, and ligated with Truseq adaptors using Illumina DNA Sample Prep Kit (Illumina). Adaptor-ligated DNA was purified using Ampure SPRI beads. Then the second strand was removed by AmpErase UNG (Applied Biosystems) similar to the method described by Parkhomchuk et al. [1]. Digested cDNA was again cleaned up with Ampure SPRI beads. Paired end 76 bp reads were generated by sequencing using the Illumina HiSeq instrument.

*De novo* transcript contig assembly was done using Rnnotator [2]. The Rnnotator assembly pipeline consists of three major components: preprocessing of reads, assembly, and postprocessing of contigs. 325 million of paired-end 100 bp Illumina reads of stranded RNA-seq data were used as input. Preprocessing consisted of removal of low-quality reads, low-complexity reads, adapter-containing and duplicate reads. Assembly was completed with Velvet (eight runs of velvet were performed with hash lengths of 53, 59, 65, 71, 77, 89, 83, 95). Minimum contig length was set at 100. Duplicate contigs were removed using Vmatch (v. 2.1.4). Contig postprocessing included merging, splitting misassembled contigs, contig extension and polishing using the strand information of the reads. Post-processed contigs were clustered into loci and putative transcript precursors were identified.

Assembly and annotation statistics are listed in Tables S1 and S2, respectively. Proteins were assigned to 6412, 5615, 6932 and 2253 KOG categories, GO terms, pfam domains, and EC numbers, respectively. In addition to these assignments, detailed functional annotation includes Signal P, TMHMM, BLASTp against NCBI NR and manual curation. All are available via the JGI Genome portal.

We used the MCL analysis tool implemented in the *P. gigantea* website (http://genome.jgi.doe.gov/Phlgi1/Phlgi1.home.html) to search for PFAM clusters in Agaricomycotina. The most abundant clusters in *P. gigantea* included a domain for which no PFAM domain had yet been defined. Also prevalent were WD-40, NmrA and amidohydrolase_1 domains. (The NmrA domain includes short chain dehydrogenases/reductases [3]). Several PFAM families involved in oxidative reactions, e.g. cytochrome P450; N-terminal alcohol dehydrogenases, and MFS/sugar permeases were widespread. The most abundant transcriptional regulator family comprises proteins with BTB/POZ domains, which typically mediate transcriptional repression and interact with components of histone deacetylase co-repressor complexes. No PFAM cluster exceeded those found in other basidiomycetes.

Individual proteins from these clusters exhibited highest similarity to *Phanerochaete carnosa* or *P. chrysosporium*. This similarity was reflected in a high level of synteny between *Phlebiopsis* and *Phanerochaete* (Figure S1). Focusing on genes likely involved in lignocellulose degradation (73 CAZy families and 12 AAs; Dataset S1), principal component analysis (PCA) firmly grouped *P. gigantea* among white rot fungi (Figures 2, S2, S3).

**S.1.2. Environmental signalling**

**Heterotrimeric G-protein signaling**

Heterotrimeric G-protein signaling represents one of the most important pathways for reception and transmission of environmental signals. *P. gigantea* has three G-protein alpha subunits related to *N. crassa* GNA1, GNA2 and GNA3 (Phlgi1_107343, Phlgi1_29521 and Phlgi_78174). Additionally, as also observed for *Postia placenta* [4] and *Ceriporiopsis subvermispora* [5], *P. gigantea* has several homologues of *U. maydis* GBA4 (Phlgi1_16052, Phlgi1_28734, Phlgi1_100177, 106742, Phlgi1_109311, Phlgi1_110379, Phlgi1_111453, Phlgi1_124535, Phlgi1_125945 and Phlgi1_128468). As in *P. placenta* and *C. subvermispora,* a putative G-protein beta subunit (Phlgi_17891) and two G-protein gamma subunits (Phlgi1_23297 and Phlgi1_68598) were detected.

***G-protein coupled receptors***

*P. gigantea* has 3 GprC type GPCRs, 3 mPR type GPCRs and 2 Stm1 type GPCRS. Also the group of STE3-type G-protein coupled pheromone receptors is enlarged with 7 genes. However, in contrast to *P. placenta*, where a comparable number of genes for STE3-type GPCRs was detected, these genes are not clustered in one locus in *P. gigantea*. Only two pairs of STE3-type pheromone GPCR encoding genes were found in close vicinity to each other in the genome (Phlgi1_97054 and Phlgi1_60412 on the end of scaffold 377; Phlgi1_106595 and Phlgi1_128080 on scaffold 74). In tetrapolar species, STE3-type GPCRs are encoded in the *B* mating type loci and are shown to interact with *B* mating-type-specific pheromones [6,7]. Between the expressed genes Phlgi1_97054 and Phlgi1_60412 on scaffold 377 there is an also expressed gene for a pheromone precursor (Phlgi1_534820) as would be indicative for *B* mating type loci in tetrapolar species. Other putative pheromone precursors are encoded by four genes located on scaffold 66 (Phlgi1_534792, Phlgi1_534790, Phlgi1_534826, Phlgi1_534997). As in *P. placenta* [4], *P. gigantea* is heterothallic bipolar [8,9] with a divergently transcribed *HD1* and *HD2* gene pair for homeodomain transcription factors (Phlgi1_534802 and Phlgi1_434801). In tetrapolar species, the *HD* genes are found in the *A* mating type locus [6,7]. In all other bipolar cases analyzed so far, the *HD* locus has been shown to confer mating type activity and the pheromone receptor genes and pheromone precursor genes are without mating type activity. Data suggest that loss of *B* mating type control by the pheromone-pheromone receptor system is the derived situation [7,10,11].

Strikingly, *P. gigantea* encodes 10 predicted microbial opsins (Figure S4). While some homologues to *C. subvermispora* were found as well, the number of opsin genes in *P. gigantea* still exceeds that of *C. subvermispora*. Besides opsins related to those detected in ascomycetes such as *N. crassa* or *A. nidulans*, opsin related genes of *P. gigantea* and *C. subvermispora* form an individual clade also comprising the single opsin related gene of *P. placenta*.

The physiological function of opsins in fungi has not yet been described in detail. They are assumed to serve as sensory receptors for light dependent events [12]. *N. crassa* NOP1 was shown to form a green light absorbing pigment with retinal and to undergo a photocycle [13]). Along with the well-equipped toolbox of *P. gigantea* for light signal perception as discussed below, these findings indicate an intriguing relevance of light for the physiology of this fungus.

**Light response**

The genes of three putative photoreceptors related to *N. crassa* White collar-1 (Phlgi1_72862 and Phlgi1_92699) and White collar-2 (Phlgi1_62123) were detected in the genome of *P. gigantea*. While RNAseq analysis clearly showed transcription of these genes, no significant regulation was observed. Multiple photoreceptors related to WC1 and WC-2 were also detected in *Phycomyces blakesleeanus* [14]. In order to evaluate whether the two WC-1 related genes might be related to these genes, we did a phylogenetic analysis (Figure S5). For *P. blakesleeanus* only an involvement of MADA and MADB, which are related to WC-1 and WC-2, respectively, in phototropism was shown. The remaining five homologues to these photoreceptors are assumed to have additional sensing functions for light dependent events, which are not studied in detail so far.

While photoreceptors related to *N. crassa* WC-1 or WC-2 could clearly be distinguished, a specific relationship to one of the *P. blakesleeanus* proteins was not observed. Due to its close relationship with *N. crassa* WC-1, Phlgi1_72862 appears to be the major photoreceptor of *P. gigantea*. Albeit more distantly related and lacking the characteristic LOV domain (Figures S5 and S6), a function in light signal reception is also likely for Phlgi1_92699, which however might also have assumed novel functions. Orthologues of *N. crassa* VVD or *Trichoderma reesei* ENV1 could not be identified. Phlgi1_62123 represents the putative WC-2 related photoreceptor of *P. gigantea* and appears to be conserved in several basidiomycetes.

The *P. gigantea* genome also contained one class I photolyase (Phlgi1_32310), a protein related to fungal VELVET proteins (Phlgi1_35880), one related to the blue light inducible protein BLI-3 (Phlgi1_124744) and two proteins related to an RNA helicase involved in regulation of the circadian clock in *N.* *crassa* (Phlgi1_99229 and Phlgi1_489254). However, no homologue encoding the major regulator of circadian rhythmicity in *N. crassa*, FRQ could be detected.

In summary, the light signaling machinery in *P. gigantea* comprises a striking enrichment in opsins, suggesting an increased relevance of green-light related phenomena. Nevertheless, the presence of two WC-1 related blue light photoreceptors as well as at least one putative phytochrome (Phlgi1_507654) indicate sensitivity to blue and red light as well. With respect to circadian rhythmicity, the lack of a FRQ homologue is consistent to FRQ being absent in basidiomycetes. Nevertheless, the extensive toolset for light perception it is unlikely that circadian rhythms are not present in *P. gigantea* or in other basidiomycetes.

**Calcium signalling**

Although most signal transduction components are considered intracellular proteins, several of these proteins were found in the secretome of *Phlebiopsis gigantea*. Interestingly, two candidate calmodulin proteins as well as a putative calnexin (Phlgi1_123186, Phlgi1_20072 and Phlgi1_17911), which are acting in the calcium signaling pathway were among the proteins found to be secreted. Since detection of the peptides shows significant values also at early timepoints, it is unlikely that autolysis has caused these proteins to be released into the culture filtrate.

While secretion of calmodulin into the culture filtrate has not been reported so far, translocation of small secretory proteins by the aid of calmodulin, which binds to their signal peptides, was reported. Thereby, calmodulin prevented degradation and aggregation of these proteins and minimized their interactions with other polypeptide-binding proteins [15]. However, this function was shown for chaperoning precursors through the cytosol in mammalians. Search of the Database of Interacting Proteins (DIP) (<http://dip.doe-mbi.ucla.edu/dip/>) in yeast for interactors of these proteins did not hint at a chaperoning function for extracellular proteins. Whether a comparable role for calmodulin in protection of secreted proteins outside the fungal cell is possible, remains to be confirmed.

**Two component phosphorelay systems**

Two component phosphorelay systems represent important signal transmission tools. Upon reception of the signal, a phosphorylation cascade is initiated that involves a histidine kinase, a histidine phosphotransferase and a response regulator receiver. Interestingly, in *P. gigantea* there is a striking cluster of response regulator receivers on scaffold 107 (Figure S7). Screening of the genes surrounding this cluster revealed a remarkable enrichment of transcription factor genes. In *P. chrysosporium*, but not *C. subvermispora* or *P. placenta,* a comparable cluster appears to be present. Transcript levels of several transcription factors are significantly (P<0.01) regulated >2-fold with RPKM values exceeding 10. Significant regulation of a putative aldose epimerase or mutarotase (Phlgi1_128902) as well as a transcriptional regulator (Phlgi1_36458) (Table S3) strongly suggests a physiological relevance of this locus in response to extractives. Since this transcriptional regulator is assumed to be involved in chromatin rearrangement, the whole cluster might be subject to epigenetic regulation.

**S1.3 *Phlebiopsis gigantea* genes involved in ligninolysis**

**S.1.3.1 Heme peroxidase genes in the *Phlebiopsis gigantea* genome**

A preliminary screening of the automatically-annotated genome of *P. gigantea* was performed using “peroxidase” as the search term (http://genome.jgi.doe.gov/Phlgi1/Phlgi1.home.html), and twenty gene models were initially identified. A sequence-by-sequence exhaustive analysis revealed that only 18 of the above gene models encode heme peroxidases. Manual annotation of these gene models was based on multiple alignments with 460 basidiomycete heme peroxidase protein sequences, and examination of theoretical molecular structures obtained by homology modeling using crystal structures of related peroxidases as templates and programs implemented by the automated protein homology modeling server “SWISS-MODEL” (1) (see Dataset S2 for detailed information about the 18 heme peroxidases identified in *P. gigantea*).

The heme peroxidases finally identified could be classified into five different groups as follows: i) Cytochrome *c* peroxidase (CCP) (1 model, Phlgi1_127931); ii) Ligninolytic peroxidases (8 models), including five typical “long” manganese peroxidases (MnP) (Phlgi1_117668, Phlgi1_115592, Phlgi1_115591, Phlgi1_75566, Phlgi1_75572) specific for Mn^2+^ and three lignin peroxidases (LiP) (Phlgi1_30372, Phlgi1_121662, Phlgi1_150531); iii) Low redox-potential generic peroxidases (GP) (1 model, Phlgi1_32509); iv) Heme-thiolate peroxidases (HTP) including chloroperoxidases (CPO) and unspecific peroxygenases (UPO) with a proximal cysteine acting as the fifth heme iron ligand (4 models, Phlgi1_18201, Phlgi1_19534, Phlgi1_104428, Phlgi1_131735); and v) Dye decolorizing peroxidases (DyP) (4 models, Phlgi1_71660, Phlgi1_122124, Phlgi1_85295, Phlgi1_125681) (note that protein model Phlgi1_122124 is the curated version of model Phlgi1_78526 as described below).

Ligninolytic peroxidases could be annotated as LiP and MnP on the basis of the presence or absence of only a few amino acid residues at the active center and substrate oxidation sites [16] after homology modeling. A representation of the homology models obtained for these enzymes, including key amino acid residues of the heme environments, is presented in Figure S8. LiP and MnP models were obtained using the *P. chrysosporium* LiP (PDB entry 1LLP) and MnP (PDB entry 3M5Q) crystal structures as templates. By contrast, the *Trametes cervina* LiP and not the *Coprinopsis cinera* GP (CiP) crystal structure was automatically selected by the SWISS MODEL server as the most appropriate template for modeling the putative GP (protein model Phlgi1_32509). This protein seems to be structurally more closely related to the *T. cervina* LiP (the only LiP described to date containing a catalytic tyrosine [17]) than to CiP. However, the absence of a putative catalytic Tyr exposed to the solvent in the homology model and its position in the dendrogram of Figure S8I (it is grouped together with ten GPs from seven different basidiomycete species) strongly support that protein Phlgi1_32509 is a GP. Analysis of this GP model also revealed the presence of two β-strands not observed in other GPs. This structural feature was finally removed (Figure S6.I) by manual curation of its coding DNA sequence after identification and removal of an extra intron previously not identified by the gene structure prediction.

Regarding the putative HTPs, the four members of this new superfamily identified in the *P. gigantea* genome sequence were modeled with the crystal structure of CPO (PDB entry 2CIW) from the ascomycete *Leptoxyphium fumago*, this being the only fungal HTP crystallized to date. The *Agrocybe aegerita* UPO has been recently crystallized [18] but its molecular structure is not yet available in PDB. Differences in the amino acid residues of the heme environment were observed among the four HTP models (Figure S10), suggesting putative differences in their catalytic properties.

Finally, DyP models were obtained using the *B. adusta* DyP crystal structure (PDB entry 3MM3) as template. Although one of the four DyPs identified in *P. gigantea* (protein model Phlgi1_78526) had to be curated, the curation process only affected the signal peptide and a few amino acids of the N-terminal sequence of the mature protein (yielding the curated model Phlgi1_122124). In consequence, the homology model for this protein was the same before and after curation. Unlike HTP, the four DyP models present the same residues at the proximal and distal sides of the heme active site (Figure S11). Putative variations in biochemical properties among these proteins should be sought in other protein regions, as observed in the case of the ligninolytic peroxidases.

An additional MnP-like protein (Phlgi1_65973) encoded by *P. gigantea* was not considered further as the predicted 157-amino acid polypeptide sequence lacks a C-terminal (around 200 amino acids). This region typically contains the histidine coordinating the heme-iron and one of the three acidic residues forming the Mn-binding site. The gene encoding this protein appears truncated by a gap at the end of contig 3 on scaffold 19. On the other hand, model Phlgi1_65951 was annotated as a putative non-functional MnP. Its amino acid sequence converted into a structural homology model presents the general folding of a typical manganese peroxidase (MnP). However it lacks key residues involved in the heme group coordination and contains only two of the three acidic residues forming the Mn-binding site. These two models could be natural pseudogenes or the result of an error in the sequencing/ assembling processes.

**Comparison with other basidiomycete peroxidases**

Manual annotation of the heme peroxidase gene models was followed by an analysis of the evolutionary relationships among all basidiomycete heme peroxidases in addition to the 18 gene models from the *P. gigantea* genome. The deduced amino-acid sequences of a total of 478 basidiomycete peroxidases were included in this comparison and a dendrogram showing their evolutionary relationships was obtained (Figure S9) (compressed sub-trees are shown to facilitate the *P. gigantea* peroxidases analysis). Sequence comparison was computed as Poisson distances, and clustering was based on the UPGMA method using the "pairwise deletion option" of the MEGA5 program [19]. The heme peroxidase dendrogram of Figure S9 shows clearly differentiated groups corresponding to the superfamily of plant-fungal-bacterial peroxidases, dye-decolorizing peroxidases (DyPs) and HTPs.

The group of the superfamily of plant-fungal-bacterial peroxidases contains: i) Two small clusters accommodating Class I peroxidases (a total of 48 sequences including CCPs from 30 different fungal species, with 1-2 members per genome, that of *P. gigantea* containing only one; and hybrid ascorbate-cytochrome *c* peroxidases (APX-CCP)); and ii) a large cluster comprising class II peroxidases (221 sequences among which there are 30 low redox-potential GPs, one of them from *P. gigantea*, and 191 members of the different families and subfamilies of high redox potential ligninolytic peroxidases described to date. The latter includes short, long, and extralong MnPs, LiPs, VPs, VP-LiP intermediate states, and atypical MnPs and VPs). GenBank accession numbers for sequences from fungal genomes sequenced at JGI are included in the expanded dendrogram of Figure S12, which provides a detailed analysis of the class II peroxidases identified in *P. gigantea*.

With only 64 members, the DyPs are distributed in two clusters (H and I)(Figure S9). The *P. gigantea* genome has four representatives: i) Three of them (protein models Phlgi1_85295, Phlgi1_125681 and Phlgi1_71660) are found in cluster H (formed by only seven sequences) grouped together with DyPs identified in the non-ligninolytic basidiomycetes *P. placenta*, *C. cinerea* and *Laccaria bicolor*; and ii) the fourth one (protein model Phlgi1_122124) is located in cluster I (expanded Figure S13) closely related to DyP sequences from *B. adusta* and *P. brevispora*.

With few exceptions, HTPs have not been biochemically characterized. Distinct from members of the superfamily of plant-fungal-bacterial peroxidases and DyPs (Figure S9), the 141 sequences are organized in four well defined clusters (J-M in Figure S9) with the four *P. gigantea* members located in clusters J, L and M. Cluster K contains the *A. aegerita* ‘unspecific peroxygenase’ (UPO), the most thoroughly studied representative enzyme [20-24].

The class II peroxidase cluster, containing low redox-potential GPs and high redox-potential ligninolytic peroxidases, is shown expanded in Figure S12. As observed, the three LiP gene models identified in *P. gigantea* are located in cluster B together with all LiP sequences identified to date, with the only exception being the *T. cervina* LiP that, as previously indicated, exhibits a catalytic tyrosine [17] instead of the typical catalytic tryptophan [25]. These three LiPs are closely related to both the *P. chrysosporium* and *T. versicolor* LiPs and the three VPs identified in the *T. versicolor* genome sequence. Cluster B is embedded in cluster A, where short MnPs and VPs are grouped with different atypical MnP and VP forms lacking one of the three acidic residues forming the typical Mn-binding site. The five *P. gigantea* MnP gene models (corresponding to long MnPs) appear in the same sub-cluster of cluster C where all the long and extralong MnPs are grouped separately from cluster A. Finally, the only GP model identified in *P. gigantea* (Phlgi1_32509) is clustered together with other GPs from 8 different fungal species in cluster D. These are more closely related with the ligninolytic peroxidases than with GPs and atypical MnPs from *A. delicata* appearing in the more distant group E.

**Peroxidase gene expression analysis**

Analysis of peroxidase gene expression levels (Table 1; Dataset S2) revealed significant transcript accumulation (12.8-fold up-regulation) of DyP 85295 in acetone extracted loblolly pine (ELP) relative to media containing glucose (Glu) as sole carbon source. However, peptides corresponding to DyP Phlgi1_85295 were not detected in any media filtrates. No other peroxidase exhibited significant (P<0.01) transcript accumulation under the conditions examined, but a second DyP (Phlgi1_122124) showed a modest increase (>5-fold; P=0.04)) in loblolly pine (LP) relative to Glu medium (Dataset S2). Further, emPAI values for this DyP were extremely high, ranking fifth among all the proteins identified in the secretome analysis of LP medium. Also, the Phlgi1_122124 protein model is the only peroxidase of this super-family with a signal peptide predicted by SignalP.

A recent study of 31 fungal genomes has revealed that DyP-encoding genes are widespread among the white rot fungal genomes [26], ranging from only one gene identified in the genome sequences of *H. annosum* and *D. squalens* to the eleven genes from *A. delicata*. *P. gigantea* presents an intermediate number (four genes) like *Fomitiporia mediterranea* (three genes) and *Punctularia strigosozonata* (five genes). However, this type of peroxidase is not unique to white-rot fungi. The brown-rot model fungus *P. placenta* contains two DyP genes in its genome sequence, and genes for these peroxidases have been also identified in other fungi, including the ectomycorrizhal *L. bicolor* (two genes) and the coprophilic *C. cinerea* (four genes), among others. The presence of DyP-coding genes in these fungal strains, as well as their absence in the white-rot model fungus *P. chrysosporium*, suggest that DyPs are not ligninolytic enzymes. However, Liers *et al.* [27] have demonstrated that a DyP-type peroxidase isolated from cultures of the white-rot fungus *A. auricula-judae* growing on beech wood catalyzes the oxidative breakdown of a nonphenolic β-O-4 lignin model dimer in a similar extent to that observed with the ligninolytic VP from *B. adusta*. All the above suggest that fungal DyPs could be involved in different processes including ligninolysis, acting directly on lignin (still to be demonstrated) or on metabolites generated from its depolymerization.

**S1.3.2 Copper radical oxidases (CROs)**

A blastp search of the *P. gigantea* genome with mature *P. chrysosporium* glyoxal oxidases (PcGLX) indicated six CROs in the genome sequence (Table S4). Alignments identified conservation of C70, Y135, YH377-378 and H471 active site amino acids [28]. These putative *P. gigantea* genes correspond to *P. chrysosporium* GLX, CRO1, two CRO2s, CRO5 and CRO6 [29]. The protein ID Phlgi1_327975 (CRO6) has a probable transmembrane region. Peptides for the CRO5 and CRO2-like proteins were detected in filtrates from ELP and NELP cultures. Partial sequence for another CRO3/4/5 was identified on scaffold 65. The N-terminal has been assigned protein ID Phlgi1_118353, the C-terminal has protein ID Phlgi1_71528, and there is a considerable sequence gap between these regions. Alignments did not allow verification of active site due to lack of sequence in those regions.

**S1.3.3** **GMC oxidoreductases and alcohol dehydrogenases**

No pyranose dehydrogenase (PDH) and glucose dehydrogenase (GDH) gene models were localized in the genome of *P. gigantea*. Multiple sequence alignments of localized GMC models from *P. gigantea* with well-known GMC proteins identified conserved regions and catalytic active site residues for the following:

1. Aryl-alcohol oxidase (AAO) models: Phlgi1_121514 and Phlgi1_128071 (Figure S14)
2. Methanol oxidase (MOX) models: Phlgi1_120749, Phlgi1_108516 and Phlgi1_72751 (Figure S15)
3. Cellobiose dehydrogenase (CDH) model: Phlgi1_99876 (Figure S16)
4. Pyranose oxidase (POX) model: Phlgi1_130349 (Figure S17)
5. Glucose oxidase (GOX) model: Phlgi1_128108 and Phlgi_123211 (Figure S18)

Physiologically related to GMCs, but belonging to the zinc-type alcohol dehydrogenase superfamily, are aryl-alcohol dehydrogenases (AADs) with 8 models (Phlgi1_148078, Phlgi1_127405, Phlgi1_89048, Phlgi1_101518, Phlgi1_115124, Phlgi1_406426, Phlgi1_147847 and Phlgi1_105456) in the *P. gigantea* genome (Figure S19).

Protein and transcript expression of these genes are summarized in Table S5. CDH Phlgi1_99876 and MOX Phlgi1_120749 models are highly expressed. In addition to CDH, significant regulation was observed for MOX Phlgi1_108516 and GOX Phlgi1_128108. Transcipt levels corresponding to AAD proteins Phlgi1_89048 and Phlgi1_105456 were high in NELP- and ELP-media. Similar to a more distantly related AAD-like gene (Phlgi1_30343; Table 1; Dataset S2) the Phlgi1_89048 levels were significantly higher in the non-extracted medium (NELP). These results are consistent with a role for these dehydrogenases in the metabolism of host extractives.

**S.1.4 Iron homeostasis and MCOs.**

Due to their close connection to iron metabolism we carefully annotated the multicopper oxidases (MCOs) of *P. gigantea* (Table S6). The *P. chrysosporium* MCO1 and Fet3 protein sequences, as well as the *C. subvermispora* Lcs-1 (laccase) were BLASTed against the *P. gigantea* genome database. In addition, we searched the *P. gigantea* automated annotations with the keywords “laccase” and “multicopper oxidase”. These protein models are summarized in Table S6, while phylogenetic comparisons are shown in Figure S20. Close inspection of the enzyme signature regions suggested variation in enzyme function (Figure S21).

Thus*, P. gigantea* has five different genes for complete MCOs with three typical Cu-oxidase motifs (there is an additional partial gene sequence; Phlgi1_ 38701) but, similar to *P. chrysosporium* and *P. carnosa*, no gene for a *sensu stricto* laccase [30-32] (Figure S20).

One gene in *P. gigantea* encodes a sequence for a typical Fet3-type ferroxidase (Phlgi1_77659), whereas the other four [Phlgi1_534793; Phlgi1_534795; Phlgi1_534796; Phlgi1_534797] group within a cluster of MCOs (Figure S20) that contain enzymes of (dual) ferroxidase and laccase activities [33,34]. SignalP analysis predicts signal peptides for Phlgi1_77659, Phlgi1_534796 and Phlgi1_534797 but not for Phlgi1_534793 and Phlgi1_534795. As typical for ferroxidases, Phlgi1_77659 is longer in sequence than the other MCOs due to the presence of a C-terminal transmembrane (TM) domain distinctive for the plasma-membrane-localized ferroxidases [35,36]. Also typical for *fet3*-type genes [30,37], the gene for the putative ferroxidase Phlgi1_77659 is directly linked in divergently transcribed orientation to a gene *ftr1* for an iron permease FTR1 (Phlgi1_77664) that in analogy to characterized systems will be required for uptake of Fe^3+^ after Fet3-mediated oxidation of Fe^2+^ [38].

Closest relatives of *P. chrysosporium* are Fet3 (to Phlgi1_77659), MCO2 and MCO3 (to Phlgi1_534793) and MCO1 and MCO4 (to Phlgi1_534795; Phlgi1_534796; Phlgi1_534797), respectively. The closest relative of Phlgi1_534797 in GenBank is PfaL (Figure S20), an enzyme of *Phanerochaete flavido-alba* (ABR15762) that has reasonable laccase activity and low ferroxidase activity [34]. The extracellular MCO1 of *P. chrysosporium* in contrast has strong ferroxidase activity and low laccase activities ([33,34]).

In contrast to all enzymes with considerable ferroxidase activity, the *bona fide* laccase PfaL and *P. chrysosporium* MCO4 were seen before to differ from all other enzymes in the ferroxidase/laccase cluster much in length and sequence of two of the four loops expected in ferroxidases to form the substrate pocket for Fe^2+^ binding and oxidation [37]. Phlgi1_534795, Phlgi1_534796, and Phlgi1_534797 resemble in sequence PfaL and *P. chrysosporium* Mco4 whereas Phlgi1_534793 is more similar in sequence to *P. chrysosporium* MCO1 (Figure S19) showing the stronger ferroxidase activity [33]. In the Fet3 protein of the ascomycete *S. cerevisiae,* the corresponding loops contain amino acids critical to contact Fe^2+^ and for the electron transfer pathway [39-42]. All critical amino acids are missing in PfalL, *P. chrysosporium* MCO4, the three related enzymes of *P. gigantea* and also seven putative MCOs of *P. carnosa*. As *P. chrysosporium* MCO1 and *P. gigantea* Phlgi1_534793, two other MCOs of *P. carnosa* resemble in sequence the four loops of canonical Fet3 ferroxidases. In addition, *P. carnosa* has one gene for a Fet3-type ferroxidase.

*P. carnosa* and *P. chrysosporium* are reported to not exhibit laccase activity [32,43], whereas for *P. gigantea* there are contradictory reports and strains seem to widely differ in strength of laccase activities [44,45]. By similarity in their loops for the substrate pockets to *P. carnosa* PfaL, Phlgi1_534795, Phlgi1_534796, and Phlgi1_534797 are prime candidates to look for laccase-like enzymatic activity, but as mentioned, our analysis does not support the presence of *sensu stricto* laccase proteins. Consistent with this possibility, peptides corresponding to Phlgi1_534795 and Phlgi1_534796 were easily detected in LP media. Interestingly, the gene encoding Phlgi1_534795 was the only gene significantly upregulated in ELP relative to NELP media (Dataset S2; Table 3).

**Ferric reductases.**

Using a similar strategy as mentioned above, we also looked for proteins with potential iron reductase activity. Gene models are listed below. No clustering or significant gene regulation was observed.

| ***P. gigantea* ID** | **Position** | **Description, notes** |
| --- | --- | --- |
| 77256 | scaffold_169:55780-57536 | Ferric reductase activity |
| 123760 | scaffold_1:54688-57138 | Ferric reductase activity |
| 125089 | scaffold_11:264401-266693 | Ferric reductase activity |
| 96160 | scaffold_250:15115-17510 | Ferric reductase activity |
| 78922 | scaffold_227:35786-37381 | Ferric reductase activity |
| 126175 | scaffold_27:76097-80483 | Ferric reductase activity |
| 309956 | scaffold_265:4418-6731 | Ferric reductase activity |
| 83405 | scaffold_9:69153-71527 | Ferric reductase activity |
| 130253 | scaffold_187:44081-46548 | Ferric reductase activity |

**Iron regulatory proteins.**

Phlgi1_77373 is similar to the URBS1 iron regulator, from *Ustilago maydis*. Close examination of the sequence revealed the absence of the CRR region (Cys rich region) between the two-zinc finger domains, commonly found in this group of transcriptional regulators (recently reviewed by [46]. BLAST analysis revealed close similarity to PIR1, the *P. chrysosporium* iron regulator recently described [46]. No significant transcript regulation was observed. URBS1 is known as SRE in *Neurospora crassa* and *Aspergillus*, which acts in conjunction with HapX in a complex regulatory circuit. Nevertheless, no HapX sequence was identified. This regulatory protein plays an important role in the control of iron homeostasis when this metal is in high concentrations.

**Siderophores.**

Siderophore biosynthesis shares a first common and committed step for the hydroxylation of ornithine, which is catalyzed by the ornithine mono-oxygenase Sid1/SidA, first described in *U. maydis* [47]. No putative Sid1 gene was identified. Accordingly, a gene encoding a siderophore peptide synthetase was not found. Importantly, *in silico* analysis of several white and brown-rot fungi has revealed that putative *sidA* encoding genes are present in most (but not all) white-rotters, while they are rarely found in the genomes of brown-rot basidiomycetes [38].

**Quinone reductases.**

These enzymes are associated with quinone redox cycling, thus thought to be connected to the Fenton reaction [48]. Tentative protein models include Phlgi1_18080, Phlgi1_124588, Phlgi1_81089, Phlgi1_309021 and Phlgi1_14295. Peptides and relatively high transcripts corresponding to Phlgi1_11626, another putative 1,4 benzoquinone reductase, were detected (Dataset S2). Two potential phenylalanine ammonia lyases (PAL; Phlgi1_83658, Phlgi1_352235), likely involved in biogenesis of aromatic substrates, were detected. Phlgi1_83658 showed 2-fold transcript regulation in NELP relative to ELP (p<0.05).

**Glycopeptides (potentially involved in iron reduction).**

Small molecular weight glycopeptides have also been implicated in Fenton chemistry via extracellular iron reduction [49]. Several GLP-like proteins were identified including Phlgi1_99429, Phlgi1_65475, Phlgi1_220080, Phlgi1_219719 and Phlgi1_219699. Most, if not all, featured predicted N-terminal signal peptides. Nevertheless, no peptides were detected and none of the corresponding transcripts exhibited significant regulation.

**S.1.5 Monosaccharide catabolic gene annotation and regulation**

Based on KOG categories, seventy-three gene models were verified in *P. gigantea*. The genes encoding enzymes of glycolysis were all identified. *P. gigantea* contains a single hexo- and a single glucokinase, which were identified by phylogenetic analysis together with known members from other fungi. As in *Pleurotus ostreatus* and *C. subvermispora* phosphoglycerate mutases were strongly amplified, and some genes were reminiscent of ancient duplications and horizontal gene transfer events or deletion in other fungi.

With the exception of glucose-6-phosphate dehydrogenase, the genes encoding enzymes of the pentose phosphate pathway were also partially amplified: two 6-phosphogluconate dehydrogenases, transketolases and transaldolases were found. A gluconate kinase was identified but, as in *C. subvermispora*, a glucose oxidase was not assigned by KOG. Nevertheless, close examination and alignments classified Phlgi1_128108 (Table S5) as a likely glucose oxidase. As mentioned above (GMC section), this oxidase was significantly (P<0.03) upregulated (>6-fold) in LP media relative to Glu medium, and the corresponding peptides identified by mass spectrometry.

The genes required for catabolism of other monosaccharides arising from hemicelluloses were also found: the Leloir pathway for D-galactose catabolism is present. Since this pathway exclusively requires the α-anomer, two intracellular and one putative extracellular mutarotases are also present. As in *C. subvermispora* and *P. ostreatus, P. gigantea* has two galactokinases, one which appears to be an orthologue to other basidiomycete galactokinases, and the other orthologous to ascomycetes.

We also detected two of the genes encoding the enzymes necessary for degradation of D-xylose and L-arabinose, i.e. aldose reductase, and xylitol dehydrogenase. Tests for other genes resulted in several hits with only small differences in Blast E values. Since only ascomycetous queries were available, these were only in the range of e-40 and higher, and we therefore cannot decide if these findings reflect a multiplicity of pentose catabolism genes, as in *P. ostreatus* and ascomycetes.

Thirteen of the 27 glycolytic genes acted at the C3 level of carbohydrate breakdown. Together with the finding of seven aquaporin genes this may indicate that the rapid formation and turn-over of glycerol as an osmotic stabilizer has an important function in *P. gigantea*, as also shown in *C. subvermispora*.

Beyond glucose oxidase, few genes were found to be significantly regulated. However, glycerol kinase Phlgi1_106139 was upregulated 1.9-fold (P<0.01) in NELP relative to ELP and this pattern was accompanied by glycerol permease Phlgi1_99331 (2.1-fold; P<0.014).

**S.1.6 Carbohydrate Active Enzymes (CAZys)**

A total of 306 CAZy protein modules were identified which included 197 glycoside hydrolases (GHs), 19 carbohydrate esterases (CEs) and 6 polysaccharide lyases (PLs). Classifications are tabulated in Tables S7-S9 and GH5s are further subdivided as recently recommended (Figure S22; [50].

**Lytic polysaccharide monoxygenases (LPMOs; formerly GH61s)**

Analysis of the *P. gigantea* genome revealed fifteen gene models annotated as lytic polysaccharide monoxygenases (previously classified as CAZy GH61)[51,52] (Table S10; Figure S23). Proteins Phlgi1_64774, Phlgi1_125213 and Phlgi1_79143 are represented by fragments translated from incomplete gene models. Twelve gene models are predicted to contain N-terminal secretion signal sequences that, when cleaved would yield the family conserved N-terminal histidine considered to be important for metal coordination [52,53]. One additional gene model (Phlgi1_79150) was verified through sequence alignments to have the conserved N-terminal histidine although the signal sequence prediction indicated a shifted cleavage position which would effectively remove this amino acid (Table S10). In addition to the uncertain interpretation of the N-terminus of this model the C-terminus codes a truncated CBM1 domain. While not identified using the protein domain prediction tools available through the NCBI, it was readily verified using the Pfam server directly. Amino acid alignments indicate that the last of four conserved cysteine residues is missing along with the last five amino acids. Including this model, a total of four models have C-terminal annotated CBM1 domains.

Peptides representing four LPMO gene models were detected by nano LC-MS/MS. Notably, for three of these LPMO enzymes (Phlgi1_227588, Phlgi1_227560 and Phlgi1_118444) the peptides were detected on loblolly pine (LP) for all three sampling days but only on the first sampling day for the ELP cultures (Dataset S2). Peptides corresponding to the fourth detected LPMO (Phlgi1_37310) were only observed on the first sampling of NELP cultures. This partially supports transcriptome findings for this gene model that show this transcript expressed constitutively at comparably high levels (RPKM values >1200). Two of the LPMOs detected through secretome analysis corresponded to increased transcript levels growing on ELP and NELP relative to growth on glucose. Overall, five transcripts increased significantly (p≤0.01) during growth on NELP while only four increased with growth on ELP suggesting that the extractives removed by acetone may be involved in gene regulation. Additionally, three GH61 models were expressed at significantly greater levels on NELP relative to ELP.

**Carbohydrate esterases (CEs)**

Nineteen *P. gigantea* proteins were classified into 7 CE families (CE1, 4, 8, 9, 12, 15 and 16). Phylogenetic analysis was performed for each CE family by using CE gene sequences encoded by other Agaricomycete fungi. CE family 1 includes acetyl xylan esterases, and the two CE1s in *P. gigantea* clustered with CE1 protein sequences from *P. carnosa* and *P. chrysosporium* (Figures S24 to S30). While two CE1s encoded by *P. carnosa* comprise an N-terminal CBM1, those encoded by *P. gigantea* and *P. chrysosporium* appear to lack CBMs. Similar trends were observed for the other six CE families identified in the *P. gigantea* genome sequence, where most CEs lacked a CBM and were clustered with corresponding CE families in *P. carnosa* and *P. chrysosporium*. Notable exceptions were the prediction of a CE12 in *P. gigantea*, which has not been identified in *P. carnosa* or *P. chrysosporium*, and the presence of an N-terminal CBM on one of the seven CE16 enzymes.

Differential expression of *P. gigantea* CEs between cultures was deemed insignificant when applying pre-set criteria to estimate up/down-regulated genes (RPKM values >10, ratios exceeding 2-fold up or down, and FDR probabilities <0.01). When relaxing criteria to FDR probabilities p<0.05, and ratios >2-fold regardless of RPKM values, Phlgi1_30798 and Phlgi1_487938 (both from CE family 8) were classified as up-regulated in NELP or ELP cultures (Figure S26). BLASTp analyses predict that both of these genes likely encode pectin esterase activity. Relatively few agaricomycete species encode CE12 proteins. Notably, the one CE12 predicted in *P. gigantea* is 65 % identical to a lipase from *F. mediterranea* (Fomme1_31947) and was up-regulated in control and extracted cultures compared to the glucose culture (Figure S27). Among the seven *P. gigantea* CE16 proteins, Phlgi1_103464 was up-regulated in test cultures compared to controls, and like orthologous proteins in *P. chrysosporium*, *T. versicolor* and *H. annosum,* was the only CE16 with a CBM1 domain (Figure S30).

**Regulation of CAZy gene expression**

For identification of binding sites for proteins regulating cellulase and hemicellulase induction in *P. gigantea*, all glycosyl hydrolases with transcripts accumulating >2-fold under the both conditions (*vide supra*) relative to glucose were selected, which resulted in retrieval of 10 genes. The RSA tool [54] was used to search for motifs that were abundant in 1000 5’-sequences (relative to the first coding ATG). Searches were conducted for direct motifs with a maximum of 8 nts and dyadic 3-4 nt long. Three motifs were detected (GACAGC, GTTNNNNNCGA, TGGCCA). A search of the YEASTRAC database, however, did not identify any known protein that binds to these sequences.

To identify potential binding sites for known transcription factors, the ten promoters were subjected to an analysis by YEASTRAC, which resulted in the detection of a considerable number of binding sites. We consequently selected those 10 proteins, for which binding sites were present in all or at a minimum 8 of the 10 promoters. Their properties are listed in Table S11. Although unclear in basidiomycetes, the roles of these transcription factors in yeast center on regulation in response to carbohydrate breakdown, oxygen and general stress, and unfolding protein stress (commonly occurring during phases of massive protein secretion). We therefore tested whether corresponding orthologues for these transcription factors would be present in the *P. gigantea* genome. Blast e-values were between e^-40^ and e^-25^ for most hits, which is in the range of positives between yeast and basidiomycetes because of the large evolutionary distance.

No orthologues were detected for GCR1, ASH1 and HAC1. Nevertheless, for the latter, if the *C. neoformans* HAC1/XBP1 orthologue is used as bait, gene model Phlgi1_19422 is retrieved. The *C. neoformans* HXL1 (*HAC1* and *XBP1*-*L*ike gene *1*) is the transcription factor involved in the UPR response in this organism, presenting unique structural features. Clearly, these data are preliminary but it may be interesting to test whether they are functional in this way in *P. gigantea.* Also, no binding sites for the ascomycete cellulase and xylanase regulator XYR1/XlnR (5’-GGSGAAT/AA) were found in this study, and we also could not detect a potential orthologue in the *Phlebiopsis* genome. Regulation by XYR1/XlnR seems therefore to be absent from *P. gigantea*. Finally, no binding sites for the CreA/CRE1/Mig1/2/3 carbon catabolite repressor were detected by YEASTRAC. In order to specifically test whether *Phlebiopsis* indeed contains an orthologue of MIG1/CreA/Cre1, we used orthologue of *C. subvermispora* in a BLAST search. Phlgi1_69101 was obtained as ‘best hit’ (79 % similarity a third of the whole sequence; 3.6e^-43^). A closer inspection shows, however, that this protein in *P. gigantea* was incorrectly annotated. Using a longer version of its nt sequence that theoretically should contain a full-length CRE1 orthologue, in blastx identified this gene to encode a DEAD_2 box containing DNA-repair helicase which is conserved in close phylogenetic neighbors of *P. gigantea*. Transcript levels are listed in Table S12.

**S.1.7 P450s and secondary metabolism**

**P450 annotation**

Putative P450 hits in the JGI whole genome database were subjected to annotation based on the presence of the conserved P450 signature domains namely, the oxygen-binding motif and the heme-binding motif. Sequences that showed these characteristics P450 signatures were considered as “authentic P450s” and the remaining sequences that showed one or both of the conserved domains or exhibited sequence homology to P450 proteins in general were grouped as “tentative P450s”. The remaining sequences that showed no conserved P450 domains or a convincing homology to the P450 proteins were grouped as “non-P450s” and dropped from further analyses. The authentic P450s were grouped into CYP families and subfamilies based on the existing nomenclature criteria of > 40% homology for assigning a family and > 55% for a subfamily. The families were then grouped into clans. P450 superfamily nomenclature rules set by the International P450 Nomenclature Committee were followed for assigning the clan, family and subfamily classification.

**P450ome annotation and classification.**

Cytochrome P450 monooxygnases (P450s) are heme-thiolate proteins found across the biological kingdoms. These enzymes perform a wide-variety of reactions such as hydroxylation, epoxidation, dealkylation, sulfoxydation, deamination, desulphuration, dehalogenation, and nitro reduction [55]. *Phlebiopsis gigantea* genome features 127 P450 genes (P450ome) and 9 pseudo-P450 genes (Table S13 and Figure 31S). Using the P450 nomenclature criteria, *P. gigantea* P450ome was classified into 11 clans (10 named clans and one un-named clan), 34 families and 62 subfamilies (Tables S13 and S14). Among clans, CYP64 includes highest number of member P450s (50 P450s) followed by CYP52 (21 P450s), CYP53 (12 P450s), CYP534 (10 P450s), CYP67 (8 P450s), CYP547 (7 P450s), CYP503 (6 P450s), CYP51 (5 P450s), CYP505 (4 P450s) and CYP61 (1 P450) (Table S14). Three P450s belonging to families CYP6001 (2 P450s) and CYP5108 (1 P450) were grouped under the hitherto un-named (UN) clan in the overall classification scheme for this protein superfamily. Among the 34 families, CYP5144 includes the highest number of member P450s (24 P450s) followed by CYP5150 (11 P450s) and CYP5141/CYP5037 (7 P450s each) (Table S14 and Figure S31).

**Comparative P450omics with other wood rots**

The *P. gigantea* P450ome features a P450 count somewhat lower than that reported for the model white-rot basidiomycete *P. chrysosporium* (149 P450s) [31,56] and much lower as compared to the model brown rot basidiomycete *Postia placenta* (250 P450s) [4]. Clan-level P450ome comparison between the two white rot species *P. gigantea* and *P. chrysosporium* showed the presence of a novel (hitherto un-named) clan in *P. gigantea* (Figure 32). In comparison to *P. chrysosporium* which colonizes dead wood, this fresh wood-degrading species showed large reduction in P450 count across the clans CYP64, CYP67 and CYP503 (Table S14). For clans CYP51, CYP61, CYP505 and CYP547, *P. gigantea* has the same number of P450s as *P. chrysosporium*. For clans CYP52, CYP53 and CYP534, *P. gigantea* has somewhat higher number of member P450s as compared to *P. chrysosporium* (Table S14). Family-level P450ome comparison between *P. gigantea* and *P. chrysosporium* revealed four new P450 families, namely CYP5093, CYP509, CYP6001 and CYP5108 and absence of two families viz. CYP5137 and CYP5149 in *P. gigantea* (Table S14). Interestingly, the *P. gigantea* P450ome showed 13 new subfamilies when compared to *P. chrysosporium*. However, *P. gigantea* showed a reduction in the number of subfamilies (7) when compared to *P. chrysosporium* (Table S14).

*P. gigantea* however showed a somewhat differing pattern when compared to the other recently sequenced species of *Phanerochaete*. Specifically, *P. carnosa* [32] encodes a larger contingent of P450s (266 P450s) as compared to other sequenced wood-degrading basidiomycetes. Like *P. chrysosporium*, the *P.carnosa* genome sequence did not include P450 families CYP5108, CYP5093 or CYP509, but did include family CYP6001 as well as families CYP5137 and CYP5149 (Table S14). Notably, *P. gigantea* and *P. chrysosporium* genome sequences could be distinguished from the *P. carnosa* genome sequence by the presence of the CYP503 P450 family (Figure S32). By contrast, the CYP54 family was uniquely absent from the *P. gigantea* genome (Figure S32).

**Functional significance of the P450ome in *P. gigantea***

The presence of a large repertoire of P450 monooxygenase genes when compared to other oxidoreductase genes in *P. gigantea* genome suggests a potential role of these P450s in various endogenous and xenobiotic metabolic processes. However, except for the conserved fungal P450s such as CYP51, CYP61, CYP52, and CYP505, the majority of the P450s identified in the *P. gigantea* genome are orphan with no known function. Functional analysis of the available basidiomycete P450omes has suggested that the highly conserved P450s that are common across different fungal phyla viz. CYP51 and CYP61 play roles in basic cell wall development process [57]. Other P450s which are fairly conserved across the fungal phyla viz. CYP52 and CYP505 participate in cellular metabolism of aliphatic compounds (fatty acids and alkanes). Functional genomic studies on various orphan P450s of the model white rot basidiomycete *P. chrysosporium* showed that P450s belonging to CYP families CYP63, CYP5136 and CYP5142 to CYP5145 catalyze oxidation of a range of xenobiotic structures including carcinogenic and/or mutagenic polycyclic aromatic hydrocarbons and endocrine-disrupting alkylphenols [56,58-60]. Several P450s with predicted roles in secondary metabolite biosynthesis and oxidation of xenobiotic compounds (based on known P450 homologs in other organisms) were found to be up-regulated in *P. gigantea* (Table S15). Functional analysis of the up-regulated P450s using experimental approaches will authenticate their predicted functions and role in this organism. Considering that this basidiomycete is capable of colonizing the fresh wood and up-regulation of P450s in response to a solvent extract of the wood, several of the P450s may be expected to be involved in the colonization process via degradation or assimilation of plant defense chemicals and wood extractives*.*

***Phlebiopsis gigantea* secondary metabolism**

The *Phlebiopsis gigantea* genome harbors a small but intriguing set of genes (Table S16) putatively coding for natural product biosynthesis enzymes. Two loci coding for nonribosomal peptide synthetase (NRPS)-like proteins were identified. Protein Phlgi1_66945, now referred to as Lys2, is a putative α-aminoadipate reductase and thus very likely involved in L-lysine biosynthesis, i.e., primary metabolism. This hypothesis is supported by its typical domain structure consisting of a single adenylation (A) domain, thiolation (T) and a reductase (R) domain (A-T-R). Bioinformatic analysis of its adenylation domain to extract key amino acid positions which line the active site yielded a substrate specificity code (D-P-R-Y-F-V-M-A-V) very similar to Lys2p [61] of *Candida albicans* (D-P-R-L-F-V-M-S-V). Protein Phlgi1_35145 (NPS1) exhibits a similar domain architecture (A-T-R), however, deviates in its specificity code (D-M-W-C-A-A-S-I-V), which determines an unknown amino acid substrate. Recently published data for Aspergilli show that enzymes with A-T-R domain layout do play a role in secondary metabolism [62]. We assume NPS1 contributes to natural product formation, too. Related genes coding for NPS1-like enzymes with an identical substrate specificity code are present in various lignocellulose-degrading fungi including *Ceriporiopsis subvermispora, Phlebia brevispora, Heterobasidion annosum* and *Trametes versicolor*. Thus, the presence of these genes and the capacity to produce structurally still unidentified small molecules seems to correlate with a lignocellulose-degrading lifestyle or may even enhance the fitness of wood degrading fungi in their natural environment.

Apart from NRPS genes, *P. gigantea* contains a locus coding for one polyketide synthase (PKS1, protein 36582). Though it is disrupted by two sequence gaps, it is reasonable to assume it has a typical domain of non-reducing PKS, including starter unit acyl transferase (SAT) and product template (PT) domains, canonical keto synthase (KS) and acyl transferase (AT) domains, along with a tandem acyl carrier protein (ACP) domain and a terminal thioesterase (TE) (SAT-KS-AT-PT-ACP-ACP-TE). Typically, such multi-domain enzymes produce aromatic compounds like orsellinic acid [63]. Intriguingly, we also identified genes for halogenases, i.e., tailoring enzymes of polyketide products (Phlgi1_534844 and Phlgi1_30804, respectively).

Four genes encode putative metal-dependent terpenoid synthases/prenyltransferases (Phlgi1_126738, Phlgi1_114823, Phlgi1_359064, Phlgi1_367715) and one squalene cyclase (Phlgi1_87649).

**S.1.8 Comparative analysis of hydrophobins and ABC proteins**

**Hydrophobins**

Hydrophobins are small secreted proteins approximately 100-120 aa in length with 8 conserved cysteine residues strictly arranged in a conserved motif. Hydrophobins have only been reported in filamentous fungi. They are often secreted extra-cellularly but can also be found inside fungal structures such as fruit bodies and mycelium [64]. Sequences of *P. gigantea* hydrophobins from published data [65] were used as queries to identify hydrophobins in the gene catalogue of *P. gigantea* genome v 1.0, using tBlastn and BlastP. ORFs found were used as queries for further searches to identify all possible hydrophobin proteins in *P. gigantea*. This process was repeated until no new hydrophobins were found. The blast searches identified 20 genes with hydrophobin domains. Out of the 20 proteins found, proteins Phlgi1_18178 and Phlgi1_18177 presented abnormally long C-termini. The protein sequence for Phlgi1_18177 has very long C-terminal repeats. Whether these two proteins (Phlgi1_18178 and Phlgi1_18177) are still functional is not clear, however the proteins still retain the 8 cysteine residues, a common feature for most fungal hydrophobins. Protein Phlgi1_39115 had no signal peptide while proteins Phlgi1_80816, Phlgi1_39999, Phlgi1_53256 and Phlgi1_39115 had incomplete N-termini, but alternative models containing proper N-termini were available (Table S17). Proteins Phlgi1_114166, Phlgi1_17842, Phlgi1_80088 and Phlgi1_270986 have 6 cysteine residues, a deviation from the conventional number of cysteine residues present in most fungal hydrophobins. Another protein, Phlgi1_80816 has an extra cysteine residue making it a total of 9 cysteines for this protein. Another interesting observation is the location of the hydrophobin encoding genes in the *P. gigantea* genome. Most of the genes clustered together in the genome. For example, 5 of the genes were located on scaffold 8, 4 genes on scaffold 46, and 2 genes on scaffolds 6, 13 and 407 respectively. The biological relevance of such clustering is not known, however it is possible that the arrangement could be evidence of duplication events. All the protein sequences identified in this inventory have been manually curated, more specific details about each protein can be found in Table S17. Multiple alignments of the protein sequences identified the conserved cysteine residues specific for fungal hydrophobins (Figure S33). Comparison of the protein sequences with other published consensus sequences of class I C-X_5-7_-C-C-X_19-39_-C-X-_8-23_-C-X_5_-C-C-X_6-18_-C-x-_2-13_ [65,66] and class II C-X_9_-C-C-X_11_-C-X_14_-_16_-C-X_8_-C-C-X_10_-C-x-_6-7_ [65,67] proteins showed that all the identified hydrophobins in *P. gigantea* are class I proteins.

Phylogenetic analysis of 14 hydrophobin sequences from *P. gigantea* and 160 hydrophobin sequences from 10 closely related basidiomycetes and one ascomycete (Table S18) showed 5 distinct clusters (Figure S34), although with very low support. The hydrophobins from *P. gigantea* shared the first two clusters with hydrophobins from *P. chrysosporium*, *C. subvermispora*, *Ganoderma sp., Phlebia brevispora, Serpula lacrymans* and *Wolfiporia cocos* (Figure S33). Notably, most of those species belong to the order Polyporales (except for *S. lacrymans*). This trend indicates a close evolutionary relationship between members of hydrophobin gene families from these fungal species. It is also evident from the phylogenetic tree that different hydrophobins from *P.gigantea* evolved differently from other members of the gene family with some members sharing closer evolutionary relationship with orthologues from other fungal species than with the members from the same gene family. Transcriptome analysis of the hydrophobins from *P. gigantea* revealed differential expression but generally low transcript levels under the conditions tested (Table S19; Dataset S2).

**Comparative analysis of *P. gigantea* ABC proteins**

Based on the comparison of *P. gigantea* ABC proteins with homologues from other basidiomycetes, a total of 48 genes encoding putative ABC proteins were detected in the genome of *P. gigantea* (Table S20; Dataset S2). A large fraction of the identified proteins (39 out of 48) feature predicted transmembrane domain(s), and are therefore likely to be involved in the transport of certain substrates across the membrane. The remaining 9 ABC proteins apparently lack transmembrane domains, and for 7 of them cellular functions not related to the transport can be predicted based on homology with known proteins from other fungi, mainly *Saccharomyces cerevisiae*. Two ABC proteins without transmembrane domains show some similarity to the components of bacterial multisubunit ABC transporters; however no corresponding transmembrane components could be identified so far, and their biological role in the fungi remains unclear.

Overall, the *P. gigantea* set of ABC proteins shows high similarity to the one of *P. chrysosporium*. A total of 50 predicted ABC proteins were identified in the genome of *P. chrysosporium*. The two sets are different in 6 proteins. Two of *P. gigantea* proteins were not found in the genome of *P. chrysosporium*, while 4 of *Phanerochaete* ABC transporters are apparently missing from the genome of *Phlebiopsis*. These numbers are rather low, taking into account the rapid evolution of the members of this protein family within fungi.

Here, we briefly discuss the distinctive features of *P. gigantea* ABC proteins set, following the accepted subfamily classification. Most of the data on ABC transporters in other fungal species, particularly in ascomycetes, were taken from a previous publication [68].

**Subfamily A**. We could not identify any genes coding for ABC-A transporters in the genome of *P. gigantea*. This is rather unusual, as the corresponding genes were found in most of the analyzed species of *Agaricomycotina*, in *Ustilago maydis* and in most of *Pezizomycotina*. On the other hand, those genes are missing from the genomes of many yeast-like fungi, e.g. *Saccharomyces cerevisiae*, *Candida albicans*, *Schizosaccharomyces pombe* and *Cryptococcus neoformans*, to name just a few. As *P. chrysosporium* also lacks the corresponding gene, we assume that it was lost in the *Phlebiopsis*/*Phanerochaete* lineage. Interestingly, ABC-A transporter of rice pathogen *Magnaporthe grisea* is required for pathogenesis and appresoria formation [69], but no data are available on the function of its homologues in basidiomycetes.

**Subfamily B.** The set of *P. gigantea* ABC-B proteins has much in common with other *Agaricomycotina*. It includes 3 full-length transporters and 9 so-called half-transporters. They can be readily assigned to the groups identified in previous analysis. So, the *P. gigantea* full-length ABC-B transporter (Phlgi1_34158) is homologous to the *S. cerevisiae* a-factor exporter Ste6p, while two others (Phlgi1_127165 and Phlgi1_129050) belong to the group of proteins related to the *S. pombe* multidrug-resistance transporter Pmd1. Three of the *P. gigantea* half-transporters also belong to the highly conserved groups widely distributed among fungi: mitochondrial Fe/S cluster precursor transporters homologous to *S. cerevisiae* Atm1p (Phlgi1_91112), *S. pombe* hmt1-related transporters with a predicted role in heavy metal resistance (Phlgi1_90887) and mitochondrial peptide exporters exemplified by *S. cerevisiae* Mdl1p (Phlgi1_87882). The remaining 6 half-transporters (Phlgi1_21134, Phlgi1_25601, Phlgi1_27132, Phlgi1_32454, Phlgi1_98346 and Phlgi1_111230), however, were placed into a peculiar group of ABC proteins that appears to be specific for the members of *Agaricomycotina*. To our knowledge, nothing is currently known about their biological function. The only difference between sets of ABC-B proteins in *P. gigantea* and *P. chrysosporium* is the number of the transporters belonging to the last described group, as *Phanerochaete* has only 5 members.

**Subfamily C.** This subfamily is by far the largest one among *P. gigantea* ABC proteins as it includes 19 predicted proteins. They can be further subdivided into 5 smaller groups defined in previous study. Unfortunately, the information available for three of those groups is rather scarce. Members of groups C(II) and C(III) are widely distributed among ascomycetes and basidiomycetes, but at the same time they are missing from the genomes of hemiascomycetous yeasts (including *S. cerevisiae*) and *S. pombe*. Owing mainly to this fact, none of the fungal proteins belonging to these groups was functionally characterized yet, and even their intracellular localization remains obscure. Interestingly, one of *P. gigantea* C(II) transporters (Phlgi1_106557) does not have direct counterparts in the analyzed genomes of other polyporoid fungi, and its next relatives were found in *Cryptococcus neoformans*.

The group C(IV) definitely deserves special attention. Our study revealed that its members are universally present in all analyzed species of *Agaricomycotina*. They were found also in a few ascomycetes including *S. pombe*. Based on the data obtained on *S. pombe* transporters, localization to the vacuolar membrane can be suggested for other proteins. While genomes of most fungi encode only 1 or 2 transporters belonging to this group, it has undergone a remarkable amplification in polypores: *P. placenta* has 8 members and both *Phanerochaete* and *Phlebiopsis* – as many as *­*11 corresponding genes. Phylogenetic analysis indicates that gene amplification within this group continued even after separation of lineages leading to *P. gigantea* and *P. chrysosporium*. The significance of this extensive amplification for the polyporoid fungi is not entirely clear.

Similar to most other basidiomycetes, *Phlebiopsis* has a single protein homologous to *S. cerevisiae* Ycf1p (group C(VI)) (Phlgi1_128871). Characterized members of this group are vacuolar transporters involved in the detoxification of heavy metals by mediating the accumulation of phytochelatin and glutathione in vacuoles [70]. Finally, there are three transporters (Phlgi1_14797, Phlgi1_22983 and Phlgi1_114868) related to *S. cerevisiae* Yor1p (group C(VII)), a plasma membrane-localized transporter. The last group of proteins is distinctly more abundant in *Agaricomycotina* where their numbers vary between 2 and 5 per genome than in ascomycetes, with a single gene in most of the analyzed species.

Direct comparison of sets of ABC-C transporters from *Phlebiopsis* and *Phanerochaete* shows their high similarity. The only noteworthy difference is an existence of the *P. gigantea* protein Phlgi1_106557, which does not seem to have a direct counterpart in *Phanerochaete* genome. The other thing that should be taken into account is the fact that even if the number of the transporters belonging to the group C(IV) in both species is equal, not all of them can be assigned to orthologous pairs due to the fact that gene amplification within this group apparently continued after the divergence of lineages leading to those two species.

**Subfamily D**. This group includes ABC transporters localized to the membranes of microbodies (peroxisomes). Its organization in fungi is highly conserved, and genomes of all analyzed basidiomycetes encode two half-transporters belonging to this subfamily. The same situation is observed in *P. gigantea* (Phlgi1_24318 and Phlgi1_28761).

**Subfamily E.** Again, this family of proteins is highly conserved. In the genomes of all analyzed basidiomycetes and ascomycetes, there is a single gene encoding an essential protein involved in ribosome biogenesis and translation initiation. In the case of *P. gigantea*, that is protein Phlgi1_192020.

**Subfamily F.** Like the members of the previous subfamily, ABC-F proteins are not involved in the transmembrane transport. Instead, they act as translational elongation factors or play important roles in ribosome biogenesis, translational control and mRNA export. Most of the analyzed fungal genomes carry either 4 or 5 genes encoding ABC-F proteins that can be further subdivided into 5 homology groups. Likewise, *P. gigantea* has 5 ABC-F proteins but, interestingly, we could not find a protein belonging to the subgroup F(III). At the same time, there are two proteins of the subgroup F(V). The same pattern is observed in *P. chrysosporium* and *H. annosum*, but our preliminary phylogenetic analysis indicates that those duplications probably occurred independently in all three species. The loss of the single member of the subgroup F(III), however, seems to be common for polyporoid fungi, as it is also missing from the genome of *Postia placenta*, while *C. cinerea*, *L. bicolor* and *S. lacrymans* retained it.

**Subfamily G.** Members of this group are also known as PDR (pleiotropic drug resistance) transporters. Six ABC-G proteins were identified in the genome of *P. gigantea*. Two of them (Phlgi1_102023 and Phlgi1_130987) belong to the group G(II) encompassing proteins related to the intensively studied yeast PDR transporters (Snq2p, Pdr11p, Pdr12p, Pdr18p and Aus1p). Remaining *P. gigantea* ABC-G proteins belong to the widely distributed but less-understood subgroups G(IV) (ID 114337), G(VI) (ID 95544) and G(VII) (ID’s 101348 and 493857). This situation reflects the general trend towards the lower number of ABC-G proteins in basidiomycetes compared with ascomycetes. The sets of ABC-G transporters in the genomes of *P. gigantea* and *P. chrysosporium* differ by 4 proteins. *Phanerochaete* carries one additional G(II) protein and three G(VI) proteins without counterparts in *P. gigantea* genome. Of particular interest, an abovementioned ABC efflux transporter (Phlgi1_130987) is most closely related to the *GcABC-G1* gene of the ascomycete *Grosmannia clavigera*, a pathogen of lodgepole pine [71]. The *GcACC-G1* gene appears to be a key element against the host defenses. The *P. gigantea* homolog is upregulated >4.9-fold (P=0.02) in NELF relative to ELF media (Dataset S2).

**Unclassified ABC proteins.** A small number of ABC proteins that do not fall into any of the above groups are also present in fungal genomes. One of them is homologous to *S. cerevisiae* Caf16p. The corresponding gene is present in a single copy in the genomes of all analyzed basidiomycetes and ascomycetes. In the case of *P. gigantea,* it is Phlgi1_101292. It is a part of CCR4-NOT transcriptional regulatory complex. Two remaining *P. gigantea* ABC proteins show some similarity to the components of bacterial multisubunit ABC transporters. One of these, Phlgi1_103495, is homologous to *S. cerevisiae* YDR061W. This protein with unknown function is present in virtually all analyzed basidiomycetes and ascomycetes. A second Phlgi1_102729 belongs to the group specific for the species of *Agaricomycotina*. Its function also remains unknown.

**Summary of ABC proteins**

With 48 identified members, the number of ABC genes in *Phlebiopsis* stands on the higher end compared to other analyzed basidiomycetes, as it exceeds the numbers observed in *C. cinerea*, *S. lacrymans*, *P. placenta*, *H. annosum*, *C. neoformans*, *U. maydis* and *P. graminis*. The relatively high number of *Phlebiopsis* ABC proteins owes to the amplification of the single group of ABC transporters, namely C(IV), as number of proteins belonging to the remaining groups does not differ significantly from other species.

The set of *P. gigantea* ABC proteins shows high similarity to those of *P. chrysosporium*. Their common features include the loss of ABC-A and ABC-F(III) genes and the proliferation of ABC-C(IV) group.

Another feature that seems to be common for the broad range of basidiomycetes is the relatively low number of ABC-G and full-length ABC-B transporters combined with the higher number of ABC-C proteins as compared with ascomycetes. Members of those three subfamilies seem to take different evolutionary routes in the large fungal phyla. Some of the groups that are abundant in the genomes of ascomycetes are totally missing from higher basidiomycetes, e.g. ABC-B(II), ABC-B(III), ABC-C(I), ABC-G(III) and ABC-G(V), while the group ABC-G(I) is represented only by a single gene in *H. annosum*. Interestingly, at least some of those groups are still present in the genome of *C. neoformans*, belonging to the lineage that separated early in the evolution of *Agaricomycotina*. On the other hand, members of the group ABC-C(IV) were found only in few ascomycetes while it is common (and sometimes very abundant) in all analyzed *Agaricomycotina*. Finally, two groups of ABC proteins seem to be specific either for mushroom-forming fungi (a group of ABC-B half-transporters related to *C. cinerea* CC1G_00632) or for *Agaricomycotina* (proteins related to *C. cinerea* CC1G_04237).

It is tempting to speculate that the evolution of fungal ABC proteins within the discussed phyla was driven by the fundamental differences in their life style and life forms. Most of the ascomycetes included in our analysis generally exist as simple hyphae and do not form massive multi-layered structures. Therefore, they can rely on the extracellular export of toxic compounds as a principle way of their utilization.

On the other hand, mushroom-forming basidiomycetes develop massive fruiting bodies consisting of numerous cell layers. In case of polyporoid fungi, those fruit bodies are often long-lived and continue to grow for several years. Within the fruiting body, the excretion of toxic compounds outside of the cell, the function generally attributed to many of the ABC transporters, would not help to get rid of them, as they will remain and eventually accumulate in the intercellular space. In such situation, the sequestration within a vacuole might become a preferred way for the detoxification of unwanted compounds. This eventually could lead to the decrease in number of plasma membrane transporters accompanied by the simultaneous expansion and diversification of vacuolar transporters. All known vacuolar ABC transporters in fungi belong to the subfamily ABC-C, while ABC-G and full-length ABC-B transporters usually localize to plasma membrane. Therefore, we reasoned that development of massive fruiting bodies in basidiomycetes could be one of the driving forces for the evolution of ABC transporters within this group.

**S.1.9 RNAseq and LC-MS/MS methods**

**RNAseq.** Mycelium was derived from triplicate cultures of 250 ml basal salts containing: 1. 1.25 g freshly-harvested, ground (1mm mesh) loblolly pine that had been ‘spiked’ with acetone and thoroughly dried (NELP); or 2. The same material following extended soxhlet extraction and drying (ELP). Duplicate cultures of basal salts medium with glucose as sole carbon source also served as a reference. After 5 days incubation, total RNA was purified from frozen mycelium as described [5,72]. The Illumina TruSeq protocol was used to prepare samples for multiplexed HiSeq2000 sequencing. Briefly, total RNA samples were tested at the University of Wisconsin-Madison Biotechnology Center for quality control (NanoDrop Spectrophotometer and Agilent 2100 BioAnalyzer for purity and integrity verification, respectively). For RNA-Seq library preparation, each library was generated using Illumina’s “TruSeq RNA Sample Preparation Guide” (Illumina Part # 15008136, Rev. A, August 2011) and the Illumina TruSeq RNA Sample Preparation Kit (Illumina Inc., San Diego, California, USA). Messenger RNA was purified from 1μg total RNA using poly-T oligo-attached magnetic beads. Double-stranded cDNAs were synthesized using SuperScript II (Invitrogen, Carlsbad, California, USA) and random primers for first strand cDNA synthesis followed by second strand synthesis using DNA polymerase I and RNAse H for removal of mRNA. Double-stranded cDNA was purified using Agencourt AMPure XP beads (Qiagen, Valencia, California, USA) as recommended in the TruSeq RNA Sample Prep Guide. cDNAs were end-repaired by T4 DNA polymerase and Klenow DNA polymerase and phosphorylated with T4 polynucleotide kinase. The blunt ended cDNA was purified using Agencourt AMPure XP beads. The cDNA products were incubated with Klenow DNA polymerase to add an ‘A’ base (Adenine) to the 3’ end of the blunt phosphorylated DNA fragments and then purified using Agencourt AMPure XP beads. DNA fragments were ligated to Illumina adapters having a single ‘T’ base (Thymine) overhang at their 3’end. The adapter-ligated products were purified using Agencourt AMPure XP beads. Adapter ligated DNA was amplified in a Linker Mediated PCR reaction (LM-PCR) for 15 cycles using Phusion^TM^ DNA polymerase and Illumina's PE genomic DNA primer set, then purified using Agencourt AMPure XP beads. Quality and quantity of the finished libraries were assessed using an Agilent DNA1000 series chip assay (Agilent Technologies, Santa Clara, CA) and Invitrogen Qubit HS Kit (Invitrogen, Carlsbad, California, USA), respectively, and libraries standardized to 2 μM. Cluster generation was performed using a TruSeq Single Read Cluster Kit (v3) and the Illumina cBot, with libraries multiplexed in a single HiSeq2000 lane. Images were analyzed using CASAVA version 1.8.2 and FASTQ files generated. DNAStar Inc (Madison, WI) modules SeqNGen and Qseq were used for mapping reads and statistical analysis. Transcriptome data was deposited to the NCBI Gene Expression Omnibus (GEO) database (<http://www.ncbi.nlm.nih.gov/geo/>) and assigned accession GSE53112.

**LC-MS/MS secretome analysis**

With minor modification, NanoLC-MS/MS analysis identified extracellular proteins in culture filtrates as described [5,73]. For each of the two woody substrates (e.g NELP and ELP), cultures were harvested after 5, 7, and 9 days. Cultures containing microcrystalline cellulose (Avicel) were prepared as previously described [74,75], except that the cellulose was first suspended in acetone or in the pine acetone extract and then thoroughly dried under vacuum in a Rotovap apparatus. The volume of pine extract was adjusted to approximate compositional analysis (~56 mg per g dry weight; see below)

“In Liquid” digestion and mass spectrometric analysis was done at the Mass Spectrometry Facility [Biotechnology Center, University of Wisconsin-Madison]. In short, ~300µg of TCA/acetone precipitated extracellular protein lysates were re-solubilized and denatured in 30μl of 8M Urea / 50mM NH_4_HCO_3_ (pH8.5) for 10 min then diluted to 200μl for tryptic digestion with: 10μl of 25mM DTT, 14μl MeOH, 1μl 1M Tris-HCl (pH7.5), 115μl 25mM NH_4_HCO_3_ (pH8.5) and 30μl of trypsin solution [100ng/μl *Trypsin Gold* from PROMEGA Corp. in 25mM NH_4_HCO_3_]. Digestion was conducted for 2 h at 42°C then additional 15µl of trypsin solution added [final enzyme:substrate 1:67] and digestion proceeded overnight at 37°C. Reactions were terminated by acidification with 2.5% TFA [Trifluoroacetic Acid] to 0.3% final and 6μl (~7.5µg) loaded for nanoLC-MS/MS analysis.

Peptides were analyzed by nanoLC-MS/MS using the Agilent 1100 nanoflow system (Agilent Technologies) connected to a hybrid linear ion trap-orbitrap mass spectrometer (LTQ-Orbitrap XL, Thermo Fisher Scientific) equipped with a nanoelectrospray ion source. HPLC was performed using an in-house fabricated 15-cm C18 column packed with MAGIC C18AQ 3µm particles (MICHROM Bioresources Inc., Auburn, CA) and laser pulled tip (P-2000, Sutter Instrument) using 360µm x 75µm fused silica tubing. Sample loading (6µl) and desalting were done at 10µL/min using a trapping column in line with the autosampler (Zorbax 300SB-C18, 5µM, 5x0.3mm, Agilent Technologies). Peptide elution used solvents comprised of 0.1% formic acid in water (solvent A) and 0.1% formic acid, 95% acetonitrile in water (solvent B). The gradient consisted of a 20 min loading and desalting period with column equilibration at 1% solvent B, an increase to 40% B over 195 min, ramp to 60% B over 20 min, increase to 100% B in 5 min and hold for 3 min. The column was then re-equilibrated at 1% B for 30 min. The flow rate for peptide elution and re-equilibration was at 200 nl/min. The LTQ-Orbitrap was set to acquire MS/MS spectra in data-dependent mode as follows: MS survey scans from m/z 300 to 2000 were collected in centroid mode at a resolving power of 100,000. MS/MS spectra were collected on the five most-abundant signals in each survey scan. Dynamic exclusion was employed to increase dynamic range and maximize peptide identifications. This feature excluded precursors up to 0.55 m/z below and 1.05 m/z above previously selected precursors. Precursors remained on the exclusion list for 40 sec. Singly-charged ions and ions for which the charge state could not be assigned were rejected from consideration for MS/MS. Raw MS/MS data was searched against *P. gigantea* CR5-6 amino acid sequence database (11,891 protein entries) using in-house *Mascot* search engine 2.2.07 [Matrix Science] with variable methionine oxidation with asparagine and glutamine deamidation. Peptide mass tolerance was set at 15 ppm and fragment mass at 0.6 Da. Protein annotations, significance of identification and spectral based quantification was done with help of Scaffold software (version 3.6.3, Proteome Software Inc., Portland, OR). Protein identifications were accepted if they could be established at greater than 95.0% probability within 0.9% False Discovery Rate and contained at least two identified peptides. Protein probabilities were assigned by the Protein Prophet algorithm [76]. Proteins that contained similar peptides and could not be differentiated based on MS/MS analysis alone were grouped to satisfy the principles of parsimony. Approximate protein abundance in each of the cultures was expressed as the number of unique peptide and the exponentially modified protein abundance index (emPAI) value [77].

Avicel samples were prepared in a similar manner with a slight augmentation to digestion conditions and upgraded instrumental acquisition. In short, ~200µg of TCA/acetone precipitated extracellular protein lysates were re-solubilized and denatured in 30 μl of 8M Urea / 50mM NH_4_HCO_3_ (pH8.5) / 1mM TrisHCl for 5 minutes then diluted to 120μl for reduction/alkylation step with: 5μl of 25mM DTT, 10μl MeOH, 75μl 25mM NH_4_HCO_3_ (pH8.5). Incubated at 56°C for 15 minutes, cooled on ice to room temperature then 6μl of 55mM IAA was added and incubated in darkness at room temperature for 15 minutes, reaction was quenched by adding 16μl of 25mM DTT. Subsequently 30μl of trypsin/LysC solution mix [100ng/μl *Trypsin/LysC Mix* from PROMEGA Corp. in 25mM NH_4_HCO_3_] and 28μl of 25mM NH_4_HCO_3_ (pH8.5) was added to 200µl final volume. Digestion was conducted for 2 hours at 42°C then additional 15µl of trypsin/LysC solution was added [final enzyme:substrate ~1:44 with estimated ~200µg substrate] and digestion proceeded o/n at 37°C. Reaction was terminated by acidification with 2.5% TFA [Trifluoroacetic Acid] to 0.3% final. 50µg of resulted peptide digests were cleaned up using OMIX C18 SPE cartridges (Agilent, Palo Alto, CA) per manufacturer protocol and eluted in 20µl of 60/40/0.1% ACN/H_2_O/TFA, dried to completion in the speed-vac and finally reconstituted in 100µl of 0.1% formic acid, 4µl (~2µg) was loaded onto the instrument.

Peptides were analyzed by nanoLC-MS/MS using the nanoAcquity UPLC (Waters) connected to a tribrid quadropole / linear ion trap / Orbitrap mass spectrometer (Orbitrap Fusion, Thermo). HPLC was performed at 60°C using an ‘in-house’ fabricated 35-cm C18 column packed with 1.7 μm diameter, 130 Å pore size, Bridged Ethylene Hybrid C18 particles (Waters) and laser pulled tip (P-2000, Sutter Instrument) using 360µm x 75µm fused silica tubing. NanoHPLC system delivered solvents A: 0.2% (v/v) formic acid and 5% (v/v) DMSO in water, and B: 0.2% (v/v) formic acid and 5% (v/v) DMSO in acetonitrile. Samples were loaded onto the column for 12 min at 0.35 μl/min. Mobile phase B increases to 4% in the first 0.1 min then to 12% B at 32 min, 22% B at 60 min, and 30% B at 70 min, followed by a 5 min wash at 70% B and a 20 min re-equilibration at 0%B. The Fusion Orbitrap was set to acquire MS/MS spectra in data-dependent mode as follows: MS survey scans from m/z 300 to 1500 were collected in profile mode at a resolving power of 60,000 (at 200 *m*/*z*) with a 5 × 10^5^ ion count target. Tandem MS was performed by isolation at 0.7 Th with the quadrupole, HCD fragmentation with normalized collision energy of 30, and rapid scan MS analysis in the ion trap. The MS^2^ ion count target was set to 10^4^ and the max injection time was 35 ms. Only those precursors with charge state 2–6 were sampled for MS^2^. Dynamic exclusion was employed to increase dynamic range and maximize peptide identifications. This feature excluded precursors within a 10 ppm tolerance around the selected precursor and its isotopes. Precursors remained on the exclusion list for 45 sec. Singly-charged ions and ions for which the charge state could not be assigned were rejected from consideration for MS/MS. Raw data files were converted to the Mascot generic format (mgf) through open source format MS Convert tool using the Proteome Wizard software suite. Generated mgf files were searched using ‘in-house’ *Mascot* search engine 2.2.07 [Matrix Science] against *P. gigantea* CR5-6 amino acid sequence database (11,891 protein entries) with fixed carbamidomethylation and variable methionine oxidation plus asparagine and glutamine deamidation. Peptide mass tolerance was set at 15 ppm and fragment mass at 0.6 Da.. The entire downstream Scaffold-based analysis was conducted the same as for the previous samples.

**S.1.10 Analysis of pine acetone extract**

The acetone extract from Loblolly pine (5.6 % o.d. wood) was analyzed by GC-MS after silylation with bis(trimethylsilyl)trifluoroacetamide (BSTFA). The GC–MS analyses were performed with a Varian 3800 chromatograph coupled to an ion-trap detector (Varian 4000) using a medium-length fused-silica DB-5HT capillary column (12 m x 0.25 mm internal diameter, 0.1 µm film thickness) from J&W Scientific, enabling simultaneous elution of the different lipid classes [78]. The temperature program started at 120 °C (1 min), raised to 250 °C at 5 °C min^-1^ and then raised to 380 °C at 15 °C min^-1^, and held for 5 min. The transfer line was kept at 300 °C, the injector was programmed from 120 °C (0.1 min) to 380 °C at 200 °C min^-1^, and helium was used as carrier gas at a rate of 2 mL min^-1^. Compounds were identified by mass fragmentography, and by comparing their mass spectra with those of the Wiley and NIST libraries and standards, and quantification was obtained from total-ion peak area, using response factors of the same or similar compounds (palmitic acid, abietic acid, 1-monomyristin, sitosterol, 1,3-dipalmitin, cholesteryl palmitate and tripalmitin)(Table S21 and Figure S35).

**Additional References for Supplemental Information**

1. Parkhomchuk D, Borodina T, Amstislavskiy V, Banaru M, Hallen L, et al. (2009) Transcriptome analysis by strand-specific sequencing of complementary DNA. Nucleic Acids Res 37: e123.

2. Martin J, Bruno VM, Fang Z, Meng X, Blow M, et al. (2010) Rnnotator: an automated *de novo* transcriptome assembly pipeline from stranded RNA-Seq reads. BMC Genomics 11: 663.

3. Stammers DK, Ren J, Leslie K, Nichols CE, Lamb HK, et al. (2001) The structure of the negative transcriptional regulator NmrA reveals a structural superfamily which includes the short-chain dehydrogenase/reductases. EMBO J 20: 6619-6626.

4. Martinez D, Challacombe J, Morgenstern I, Hibbett D, Schmoll M, et al. (2009) Genome, transcriptome, and secretome analysis of wood decay fungus *Postia placenta* supports unique mechanisms of lignocellulose conversion. Proc Natl Acad Sci U S A 106: 1954-1959.

5. Fernandez-Fueyo E, Ruiz-Duenas FJ, Ferreira P, Floudas D, Hibbett DS, et al. (2012) Comparative genomics of *Ceriporiopsis subvermispora* and *Phanerochaete chrysosporium* provide insight into selective ligninolysis. Proc Natl Acad Sci U S A 109: 5458-5463.

6. Casselton LA, Olesnicky NS (1998) Molecular genetics of mating recognition in basidiomycete fungi. Microbiol Mol Biol Rev 62: 55-70.

7. Kües U, James T, Heitman J (2011) Mating type in Basidiomycetes: Unipolar, bipolar and tetrapolar patterns of sexuality. In: Pöggeler S, Wöstemeyer J, editors. Evolutions of fungi and fungal-like organisms. Berlin: Springer Verlag. pp. 97-160.

8. Korhonen K, Kauppila P (1988) The sexuality of *Phlebiopsis gigantea*. Karstenia 27: 23-30.

9. Grillo R, Hantula J, Korhonen K (2005) Interfertility between North American and European strains of *Phlebiopsis gigantea*. For Pathol 35: 173-182.

10. James TY, Sun S, Li W, Heitman J, Kuo HC, et al. (2013) Polyporales genomes reveal the genetic architecture underlying tetrapolar and bipolar mating systems. Mycologia 105: 1374-1390.

11. Kües U. Mating type genes as master regulators of mushroom development; 2013 August 26-29; Bejing. pp. 33-59.

12. Spudich EN, Jung T (2005) Microbial Rhodopsins. In: Briggs, Spudich EN, editors. Handbook of Photosensory receptors. Weinheim: Wiley-VCH.

13. Bieszke JA, Spudich EN, Scott KL, Borkovich KA, Spudich JL (1999) A eukaryotic protein, NOP-1, binds retinal to form an archaeal rhodopsin-like photochemically reactive pigment. Biochemistry 38: 14138-14145.

14. Corrochano LM, Garre V (2010) Photobiology in the Zygomycota: multiple photoreceptor genes for complex responses to light. Fungal Genet Biol 47: 893-899.

15. Shao S, Hegde RS (2011) A calmodulin-dependent translocation pathway for small secretory proteins. Cell 147: 1576-1588.

16. Ruiz-Duenas FJ, Morales M, Garcia E, Miki Y, Martinez MJ, et al. (2009) Substrate oxidation sites in versatile peroxidase and other basidiomycete peroxidases. J Exp Bot 60: 441-452.

17. Miki Y, Calvino FR, Pogni R, Giansanti S, Ruiz-Duenas FJ, et al. (2011) Crystallographic, kinetic, and spectroscopic study of the first ligninolytic peroxidase presenting a catalytic tyrosine. J Biol Chem 286: 15525-15534.

18. Piontek K, Ullrich R, Liers C, Diederichs K, Plattner DA, et al. (2010) Crystallization of a 45 kDa peroxygenase/peroxidase from the mushroom *Agrocybe aegerita* and structure determination by SAD utilizing only the haem iron. Acta Crystallogr Sect F Struct Biol Cryst Commun 66: 693-698.

19. Bordoli L, Kiefer F, Arnold K, Benkert P, Battey J, et al. (2009) Protein structure homology modeling using SWISS-MODEL workspace. Nat Protoc 4: 1-13.

20. Aranda E, Ullrich R, Hofrichter M (2010) Conversion of polycyclic aromatic hydrocarbons, methyl naphthalenes and dibenzofuran by two fungal peroxygenases. Biodegradation 21: 267-281.

21. Gutierrez A, Babot ED, Ullrich R, Hofrichter M, Martinez AT, et al. (2011) Regioselective oxygenation of fatty acids, fatty alcohols and other aliphatic compounds by a basidiomycete heme-thiolate peroxidase. Arch Biochem Biophys 514: 33-43.

22. Strittmatter E, Liers C, Ullrich R, Wachter S, Hofrichter M, et al. (2013) First crystal structure of a fungal high-redox potential dye-decolorizing peroxidase: substrate interaction sites and long-range electron transfer. J Biol Chem 288: 4095-4102.

23. Ullrich R, Hofrichter M (2005) The haloperoxidase of the agaric fungus *Agrocybe aegerita* hydroxylates toluene and naphthalene. FEBS Lett 579: 6247-6250.

24. Ullrich R, Nüske J, Scheibner K, Spantzel J, Hofrichter M (2004) Novel haloperoxidase from the agaric basidiomycete *Agrocybe aegerita* oxidizes aryl alcohols and aldehydes. Appl Environ Microbiol 70: 4575-4581.

25. Blodig W, Smith AT, Doyle WA, Piontek K (2001) Crystal structures of pristine and oxidatively processed lignin peroxidase expressed in *Escherichia coli* and of the W171F variant that eliminates the redox active tryptophan 171. Implications for the reaction mechanism. J Mol Biol 305: 851-861.

26. Floudas D, Binder M, Riley R, Barry K, Blanchette RA, et al. (2012) The Paleozoic origin of enzymatic lignin decomposition reconstructed from 31 fungal genomes. Science 336: 1715-1719.

27. Liers C, Bobeth C, Pecyna M, Ullrich R, Hofrichter M (2010) DyP-like peroxidases of the jelly fungus *Auricularia auricula-judae* oxidize nonphenolic lignin model compounds and high-redox potential dyes. Appl Microbiol Biotechnol 85: 1869-1879.

28. Whittaker MM, Kersten PJ, Cullen D, Whittaker JW (1999) Identification of catalytic residues in glyoxal oxidase by targeted mutagenesis. J Biol Chem 274: 36226-36232.

29. Vanden Wymelenberg A, Sabat G, Mozuch MD, Kersten P, Cullen D, et al. (2006) Structure, organization, and transcriptional regulation of a family of copper radical oxidase genes in the lignin-degrading basidiomycete *Phanerochaete chrysosporium*. Appl Environ Microbiol 72: 4871-4877.

30. Hoegger PJ, Kilaru S, James TY, Thacker JR, Kües U (2006) Phylogenetic comparison and classification of laccase and related multicopper oxidase protein sequences. FEBS J 273: 2308-2326.

31. Martinez D, Larrondo LF, Putnam N, Sollewijn Gelpke MD, Huang K, et al. (2004) Genome sequence of the lignocellulose degrading fungus *Phanerochaete chrysosporium* strain RP78. Nat Biotechnol 22: 695-700.

32. Suzuki H, MacDonald J, Syed K, Salamov A, Hori C, et al. (2012) Comparative genomics of the white-rot fungi, *Phanerochaete carnosa* and *P. chrysosporium*, to elucidate the genetic basis of the distinct wood types they colonize. BMC Genomics 13: 444.

33. Larrondo L, Salas L, Melo F, Vicuna R, Cullen D (2003) A novel extracellular multicopper oxidase from *Phanerochaete chrysosporium* with ferroxidase activity. Appl Environ Microbiol 69: 6257-6263.

34. Rodriguez-Rincon F, Suarez A, Lucas M, Larrondo LF, de la Rubia T, et al. (2010) Molecular and structural modeling of the *Phanerochaete flavido-alba* extracellular laccase reveals its ferroxidase structure. Arch Microbiol 192: 883-892.

35. Larrondo LF, Canessa P, Melo F, Polanco R, Vicuna R (2007) Cloning and characterization of the genes encoding the high-affinity iron-uptake protein complex Fet3/Ftr1 in the basidiomycete *Phanerochaete chrysosporium*. Microbiology 153: 1772-1780.

36. De Silva DM, Askwith CC, Eide D, Kaplan J (1995) The FET3 gene product required for high affinity iron transport in yeast is a cell surface ferroxidase. J Biol Chem 270: 1098-1101.

37. Kües U, Rühl M (2011) Multiple multi-copper oxidase gene families in basidiomycetes - what for? Curr Genomics 12: 72-94.

38. Canessa P, Larrondo LF (2013) Environmental responses and the control of iron homeostasis in fungal systems. Appl Microbiol Biotechnol 97: 939-955.

39. Bonaccorsi di Patti MC, Felice MR, Camuti AP, Lania A, Musci G (2000) The essential role of Glu-185 and Tyr-354 residues in the ferroxidase activity of *Saccharomyces cerevisiae* Fet3. FEBS Lett 472: 283-286.

40. Wang TP, Quintanar L, Severance S, Solomon EI, Kosman DJ (2003) Targeted suppression of the ferroxidase and iron trafficking activities of the multicopper oxidase Fet3p from *Saccharomyces cerevisiae*. J Biol Inorg Chem 8: 611-620.

41. Taylor AB, Stoj CS, Ziegler L, Kosman DJ, Hart PJ (2005) The copper-iron connection in biology: structure of the metallo-oxidase Fet3p. Proc Natl Acad Sci U S A 102: 15459-15464.

42. Stoj CS, Augustine AJ, Zeigler L, Solomon EI, Kosman DJ (2006) Structural basis of the ferrous iron specificity of the yeast ferroxidase, Fet3p. Biochemistry 45: 12741-12749.

43. MacDonald J, Suzuki H, Master ER (2012) Expression and regulation of genes encoding lignocellulose-degrading activity in the genus *Phanerochaete*. Appl Microbiol Biotechnol 94: 339-351.

44. Zolciak A, Kornillowicz-Kowalska T, Sierota Z, Iglik H (2008) Enzymatic activity of *Phlebiopsis gigantea* isolates. Acta Mycol 4: 41-48.

45. Mgbeahuruke AC, Sun H, Fransson P, Kasanen R, Daniel G, et al. (2011) Screening of *Phlebiopsis gigantea* isolates for traits associated with biocontrol of the conifer pathogen *Heterobasidion annosum*. Biol Control 57: 118-129.

46. Canessa P, Munoz-Guzman F, Vicuna R, Larrondo LF (2012) Characterization of PIR1, a GATA family transcription factor involved in iron responses in the white-rot fungus *Phanerochaete chrysosporium*. Fungal Genet Biol 49: 626-634.

47. Mei B, Budde AD, Leong SA (1993) *sid1*, a gene initiating siderophore biosynthesis in *Ustilago maydis*: molecular characterization, regulation by iron, and role in phytopathogenicity. Proc Natl Acad Sci U S A 90: 903-907.

48. Akileswaran L, Brock BJ, Cereghino JL, Gold MH (1999) 1,4-benzoquinone reductase from *Phanerochaete chrysosporium*: cDNA cloning and regulation of expression. Appl Environ Microbiol 65: 415-421.

49. Tanaka H, Itakura S, Enoki A (1999) Hydroxyl radical generation by an extracellular low-molecular weight substance and phenol oxidase activity during wood degradation by the white-rot basidiomycete *Phanerochaete chrysosporium*. Holzforschung 52: 21-28.

50. Aspeborg H, Coutinho PM, Wang Y, Brumer H, 3rd, Henrissat B (2012) Evolution, substrate specificity and subfamily classification of glycoside hydrolase family 5 (GH5). BMC Evol Biol 12: 186.

51. Quinlan RJ, Sweeney MD, Lo Leggio L, Otten H, Poulsen JC, et al. (2011) Insights into the oxidative degradation of cellulose by a copper metalloenzyme that exploits biomass components. Proc Natl Acad Sci U S A 108: 15079-15084.

52. Westereng B, Ishida T, Vaaje-Kolstad G, Wu M, Eijsink VG, et al. (2011) The putative endoglucanase PcGH61D from *Phanerochaete chrysosporium* is a metal-dependent oxidative enzyme that cleaves cellulose. PLoS ONE 6: e27807.

53. Li X, Beeson WTt, Phillips CM, Marletta MA, Cate JH (2012) Structural basis for substrate targeting and catalysis by fungal polysaccharide monooxygenases. Structure 20: 1051-1061.

54. van Helden J, Rios AF, Collado-Vides J (2000) Discovering regulatory elements in non-coding sequences by analysis of spaced dyads. Nucleic Acids Res 28: 1808-1818.

55. Sono M, Roach MP, Coulter ED, Dawson JH (1996) Heme-containing oxygenases. Chem Rev 96: 2841-2888.

56. Syed K, Yadav JS (2012) P450 monooxygenases (P450ome) of the model white rot fungus *Phanerochaete chrysosporium*. Crit Rev Microbiol 38: 339-363.

57. Cresnar B, Petric S (2011) Cytochrome P450 enzymes in the fungal kingdom. Biochim Biophys Acta 1814: 29-35.

58. Syed K, Doddapaneni H, Subramanian V, Lam YW, Yadav JS (2010) Genome-to-function characterization of novel fungal P450 monooxygenases oxidizing polycyclic aromatic hydrocarbons (PAHs). Biochem Biophys Res Commun 399: 492-497.

59. Syed K, Kattamuri C, Thompson TB, Yadav JS (2011) Cytochrome b(5) reductase-cytochrome b(5) as an active P450 redox enzyme system in *Phanerochaete chrysosporium*: atypical properties and in vivo evidence of electron transfer capability to CYP63A2. Arch Biochem Biophys 509: 26-32.

60. Syed K, Porollo A, Lam YW, Yadav JS (2011) A fungal P450 (CYP5136A3) capable of oxidizing polycyclic aromatic hydrocarbons and endocrine disrupting alkylphenols: role of Trp(129) and Leu(324). PLoS One 6: e28286.

61. Guo S, Bhattacharjee JK (2003) Molecular characterization of the *Candida albicans* LYS5 gene and site-directed mutational analysis of the PPTase (Lys5p) domains for lysine biosynthesis. FEMS Microbiol Lett 224: 261-267.

62. Forseth RR, Amaike S, Schwenk D, Affeldt KJ, Hoffmeister D, et al. (2013) Homologous NRPS-like gene clusters mediate redundant small-molecule biosynthesis in *Aspergillus flavus*. Angew Chem Int Ed Engl 52: 1590-1594.

63. Lackner G, Misiek M, Braesel J, Hoffmeister D (2012) Genome mining reveals the evolutionary origin and biosynthetic potential of basidiomycete polyketide synthases. Fungal Genet Biol 49: 996-1003.

64. Linder MB, Szilvay GR, Nakari-Setälä T, Penttilä ME (2005) Hydrophobins: the protein-amphiphiles of filamentous fungi. FEMS Microbiol Rev 29: 877-896.

65. Mgbeahuruike AC, Karlsson M, Asiegbu FO (2012) Differential expression of two hydrophobin genes (*Pgh1* and *Pgh2*) from the biological control agent *Phlebiopsis gigantea*. Fungal Biol 116: 620-629.

66. Kershaw MJ, Talbot NJ (1998) Hydrophobins and repellents: proteins with fundamental roles in fungal morphogenesis. Fungal Genet Biol 23: 18-33.

67. Kubicek CP, Baker S, Gamauf C, Kenerley CM, Druzhinina IS (2008) Purifying selection and birth-and-death evolution in the class II hydrophobin gene families of the ascomycete *Trichoderma/Hypocrea*. BMC Evol Biol 8: 4.

68. Kovalchuk A, Driessen AJ (2010) Phylogenetic analysis of fungal ABC transporters. BMC Genomics 11: 177.

69. Gupta A, Chattoo BB (2008) Functional analysis of a novel ABC transporter ABC4 from *Magnaporthe grisea*. FEMS Microbiol Lett 278: 22-28.

70. Mendoza-Cozatl DG, Zhai Z, Jobe TO, Akmakjian GZ, Song WY, et al. (2010) Tonoplast-localized Abc2 transporter mediates phytochelatin accumulation in vacuoles and confers cadmium tolerance. J Biol Chem 285: 40416-40426.

71. Wang Y, Lim L, DiGuistini S, Robertson G, Bohlmann J, et al. (2013) A specialized ABC efflux transporter GcABC-G1 confers monoterpene resistance to *Grosmannia clavigera*, a bark beetle-associated fungal pathogen of pine trees. New Phytol 197: 886-898.

72. Vanden Wymelenberg A, Gaskell J, Mozuch MD, Sabat G, Ralph J, et al. (2010) Comparative transcriptome and secretome analysis of wood decay fungi *Postia placenta* and *Phanerochaete chrysosporium*. Appl Environ Microbiol 76: 3599-3610.

73. Ryu JS, Shary S, Houtman CJ, Panisko EA, Korripally P, et al. (2011) Proteomic and functional analysis of the cellulase system expressed by *Postia placenta* during brown rot of solid wood. Appl Environ Microbiol 77: 7933-7941.

74. Vanden Wymelenberg A, Gaskell J, Mozuch MD, Kersten P, Sabat G, et al. (2009) Transcriptome and secretome analysis of *Phanerochaete chrysosporium* reveal complex patterns of gene expression. Appl Environ Microbiol 75: 4058-4068.

75. Vanden Wymelenberg A, Sabat G, Martinez D, Rajangam AS, Teeri TT, et al. (2005) The *Phanerochaete chrysosporium* secretome: database predictions and initial mass spectrometry peptide identifications in cellulose-grown medium. J Biotechnol 118: 17-34.

76. Nesvizhskii AI, Keller A, Kolker E, Aebersold R (2003) A statistical model for identifying proteins by tandem mass spectrometry. Anal Chem 75: 4646-4658.

77. Ishihama Y, Oda Y, Tabata T, Sato T, Nagasu T, et al. (2005) Exponentially modified protein abundance index (emPAI) for estimation of absolute protein amount in proteomics by the number of sequenced peptides per protein. Mol Cell Proteomics 4: 1265-1272.

78. Gutierrez A, del Rio JC, Gonzalez-Vila FJ, Martin F (1998) Analysis of lipophilic extractives from wood and pitch deposits by solid-phase extraction and gas chromatography. J Chromatogr 823: 449-455.
